# CSTAP: An Event-Conditioned Protocol for Toxicological Activation Potential in Coastal Sediments

**DOI:** 10.3390/toxics14080687

**Published:** 2026-08-03

**Authors:** Roberta Somma, Sebastiano Ettore Spoto

**Affiliations:** 1 Department of Mathematical and Computer Sciences, Physical Sciences and Earth Sciences (MIFT), University of Messina, Viale F. Stagno d’Alcontres 31, 98166 Messina, Italy; 2 Department of Earth Sciences, University of Florence, Via Giorgio La Pira 4, 50121 Florence, Italy

**Keywords:** coastal sediments, toxicological activation potential, theoretical protocol, bioavailability, mixture toxicity, pore water, event-driven exposure, evidence adequacy, evidentiary integrity, sediment quality guidelines

## Abstract

Coastal sediments can store contaminants for decades and become exposure sources when disturbance alters partitioning, transport, or biological contact. We propose the Coastal Sediment Toxicological Activation Protocol (CSTAP), a theoretical and empirically testable protocol for organizing event-conditioned toxicological activation potential in coastal sediments. The associated Coastal Sediment Toxicological Activation Index (CSTAI) is an exploratory, semi-quantitative score intended for hypothesis generation and future calibration, not a validated toxicity endpoint or regulatory threshold. The CSTAP separates chemical burden, effective bioavailability, mixture pressure, event activation, receptor vulnerability, evidence adequacy, uncertainty priority, and procedural integrity. The primary score alone assigns the exploratory class, whereas evidence adequacy, uncertainty priority, and forensic readiness qualify interpretation without changing the class. The protocol includes benchmark selection, information-source safeguards against double counting, route-resolved receptor weighting, event and mass-conservation checks, data-tier declarations, and validation logic. Limiting-case tests, a reproducible worked example, and illustrative Mediterranean case encodings show internal coherence and reporting structure. The CSTAP is intended to guide monitoring design, scenario comparison, and calibration with paired chemistry, exposure, bioassay, and ecological-effect datasets.

## 1. Introduction

Coastal sediments are geological archives, geochemical interfaces, benthic habitats, and potential secondary sources of exposure. In ports, lagoons, estuaries, deltas, industrial embayments, and semi-enclosed basins, fine-grained and organic-rich deposits can retain metals, hydrophobic organic chemicals, per- and polyfluoroalkyl substances (PFASs), additives, and particles for decades. Retention, however, is not equivalent to toxicological relevance. A contaminant may remain stored under one set of redox, pH, salinity, organic-carbon, and hydrodynamic conditions and become accessible under another. The central assessment problem is therefore conditional: when does stored contamination become toxicologically relevant? This question is consistent with sediment-quality triad logic, equilibrium-partitioning theory, recent syntheses of trace-metal behavior in coastal sediments, and calls for more exposure-oriented sediment assessment [1,2,3,4,5,6,7,8,9].

Sediment quality guidelines (SQGs) remain essential for screening. Effect-range, consensus-based, and default guideline values provide a transparent first tier, especially when combined with weight-of-evidence reasoning and tiered sediment-management guidance [2,3,10,11,12,13,14,15,16]. Their limitation is interpretive rather than practical: exceedance does not prove toxicity, and compliance does not prove safety. Pore-water concentration, freely dissolved concentration, passive-sampler signal, diffusive gradients in thin films (DGT)-labile flux, acid-volatile sulfide and simultaneously extracted metals (AVS–SEM) balance, grain size, organic carbon, black carbon, pH, salinity, and redox state can change the exposure meaning of the same dry-weight concentration [17,18,19,20,21,22,23,24,25,26,27]. A useful protocol should therefore preserve SQGs as benchmarks while making explicit which additional evidence changes the interpretation. A gap remains for a single auditable protocol that separates burden, exposure, event forcing, receptor vulnerability, and evidential support without converting missing information into toxicity evidence.

Coastal sediments are also mixtures. Industrial and urban deposits frequently combine metals, metalloids, polycyclic aromatic hydrocarbons (PAHs), polychlorinated biphenyls (PCBs), organochlorine pesticides, tributyltin (TBT), petroleum residues, PFASs, pharmaceuticals, plastic additives, microplastics, and nanoplastic particles. Mixture terms should not be assumed to imply synergy; additivity is the conservative default unless non-additive interactions are supported by data [28,29,30,31,32,33,34,35,36,37,38,39]. PFASs, microplastics, and nanoplastics also require metric- and route-specific treatment because they are not fully commensurable with neutral hydrophobic organics or dissolved metals.

Event forcing adds another layer. Dredging, anchoring, trawling, storms, floods, sediment nourishment, port works, bioturbation, hypoxia, reoxygenation, salinity change, warming, and acidification may alter partitioning, exposure pathways, and organism contact. Resuspension can release pore water, oxidize reduced phases, desorb hydrophobic chemicals, mobilize labile metals, or dilute exposure depending on contaminant class and event geometry [40,41,42,43,44,45]. Climate-driven changes in precipitation, marine heatwaves, oxygenation, and coastal biogeochemistry strengthen the rationale for scenario-based assessment, while not turning scenario outputs into observed toxicity [45,46,47,48].

A related scenario-aware protocol for dynamic beach sediments has shown how tiered escalation, confirmatory sampling, mass-closure requirements, and scenario-specific interpretation can reduce overinterpretation of bulk measurements in radiological assessment. The present study extends an analogous methodological logic to chemical and particle-associated toxicological activation potential in coastal sediments [49].

Some sediment datasets are also used in contested settings: dredging disputes, source-attribution debates, remediation liability, damage assessment, enforcement, or post-incident reconstruction. In those cases, the calculation is only part of the evidentiary record. Sample splitting, chain of custody, blanks, preservation, archive aliquots, and documentation of destructive analysis determine whether a result can be repeated or independently reviewed [50,51,52,53,54,55]. These requirements do not modify toxicological activation potential. They support a separate forensic-readiness reporting layer for procedural defensibility.

We introduce the Coastal Sediment Toxicological Activation Protocol (CSTAP) as a theoretical methodological Article and define its associated Coastal Sediment Toxicological Activation Index (CSTAI). The aim is not to replace SQGs, bioassays, benthic surveys, weight-of-evidence assessment, or site-specific reactive-transport models. The aim is to define a transparent protocol that separates stored burden, effective bioavailability, event activation, receptor vulnerability, evidence adequacy, uncertainty priority, and procedural integrity. Throughout the manuscript, *toxicological activation potential* denotes a conditional potential inferred from declared inputs; it is not observed toxicity unless supported by independent biological endpoints. The CSTAP does not define regulatory limits, assign liability, or claim predictive validation. It is a theory-guided protocol for structured inference and future calibration.

## 2. Materials and Methods: CSTAP Design and Mathematical Specification

### 2.1. Design Principles

The CSTAP was developed as a protocol-design study in environmental toxicology and sediment assessment. Its purpose is to formalize a transparent pathway from sediment contamination to conditional toxicological activation under specified baseline or disturbance scenarios. The protocol is grounded in four principles. First, sediment toxicity is exposure-mediated: a bulk concentration becomes ecotoxicologically relevant through chemical activity, pore-water concentration, labile flux, ingestion, or trophic transfer [8,19,20,21,26,27]. Second, bioavailability is not an intrinsic constant of a contaminant; it is a state variable controlled by sediment texture, organic carbon, black carbon, sulfides, oxides, pore-water chemistry, redox potential, pH, salinity, temperature, aging, and organism activity [17,18,22,23,56,57,58]. Third, mixture toxicity must be represented explicitly, but non-additive interaction terms should remain conservative unless supported by experimental or field evidence [28,29]. Fourth, activation is event-dependent: a sediment may remain in a latent state until a disturbance alters partitioning, transport, oxygenation, stratigraphic accessibility, or biological contact [40,41,42,45,47].

The protocol therefore separates *state*, *process*, *decision*, and, where required, *forensic-readiness reporting*. State variables describe what is currently measured or inferred at a site; process functions translate state variables into bioavailability, mixture, and activation multipliers; decision classes express the resulting toxicological activation potential. This separation is necessary because identical high scores may arise from different causal structures, including high contaminant inventory, high effective bioavailability, strong disturbance forcing, receptor sensitivity, or conservative default assumptions. The distinction is consistent with the sediment quality triad tradition but extends it by placing disturbance and climate forcing within the causal structure of assessment [1,7,8]. In contested applications, the same calculation must be accompanied by a procedural record showing whether the sample can support repeat analysis, counter-analysis, or independent review. The CSTAP therefore treats forensic readiness as a reporting layer rather than as a toxicity multiplier, and CSTAI classes remain exploratory outputs until calibrated against site-resolved exposure and effect observations.

### 2.2. Protocol Scope, Assumptions, and Falsifiable Propositions

The CSTAP is a theoretical protocol, not a retrospective literature synthesis and not a validated prediction rule. Its working hypothesis is that sediment assessment becomes more interpretable and may gain predictive relevance only after calibration and external testing, when it separates stored mass, exposure-relevant availability, disturbance-driven activation, and ecological vulnerability. The protocol therefore has an explicit scope. It is intended for coastal and transitional sediments in which at least one contaminant class is measured or reliably inferred, and in which the management question concerns present toxicity evidence, latent activation potential, or event-driven activation. It is not intended to replace organism-level tests, benthic surveys, site-specific regulatory thresholds, or full reactive-transport simulations. Rather, it specifies how these lines of evidence can be combined without dimensional inconsistency and without confusing chemical storage with toxicological action.

Four working assumptions are used. First, measured dry-weight concentrations are admissible only after normalization to a benchmark or background value. Second, all process terms entering the final score must be dimensionless and non-negative. Third, process multipliers must be bounded or explicitly reported as unbounded exploratory terms. Fourth, uncertainty must be disclosed as information deficiency, not as evidence of toxic effect. It cannot by itself cause a class shift, because the exploratory CSTAI class is assigned from $\Psi_{k,e}$ alone. These assumptions make the protocol conservative but auditable: every class assignment can be traced to a measurable variable, a selected benchmark, a coefficient, or a declared evidential limitation.

The protocol yields two classes of claims that should not be conflated. Some are *mathematical or implementation invariants*: if they fail, the protocol has been implemented incorrectly or its stated admissibility conditions have been violated. Others are *empirical hypotheses*: they require future paired datasets containing chemistry, pore-water or passive-sampler exposure metrics, disturbance descriptors, toxicity tests, and benthic-community responses [7,8,10,11,19,26,27]. This distinction prevents the word *falsifiable* from being used too broadly: an invariant is checked by implementation audit, whereas an empirical hypothesis is challenged by observation.

The resulting logic is summarized in Table 1. Each row identifies the type of claim and the observation or implementation result that would force correction of the mathematical form, coefficients, data requirements, or class thresholds.

### 2.3. Index Sets, Units, and Nomenclature

The notation was selected to avoid repeated or visually similar symbols for distinct quantities. Contaminants are indexed by $i=1,\ldots ,N_{c}$, sampling sites or sediment units by $k=1,\ldots ,N_{s}$, ecological receptors by $r=1,\ldots ,N_{r}$, and disturbance scenarios by $e=1,\ldots ,N_{e}$. Bold lowercase symbols denote vectors and bold uppercase symbols denote matrices; scalar variables remain in ordinary mathematical italics. The symbol $C_{i,k}^{\mathrm{tot}}$ denotes the total dry-weight concentration of contaminant *i* in sediment at site *k*. The symbol $Q_{i}$ denotes the selected guideline or reference value for the same contaminant and unit. The dimensionless hazard ratio is $H_{i,k}=C_{i,k}^{\mathrm{tot}}/Q_{i}$. Pore-water concentration is denoted by $C_{i,k}^{\mathrm{pw}}$, the solid–water distribution coefficient by $K_{i,k}^{d}$, the bioavailability multiplier by $\beta_{i,k}$, the effective bioavailability multiplier by $\beta_{i,k}^{\mathrm{eff}}$, the mixture multiplier by $\mu_{i,k}$, the activation multiplier under scenario *e* by $\alpha_{i,k,e}$, the receptor vulnerability coefficient by $\zeta_{i,r}$, the uncertainty-priority coefficient by $U_{k}$, and the evidence-adequacy coefficient by $K_{k,e}^{\mathrm{evi}}$. Only $\Psi_{k,e}$ is mapped to the primary exploratory class; $U_{k}$, $K_{k,e}^{\mathrm{evi}}$, and ${\mathrm{FIG}}_{k}$ are reporting layers. The essential indices and concentration variables are listed in Table 2.

The symbol set was additionally audited for semantic exclusivity. In particular, Greek letters are not treated as interchangeable generic multipliers: β is reserved for bioavailability, μ for mixture pressure, α for activation, ζ for receptor vulnerability, *f* for mobilizable fractions, η for release efficiencies, and ξ for dilution. This convention is intentionally stricter than common environmental-index notation because the CSTAP combines geochemistry, transport physics, mixture toxicology, and decision classes in the same equation. Reusing visually similar symbols for unrelated processes would make the protocol difficult to audit and would increase the chance of implementation errors. The core multipliers, weights, and score variables are summarized in Table 3.

Extended symbol-family, exposure-route, transport, kinetic, thermodynamic, oxygenation, and chemical-gate notation tables are provided as Supplementary Tables S1–S4.

### 2.4. Benchmark Hierarchy, Censored Values, and Replicate Aggregation

Because $H_{i,k}$ is the entry point through which chemical burden enters the CSTAI, the reference value $Q_{i}$ must be selected before scoring and must not be adjusted after observing the desired class. The CSTAP therefore uses an explicit benchmark hierarchy (Table 4). The selected benchmark must use the same concentration basis as $C_{i,k}^{\mathrm{tot}}$, or a documented conversion must be applied. If a concentration is reported on a wet-weight basis, the admissible dry-weight conversion is

(1) $$C_{i,k,j}^{\mathrm{dw}}=\frac{C_{i,k,j}^{\mathrm{ww}}}{1-w_{k,j}^{\mathrm{moist}}},\;0\leq w_{k,j}^{\mathrm{moist}}<1,$$

where *j* indexes analytical replicates, $C_{i,k,j}^{\mathrm{dw}}$ is the dry-weight concentration, $C_{i,k,j}^{\mathrm{ww}}$ is the wet-weight concentration, and $w_{k,j}^{\mathrm{moist}}$ is the water mass fraction of the corresponding sample. Concentrations that cannot be placed on a common dry-weight, grain-size, and benchmark basis do not pass the dimensional gate and should not be used for a numerical exploratory CSTAI class.

Left-censored analytical results must also be encoded explicitly. If a replicate is below the detection or reporting limit, CSTAP treats the admissible concentration as an interval,

(2) $$0\leq C_{i,k,j}^{\mathrm{dw}}<L_{i,k,j}^{\det},$$

where $L_{i,k,j}^{\det}$ is the detection or reporting limit. For deterministic screening, the substituted value is

(3) $${\hat{C^{\mathrm{dw}}}}_{i,k,j}\left(f^{\mathrm{cen}}\right)=f^{\mathrm{cen}}\;L_{i,k,j}^{\det},\;f^{\mathrm{cen}}\in \left\{0,0.5,1\right\}.$$

The value $f^{\mathrm{cen}}=0.5$ is a transparent midpoint convention, not a statistical estimate. Therefore, a CSTAP application with censored data should report the interval of possible classes generated by $f^{\mathrm{cen}}=0$, 0.5, and 1 or should apply a censored-data method when the dataset is large enough to justify distributional modelling [59]. This rule prevents non-detects from being silently converted into false absence or exaggerated burden.

When replicate measurements exist for a site–contaminant pair, the reported concentration should be a robust central value rather than a single arbitrary replicate. The default CSTAP aggregation is

(4) $${\tilde{C}}_{i,k}={\mathrm{median}}_{j=1,\ldots ,n_{i,k}}\left({\hat{C^{\mathrm{dw}}}}_{i,k,j}\right),$$

with a robust spread estimate

(5) $$s_{i,k}^{\mathrm{rob}}=0.7413\left[Q_{0.75}\left({\hat{C^{\mathrm{dw}}}}_{i,k,j}\right)-Q_{0.25}\left({\hat{C^{\mathrm{dw}}}}_{i,k,j}\right)\right],$$

when enough replicates are available. The factor 0.7413 converts the interquartile range to a normal-equivalent standard deviation. If $n_{i,k}<4$, the replicate range should be reported instead of $s_{i,k}^{\mathrm{rob}}$. The chemical burden used in the main score is then $H_{i,k}={\tilde{C}}_{i,k}/Q_{i}$, whereas $s_{i,k}^{\mathrm{rob}}$ enters the uncertainty and sensitivity layer. This separation is important: analytical variability should widen uncertainty, but it should not be confused with a mechanistic increase in toxicological activation potential.

### 2.5. Dimensional Closure and Physical Admissibility

The decision layer of the CSTAP is intentionally dimensionless. This is not a cosmetic choice; it is required if chemical, hydrodynamic, thermodynamic, and ecological terms are to be combined without violating dimensional homogeneity. The only quantities carrying physical units are measured state variables, such as $C_{i,k}^{\mathrm{tot}}$, $C_{i,k}^{\mathrm{pw}}$, $K_{i,k}^{d}$, $\tau_{b,k,e}$, ${\mathrm{pH}}_{k,e}$, ${\mathrm{Sal}}_{k,e}$, and $E_{k,e}^{h}$. Each of these variables is converted into a ratio, multiplier, or weight before entering $\Theta_{k,e}$. The hazard ratio $H_{i,k}$ compares sediment concentration with the selected benchmark $Q_{i}$; $\beta_{i,k}^{\mathrm{eff}}$ compares exposure evidence with a pore-water or labile benchmark after the information-source safeguard; $\mu_{i,k}$ compares mixture pressure with additivity; $\alpha_{i,k,e}$ compares event-forced exposure with baseline exposure; and $V_{i,k,e}^{\mathrm{rt}}$ is a route-resolved receptor vulnerability coefficient, with $V_{i,k}^{\mathrm{rec}}$ retained as the unrouted fallback. The uncertainty coefficient $U_{k}$ is dimensionless but is not part of the primary activation score; it is reported as a separate management-priority modifier. Consequently, the summand in Equation (83) is dimensionless, and both $\Theta_{k,e}$ and $\Psi_{k,e}$ are dimensionless.

Table 5 summarizes the admissible ranges used by the default implementation. These bounds are not biological effect thresholds; they are mathematical and physical constraints that prevent nonsensical calculations, such as negative porosity, negative exposure, or unsupported unbounded synergy.

A compact way to express the protocol state is

(6) $$\begin{array}{cc} x_{k,e}=( & {\left\{C_{i,k}^{\mathrm{tot}}\right\}}_{i=1}^{N_{c}},{\left\{K_{i,k}^{d}\right\}}_{i=1}^{N_{c}},\varepsilon_{k},\rho_{b,k},f_{k}^{\mathrm{oc}},f_{k}^{\mathrm{bc}},{\mathrm{AVS}}_{k},\sum {\mathrm{SEM}}_{k},\\ \; & {\mathrm{pH}}_{k,e},{\mathrm{Sal}}_{k,e},E_{k,e}^{h},\tau_{b,k,e},\tau_{c,k},w_{k}^{\mathrm{rec}},\nu^{\mathrm{rt}}). \end{array}$$

where $w_{k}^{\mathrm{rec}}=\left(\lambda_{1,k}^{\mathrm{rec}},\ldots ,\lambda_{N_{r},k}^{\mathrm{rec}}\right)$ is the receptor-weight vector, and $\nu^{\mathrm{rt}}$ collects receptor-specific route weights. The CSTAP maps this state vector into a scalar score through a composition of admissible functions,

(7) $$\Psi_{k,e}=M\left[x_{k,e};\vartheta \right],$$

where ϑ denotes parameters such as sorption coefficients, interaction coefficients, activation sensitivities, receptor sensitivities, and class thresholds. The mapping M is physically admissible if four conditions are met: all concentrations and densities are non-negative; porosity lies in $0<\varepsilon_{k}<1$; multipliers are bounded below by zero; and receptor weights satisfy $\sum_{r}\lambda_{r,k}^{\mathrm{rec}}=1$, while route weights satisfy $\sum_{g\in G^{\exp}}\nu_{g,r}^{\mathrm{rt}}=1$. These constraints make the CSTAP a constrained physical protocol rather than an unconstrained scoring exercise.

The non-dimensional structure also makes the protocol transferable across sites. A mercury-rich harbour, a PAH-rich industrial lagoon, a PFAS-affected estuary, and a microplastic-retaining coastal embayment may have incompatible analytical units and exposure pathways, but they can be compared at the level of normalized burden, normalized exposure, normalized activation, and normalized receptor vulnerability. This is the level at which transparent site comparison is usually required, and it is also the level at which uncertainty can be stated without hiding the incompleteness of the underlying measurements [6,7,13].

The symbol ${\mathrm{TU}}_{i,k,r}$ denotes the toxic unit (TU) for contaminant *i*, site *k*, and receptor *r*; its receptor-specific definition is introduced in the mixture module below.

This comparability is procedural, not a claim of universal commensurability. Molecular contaminants, ionizable PFASs, engineered or weathered nanomaterials, and microplastic particles do not share the same exposure metric, partitioning model, or receptor-contact mechanism. The CSTAP therefore requires particle-associated contaminants to be treated through route-specific and metric-specific variables, such as particle number, polymer type, size fraction, surface area, additive content, ingestion route, or co-contaminant flux, rather than through a single organic-carbon-normalized concentration. If these metrics are unavailable, the particulate module should be reported qualitatively, with reduced evidence adequacy, and should not be interpreted as quantitatively equivalent to metal or hydrophobic-organic modules [30,32,38,60,61].

### 2.6. Thermodynamic Closure: From Activity Gradients to Exposure Potential

The bioavailability module requires a physical bridge between a measured concentration and a biologically meaningful exposure. The CSTAP uses chemical activity as that bridge whenever the necessary information is available. To avoid conflict with the mixture multiplier $\mu_{i,k}$, the molar chemical potential is denoted here by $\varphi_{i,k}^{\mathrm{chem}}$ rather than by the conventional symbol μ. For contaminant *i* at site *k*,

(8) $$\varphi_{i,k}^{\mathrm{chem}}=\varphi_{i}^{\circ }+R_{g}T_{k,0}^{\mathrm{abs}}\ln a_{i,k},$$

where $a_{i,k}$ is thermodynamic activity, $R_{g}$ is the gas constant, and $T_{k,0}^{\mathrm{abs}}$ is the baseline absolute temperature. The dissipative flux associated with an activity gradient may be written in Onsager form as

(9) $$J_{i,k}^{\mathrm{act}}=-L_{i,k}^{\mathrm{Ons}}\nabla \varphi_{i,k}^{\mathrm{chem}}=-L_{i,k}^{\mathrm{Ons}}R_{g}T_{k,0}^{\mathrm{abs}}\nabla \ln a_{i,k},$$

where $L_{i,k}^{\mathrm{Ons}}$ is a positive phenomenological transport coefficient. Equation (9) provides the thermodynamic reason why freely dissolved concentrations, passive-sampler concentrations, and labile fluxes can be more mechanistic exposure metrics than total dry-weight concentration for many contaminants: organisms and phases respond to gradients in chemical potential or activity, not to stored mass alone [8,20,21,62,63].

For neutral hydrophobic organic contaminants, $a_{i,k}$ can be approximated by $C_{i,k}^{\mathrm{free}}/C_{i}^{\mathrm{sat}}$. For ionic contaminants, $a_{i,k}=\nu_{i,k}^{\mathrm{act}}c_{i,k}/c_{i}^{\circ }$, where $\nu_{i,k}^{\mathrm{act}}$ is an activity-coefficient correction, $c_{i,k}$ is molar concentration, and $c_{i}^{\circ }$ is a reference molarity. The CSTAP therefore defines a general dimensionless exposure potential,

(10) $$X_{i,k}=\max\left[0,\log_{10}\left(\frac{a_{i,k}+a_{i}^{\min}}{a_{i}^{\mathrm{ref}}+a_{i}^{\min}}\right)\right],$$

where $a_{i}^{\mathrm{ref}}$ is an activity-based or pore-water reference level, and $a_{i}^{\min}$ is a small positive constant used only to regularize very low activities. When activity is measured or inferred robustly, Equation (10) can replace the concentration ratio inside Equation (36). When activity is unavailable, CSTAP reverts to pore-water, passive-sampling, DGT, AVS–SEM, or categorical proxies. This hierarchy preserves mechanistic rigor without excluding data-poor sites.

For redox-sensitive contaminants, activity also depends on electron activity and proton activity. A generic reduction half-reaction

(11) $${\mathrm{Ox}}_{i}+n_{i}^{e}e^{-}+m_{i}^{H}H^{+}⇌{\mathrm{Red}}_{i}$$

implies the Nernst relation

(12) $$E_{k,e}^{h}=E_{i}^{\circ }-\frac{R_{g}T_{k,e}^{\mathrm{abs}}}{n_{i}^{e}F_{F}}\ln\left(\frac{a_{\mathrm{Red},i,k,e}}{a_{\mathrm{Ox},i,k,e}\;a_{H,k,e}^{m_{i}^{H}}}\right),\;a_{H,k,e}=10^{-{\mathrm{pH}}_{k,e}}.$$

Equations (11) and (12) are not intended to replace site-specific speciation codes. They provide the mathematical reason why pH and redox are treated as coupled state variables in the CSTAP: a change in $E_{k,e}^{h}$ is toxicologically relevant only when it changes the activity, solubility, methylation potential, or sorption state of the contaminant class under consideration [17,45,64,65,66].

### 2.7. Three-State Conceptual Representation

**Figure 1:**
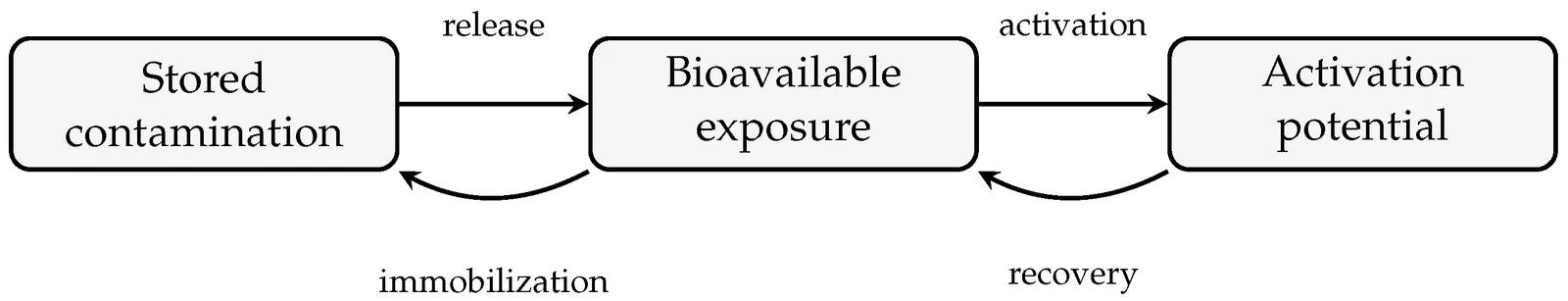
Three-state representation underlying CSTAP. The protocol separates contaminant storage, bioavailable exposure, and event-driven activation potential.

The CSTAP defines three sediment states. *Stored contamination* is the presence of contaminant mass in sediment solids, measured by $C_{i,k}^{\mathrm{tot}}$, without assuming immediate biological accessibility. *Bioavailable exposure* is the fraction or flux that can interact with organisms through pore water, ingestion, membrane transfer, or trophic pathways, represented by $C_{i,k}^{\mathrm{pw}}$, freely dissolved concentration, labile DGT flux, or an equivalent exposure proxy. *Toxicological activation potential* is the increase in exposure or effect potential following a perturbation that changes partitioning, transport, speciation, or biological contact; it is not observed toxicity unless independent biological endpoints support that interpretation. These states are ordered but not irreversible: deposition, burial, aging, and sulfide formation may move a system toward storage, whereas resuspension, oxidation, acidification, hypoxia, and bioturbation may move it toward activation [17,18,23,40,42,45]. The three-state representation is shown in Figure 1.

### 2.8. Latent-State Dynamics of Toxicological Activation Potential

The three-state representation can also be written as a minimal latent-state process. This formulation is useful because it separates the conceptual identity of a state from the measurements used to infer it. Let

(13) $$Z_{k,e}^{\mathrm{sed}}\left(t\right)\in \left\{0,1,2\right\}$$

denote the latent condition of sediment unit *k* under scenario *e* at time *t*, where 0 denotes stored contamination, 1 denotes bioavailable exposure, and 2 denotes an activation-potential state. Let

(14) $$p_{k,e}\left(t\right)=\left[p_{k,e}^{0}\left(t\right),p_{k,e}^{1}\left(t\right),p_{k,e}^{2}\left(t\right)\right]$$

be the row vector of state probabilities. A continuous-time diagnostic transition representation is

(15) $$\frac{dp_{k,e}}{dt}=p_{k,e}Q_{k,e},$$

with generator

(16) $$Q_{k,e}=\left(\begin{array}{ccc} -q_{k,e}^{01} & q_{k,e}^{01} & 0\\ q_{k,e}^{10} & -q_{k,e}^{10}-q_{k,e}^{12} & q_{k,e}^{12}\\ 0 & q_{k,e}^{21} & -q_{k,e}^{21} \end{array}\right).$$

Here, $q_{k,e}^{01}$ is the transition intensity from storage to bioavailable exposure, $q_{k,e}^{12}$ is the transition intensity from bioavailable exposure to the activation-potential state, $q_{k,e}^{10}$ represents immobilization, burial, or aging, and $q_{k,e}^{21}$ represents recovery, dilution, or management-driven reduction of active exposure. The CSTAP does not require the rates to be fitted in routine applications; rather, the state-space form defines a rigorous dynamic interpretation for the multipliers already used in the CSTAI. A parsimonious link is obtained only after the variables in the exponents have been made dimensionless. For partitioning, CSTAP uses the log-ratio diagnostic

(17) $${\bar{K}}_{k}^{d,*}=\sum_{i=1}^{N_{c}}w_{i,k}^{K}\log_{10}\left(\frac{K_{i,k}^{d}+K_{i}^{0}}{K_{i}^{d,\mathrm{ref}}+K_{i}^{0}}\right),\;\sum_{i}w_{i,k}^{K}=1,$$

where $K_{i}^{d,\mathrm{ref}}$ is a declared reference partitioning coefficient, and $K_{i}^{0}$ is a small positive stabilizer with the same units as $K_{i,k}^{d}$. The transition rates are then written as

(18) $$\begin{array}{cc} q_{k,e}^{01} & =q_{0}^{01}\;\exp\left(a_{\beta }\;\left[{\bar{\beta }}_{k}-1\right]-a_{\mathrm{imm}}\;{\bar{K}}_{k}^{d,*}\right), \end{array}$$

(19) $$\begin{array}{cc} q_{k,e}^{12} & =q_{0}^{12}\;\exp\left(a_{\alpha }\;\left[{\bar{\alpha }}_{k,e}-1\right]+a_{\mu }\;\left[{\bar{\mu }}_{k}-1\right]-a_{\mathrm{dil}}\;\chi_{k,e}^{\mathrm{dil}}\right), \end{array}$$

(20) $$\begin{array}{cc} q_{k,e}^{10} & =q_{0}^{10}\;\exp\left(a_{K}\;{\bar{K}}_{k}^{d,*}+a_{\mathrm{bur}}\;\chi_{k,e}^{\mathrm{bur}}\right), \end{array}$$

(21) $$\begin{array}{cc} q_{k,e}^{21} & =q_{0}^{21}\;\exp\left(a_{\mathrm{rec}}\;\chi_{k,e}^{\mathrm{rec}}\right), \end{array}$$

where barred quantities denote contaminant-weighted dimensionless averages, $\chi_{k,e}^{\mathrm{dil}}$ is a dilution or flushing descriptor, $\chi_{k,e}^{\mathrm{bur}}$ is a burial or capping descriptor, and $\chi_{k,e}^{\mathrm{rec}}$ represents recovery or intervention. All coefficients $a_{•}$ are dimensionless and non-negative when the corresponding process increases the indicated rate. The centered terms ${\bar{\beta }}_{k}-1$, ${\bar{\alpha }}_{k,e}-1$, and ${\bar{\mu }}_{k}-1$ make the unit values of the CSTAP multipliers neutral: when bioavailability, activation, and mixture pressure are all at their screening baselines, the corresponding transition rates return to $q_{0}^{01}$ and $q_{0}^{12}$ apart from partitioning or dilution terms. The signs in Equations (18)–(21) preserve the physical meaning of the transitions: bioavailability increases movement from storage to exposure, activation forcing increases movement from exposure to higher activation potential, strong partitioning and burial increase return to storage, and remediation increases return from activation potential toward exposure or recovery.

The latent-state representation also clarifies how the CSTAI can be tested against time-resolved observations. If toxicity tests, pore-water concentrations, or benthic responses are measured before and after a disturbance, the observed sequence can be compared with the predicted increase in $p_{k,e}^{2}\left(t\right)$. A protocol failure would occur if $\Psi_{k,e}$ and $p_{k,e}^{2}$ consistently moved in opposite directions under controlled disturbance, after measurement error and receptor mismatch have been accounted for. This makes the transition from contamination to activation not only a metaphor but a falsifiable dynamic hypothesis.

### 2.9. Sediment–Pore-Water Mass Balance and the Bioavailability Multiplier

The first mathematical layer of the CSTAP links total sediment concentration to pore-water exposure through a control-volume balance. Consider a sediment volume $V_{k}$ with pore-water volume $\varepsilon_{k}V_{k}$ and dry solid mass $\rho_{b,k}V_{k}$. For contaminant *i*, let $C_{i,k}^{\mathrm{sol}}$ be the concentration associated with sediment solids and $C_{i,k}^{\mathrm{pw}}$ be the pore-water concentration. The total concentration normalized to dry sediment mass is

(22) $$C_{i,k}^{\mathrm{tot}}=C_{i,k}^{\mathrm{sol}}+\frac{\varepsilon_{k}}{\rho_{b,k}}C_{i,k}^{\mathrm{pw}}.$$

If local linear partitioning is an acceptable diagnostic approximation,

(23) $$C_{i,k}^{\mathrm{sol}}=K_{i,k}^{d}C_{i,k}^{\mathrm{pw}},$$

then substitution of Equation (23) into Equation (22) gives

(24) $$C_{i,k}^{\mathrm{pw}}=\frac{C_{i,k}^{\mathrm{tot}}}{K_{i,k}^{d}+\varepsilon_{k}/\rho_{b,k}}.$$

Equation (24) shows why equal bulk concentrations may imply unequal exposures: sediments with lower effective $K_{i,k}^{d}$, higher pore-water volume per unit dry mass, or disturbance-enhanced desorption can yield higher $C_{i,k}^{\mathrm{pw}}$ for the same $C_{i,k}^{\mathrm{tot}}$ [5,8,19,20,21].

Equation (24) also gives a useful dimensionless diagnostic, which is the pore-water mass fraction:

(25) $$\pi_{i,k}^{\mathrm{pw}}=\frac{\left(\varepsilon_{k}/\rho_{b,k}\right)C_{i,k}^{\mathrm{pw}}}{C_{i,k}^{\mathrm{tot}}}=\frac{\varepsilon_{k}/\rho_{b,k}}{K_{i,k}^{d}+\varepsilon_{k}/\rho_{b,k}}.$$

The ratio $\pi_{i,k}^{\mathrm{pw}}$ is not a toxicity metric by itself, because organisms may be exposed by ingestion and because sorbed phases may desorb during gut passage or resuspension. It is nevertheless a physically transparent indicator of how much of the stored mass is expected to reside in pore water under the assumed partitioning approximation. Values near zero indicate strong solid-phase retention, whereas larger values identify contaminants and sediments for which pore-water exposure cannot be neglected. For hydrophobic organic contaminants, the freely dissolved concentration $C_{i,k}^{\mathrm{free}}$ is often more directly connected to chemical activity and diffusive uptake than $C_{i,k}^{\mathrm{tot}}$, which is why passive-sampling approaches have become central in contemporary sediment assessment [20,21,27,67,68].

For nonionic hydrophobic organic contaminants, the CSTAP estimates $K_{i,k}^{d}$ as a sum of sorptive phases,

(26) $$K_{i,k}^{d}=f_{k}^{\mathrm{oc}}K_{i}^{\mathrm{oc}}+f_{k}^{\mathrm{bc}}K_{i}^{\mathrm{bc}}+K_{i,k}^{\min},$$

where $f_{k}^{\mathrm{oc}}$ is the organic-carbon mass fraction, $f_{k}^{\mathrm{bc}}$ is the black-carbon mass fraction, and $K_{i,k}^{\min}$ is a residual mineral or site-specific term. The inclusion of $f_{k}^{\mathrm{bc}}K_{i}^{\mathrm{bc}}$ is important where soot, char, coke, or combustion residues strongly bind PAHs and related hydrophobic organics, lowering chemical activity relative to total concentration [56,57,69,70].

For a neutral organic contaminant, the chemical activity $a_{i,k}^{\mathrm{org}}$ can be approximated, in a dilute system, by the ratio between freely dissolved concentration and subcooled-liquid or solubility-normalized reference concentration, where

(27) $$a_{i,k}^{\mathrm{org}}\approx \frac{C_{i,k}^{\mathrm{free}}}{C_{i}^{\mathrm{sat}}}.$$

Because spontaneous diffusive uptake is driven by chemical activity gradients, Equation (27) explains why two sediments with the same dry-weight PAH concentration may have different toxicological significance when their organic-carbon quality, black-carbon content, aging, and freely dissolved concentrations differ. The CSTAP therefore treats $C_{i,k}^{\mathrm{free}}$, when available, as a superior exposure metric for hydrophobic organic chemicals, while retaining $C_{i,k}^{\mathrm{tot}}$ for first-tier screening [8,20,21,27,62,63].

#### 2.9.1. Kinetic Extension and Transport Time Scales

Equilibrium partitioning is useful for screening, but coastal disturbance may occur faster than sorption, diffusion, or sediment–water exchange can equilibrate. The CSTAP therefore includes a kinetic extension for event scenarios. Let *z* be the upward vertical coordinate within the active sediment layer, and let $D_{i,k}^{\mathrm{sed}}$ denote the effective diffusion coefficient corrected for tortuosity. A minimal one-dimensional exposure balance for pore water is

(28) $$\varepsilon_{k}\frac{\partial C_{i,k,e}^{\mathrm{pw}}}{\partial t}=\frac{\partial }{\partial z}\left(\varepsilon_{k}D_{i,k}^{\mathrm{sed}}\frac{\partial C_{i,k,e}^{\mathrm{pw}}}{\partial z}\right)-\rho_{b,k}K_{i,k}^{d}k_{i,k}^{\mathrm{des}}\left[C_{i,k,e}^{\mathrm{pw}}-C_{i,k}^{\mathrm{pw},\mathrm{eq}}\right]+S_{i,k,e}^{\mathrm{rxn}},$$

where $C_{i,k}^{\mathrm{pw},\mathrm{eq}}$ is the local equilibrium concentration given by Equation (24), and $S_{i,k,e}^{\mathrm{rxn}}$ is a net source or sink term for transformation, complexation, precipitation, dissolution, or biodegradation. The factor $\rho_{b,k}K_{i,k}^{d}$ converts first-order relaxation in the sorbed pool into a pore-water mass-balance term, preserving dimensional homogeneity. The sign of the relaxation term is chosen so that pore water approaches $C_{i,k}^{\mathrm{pw},\mathrm{eq}}$ in the absence of transport and reaction. Equation (28) is deliberately minimal: it does not replace full diagenetic or reactive-transport models, but it identifies the time scales that determine whether equilibrium assumptions are admissible during an event [62,64,65,71].

For an event of duration $\Delta t_{e}$ and active length scale $\ell_{k,e}^{\mathrm{mix}}$, three dimensionless groups are especially informative:

(29) $${\mathrm{Da}}_{i,k,e}^{\mathrm{des}}=k_{i,k}^{\mathrm{des}}\Delta t_{e},\;{\mathrm{Pe}}_{i,k,e}^{\mathrm{adv}}=\frac{|w_{k,e}^{\mathrm{adv}}|\ell_{k,e}^{\mathrm{mix}}}{D_{i,k}^{\mathrm{sed}}},\;{\mathrm{Bi}}_{i,k,e}^{\mathrm{sw}}=\frac{k_{i,k,e}^{\mathrm{sw}}\ell_{k,e}^{\mathrm{mix}}}{D_{i,k}^{\mathrm{sed}}},$$

where $w_{k,e}^{\mathrm{adv}}$ is a pore-water advective velocity scale, and $k_{i,k,e}^{\mathrm{sw}}$ is an external sediment–water exchange coefficient. If ${\mathrm{Da}}_{i,k,e}^{\mathrm{des}}\gg 1$, sorption relaxation is fast relative to event duration and equilibrium partitioning may be a defensible approximation. If ${\mathrm{Da}}_{i,k,e}^{\mathrm{des}}\ll 1$, the event samples a kinetically constrained state, and exposure may be governed by desorption rate rather than by equilibrium capacity. Large ${\mathrm{Pe}}_{i,k,e}^{\mathrm{adv}}$ indicates that advection or pressure-driven exchange can exceed molecular diffusion, whereas large ${\mathrm{Bi}}_{i,k,e}^{\mathrm{sw}}$ indicates that external exchange is sufficiently rapid for internal sediment diffusion to become limiting. These quantities provide a physical audit of the activation multiplier $\alpha_{i,k,e}$: the multiplier should not be increased merely because a sediment is contaminated but because the time scales and transport pathways allow contaminant exposure to rise during the scenario.

The corresponding flux decomposition is

(30) $$J_{i,k,e}=J_{i,k,e}^{\mathrm{diff}}+J_{i,k,e}^{\mathrm{res}}+J_{i,k,e}^{\mathrm{bio}},$$

where

(31) $$J_{i,k,e}^{\mathrm{diff}}=-\varepsilon_{k}D_{i,k}^{\mathrm{sed}}{\left.\frac{\partial C_{i,k,e}^{\mathrm{pw}}}{\partial z}\right|}_{z=0},\;J_{i,k,e}^{\mathrm{res}}=F_{k,e}^{\mathrm{er}}\left(C_{i,k}^{\mathrm{sol}}+\frac{\varepsilon_{k}}{\rho_{b,k}}C_{i,k}^{\mathrm{pw}}\right).$$

The first term is a diffusive flux across the sediment–water interface; the second is the resuspension-associated release of contaminated solids and pore-water-equivalent mass; the third represents organism-mediated particle ingestion, irrigation, and sediment reworking. Equations (28)–(31) justify treating activation as a process variable rather than as an arbitrary categorical label [23,40,42,45,72].

#### 2.9.2. Ionizable Contaminants and PFAS-Sensitive Partitioning

For ionizable organic contaminants and PFASs, a single neutral-compound $K_{\mathrm{oc}}$ expression is often insufficient. The CSTAP uses the following diagnostic expansion when speciation-relevant information is available:

(32) $$K_{i,k,e}^{d}=f_{i,k,e}^{0}K_{i,k}^{d,0}+f_{i,k,e}^{-}K_{i,k}^{d,-}+f_{i,k,e}^{+}K_{i,k}^{d,+}+K_{i,k,e}^{d,\min}+K_{i,k,e}^{d,\mathrm{biofilm}},$$

where $f_{i,k,e}^{0}$, $f_{i,k,e}^{-}$, and $f_{i,k,e}^{+}$ are the neutral, anionic, and cationic fractions, respectively, and the five $K^{d}$ terms represent sorption of neutral molecules, anions, cations, mineral-associated species, and biofilm-associated species. For a monoprotic acid, the anionic fraction is

(33) $$f_{i,k,e}^{-}=\frac{1}{1+10^{pK_{a,i}-{\mathrm{pH}}_{k,e}}},\;f_{i,k,e}^{0}=1-f_{i,k,e}^{-},$$

with analogous expressions for bases and zwitterions. The purpose of Equations (32) and (33) is not to force an oversimplified PFAS model onto all sites but to prevent PFASs and ionizable contaminants from being treated as classical neutral hydrophobes. Chain length, head group, ionic strength, organic matter, mineral surfaces, protein affinity, precursor transformation, and salinity can alter mobility and uptake; the CSTAP therefore directs these compounds toward pore-water, sediment-ingestion, and trophic-transfer evidence rather than relying only on dry-weight sediment concentration [31,32,33,34,36,38,73,74].

For divalent cationic metals, the CSTAP uses an AVS–SEM diagnostic term when data are available. Let $\sum {\mathrm{SEM}}_{k}$ denote the molar sum of simultaneously extracted Cd, Cu, Ni, Pb, and Zn, and let ${\mathrm{AVS}}_{k}$ denote an acid-volatile sulfide. A sediment has low expected dissolved metal activity when $\sum {\mathrm{SEM}}_{k}\leq {\mathrm{AVS}}_{k}$, because metal sulfides are sparingly soluble under reducing conditions. The normalized excess-metal term is defined as

(34) $$X_{k}^{\mathrm{AVS}}=\frac{\max\left(0,\sum {\mathrm{SEM}}_{k}-{\mathrm{AVS}}_{k}\right)}{{\mathrm{AVS}}_{k}+f_{k}^{\mathrm{oc}}C_{\mathrm{AVS}}^{\mathrm{OC},\mathrm{ref}}+C_{\mathrm{AVS}}^{0}},$$

where $C_{\mathrm{AVS}}^{\mathrm{OC},\mathrm{ref}}$ is an organic-carbon (OC)-associated molar reference capacity, and $C_{\mathrm{AVS}}^{0}>0$ is a regularization constant with the same units as $\sum {\mathrm{SEM}}_{k}$ and ${\mathrm{AVS}}_{k}$. Equation (34) is therefore dimensionless. It is not used as a universal predictor but as a mechanistic adjustment of metal bioavailability when the relevant measurements exist [6,17,18,75].

The AVS–SEM correction is explicitly conditional on redox state. A simplified oxidation pathway for metal sulfides can be represented as

(35) $${\mathrm{MS}}_{\left(s\right)}+2\;O_{2(aq)}\to M_{\left(aq\right)}^{2+}+{\mathrm{SO}}_{4}^{2-},$$

where $M^{2+}$ denotes a divalent metal. Equation (35) is not used as a full kinetic model, but it formalizes the physical meaning of oxidizing reduced sediments during dredging or resuspension: a phase that can immobilize metals under anoxic conditions may cease to do so when exposed to oxygenated water. This is why the CSTAP treats the sign and magnitude of $\Delta_{E^{h},k,e}^{⋆}$ as contaminant-specific rather than assigning a single universal redox penalty [40,42,45,66].

The dimensionless bioavailability multiplier is then defined with a threshold-centered logistic positive part,

(36) $$\beta_{i,k}=1+\beta_{i}^{\max}\;{\left[2\sigma \;\left(\kappa_{i}^{\beta }\log_{10}\frac{C_{i,k}^{\mathrm{pw}}+c_{i}^{0}}{C_{i}^{\mathrm{ref},\mathrm{pw}}+c_{i}^{0}}\right)-1\right]}_{+},$$

where ${\left[x\right]}_{+}=\max\left(0,x\right)$, $C_{i}^{\mathrm{ref},\mathrm{pw}}$ is a pore-water effect benchmark or predicted-no-effect-concentration (PNEC)-like reference concentration, $c_{i}^{0}$ is a low-concentration numerical stabilizer, $\kappa_{i}^{\beta }$ controls transition steepness, $\beta_{i}^{\max}$ is the maximum bioavailability amplification, and $\sigma \left(x\right)=1/\left(1+e^{-x}\right)$ is the logistic function. This scaling gives $\beta_{i,k}=1$ at $C_{i,k}^{\mathrm{pw}}=C_{i}^{\mathrm{ref},\mathrm{pw}}$ and below the benchmark in screening mode, while allowing smooth amplification above the benchmark. When pore-water data are unavailable, $C_{i,k}^{\mathrm{pw}}$ can be replaced by a proxy derived from Equation (24), by passive-sampler freely dissolved concentration, by DGT-labile flux, or by categorical scoring based on sediment properties. This construction ensures that $\beta_{i,k}\geq 1$ and that the multiplier does not overstate exposure merely because the raw concentration has crossed a benchmark.

#### 2.9.3. Information-Source Safeguard Against Double Counting

Because $H_{i,k}$ already contains the total concentration $C_{i,k}^{\mathrm{tot}}$, the bioavailability term must not simply re-express the same concentration signal. The CSTAP therefore applies an information-source safeguard before $\beta_{i,k}$ is converted into the effective multiplier used in the final score. Let $I_{i,k}^{\mathrm{src}}\in \left[0,1\right]$ denote an information-source safeguard coefficient, i.e., a governance coefficient describing whether the evidence used to assign $\beta_{i,k}$ comes from a source separable from the bulk hazard ratio. It is not a formal statistical-independence test. The effective bioavailability multiplier used in CSTAI is

(37) $$\beta_{i,k}^{\mathrm{eff}}=1+I_{i,k}^{\mathrm{src}}\left(\beta_{i,k}-1\right).$$

When $\beta_{i,k}$ is based on measured pore water, passive-sampler freely dissolved concentrations, DGT fluxes, AVS–SEM, or another exposure measurement not reducible to $C_{i,k}^{\mathrm{tot}}$ alone, $I_{i,k}^{\mathrm{src}}$ may approach 1. When $\beta_{i,k}$ is inferred from equilibrium partitioning using the same $C_{i,k}^{\mathrm{tot}}$ together with measured sediment properties such as organic carbon, black carbon, porosity, or sulfides, $I_{i,k}^{\mathrm{src}}$ should be reported below 1 because part of the exposure proxy is inherited from the bulk concentration. When no information beyond $C_{i,k}^{\mathrm{tot}}$ is available, $I_{i,k}^{\mathrm{src}}=0$ and $\beta_{i,k}^{\mathrm{eff}}=1$; the assessment then remains burden-based and the missing exposure evidence is recorded through $K_{k,e}^{\mathrm{evi}}$ rather than being converted into an artificial bioavailability amplification. In screening mode, $\beta_{i,k}^{\mathrm{eff}}$ is therefore an exposure-amplification modifier relative to the burden term, not a bidirectional exposure fraction; attenuation claims require direct exposure evidence or a calibrated partitioning model.

This rule is conservative and methodologically necessary. It allows the CSTAP to use exposure measurements when they exist, while preventing the uncorrected product $H_{i,k}\beta_{i,k}$ from double counting the same concentration measurement in data-poor cases. All applications must therefore report the source of $\beta_{i,k}$, the value assigned to $I_{i,k}^{\mathrm{src}}$, and whether the final contribution uses measured exposure, partitioning-derived exposure, or categorical exposure evidence. Minimal assignments are illustrated in Table 6.

The same principle is extended to the route-resolved receptor term. The effective bioavailability multiplier, $\beta_{i,k}^{\mathrm{eff}}$, represents physicochemical availability in the sediment–pore-water system. The route-resolved term, $V_{i,k,e}^{\mathrm{rt}}$, represents receptor access, route relevance, and biological contact. If the same exposure measurement is used directly to construct $V_{i,k,e}^{\mathrm{rt}}$, then $I_{i,k}^{\mathrm{src}}$ must be reduced or $\beta_{i,k}^{\mathrm{eff}}$ must be set to 1 for that route. Thus, a measured pore-water concentration, passive-sampler concentration, DGT flux, or ingestion proxy can inform the score, but it cannot be counted twice as both physicochemical availability and receptor-route accessibility. The corresponding governance rules are listed in Table 7.

### 2.10. Mixture Pressure

CSTAP represents mixtures using two nested layers: a baseline concentration-addition term and an interaction matrix. For a receptor *r*, the toxic unit (TU) for contaminant *i* at site *k* is

(38) $${\mathrm{TU}}_{i,k,r}=\frac{E_{i,k}}{C_{i,r}^{\mathrm{eff}}},$$

where $E_{i,k}$ is an exposure metric selected for the contaminant class, such as $C_{i,k}^{\mathrm{pw}}$, freely dissolved concentration, DGT flux, or a normalized sediment concentration, and $C_{i,r}^{\mathrm{eff}}$ is a receptor-specific effect concentration. The concentration-addition pressure for receptor *r* is

(39) $${\mathrm{TU}}_{k,r}^{\sum }=\sum_{i=1}^{N_{c}}{\mathrm{TU}}_{i,k,r}.$$

When $C_{i,r}^{\mathrm{eff}}$ is unavailable, the CSTAP uses $H_{i,k}\beta_{i,k}^{\mathrm{eff}}$ as a surrogate screening contribution, while preserving an explicit uncertainty flag. This is consistent with tiered mixture assessment: more mechanistic metrics are used when effect concentrations and exposure proxies exist; otherwise conservative hazard-based surrogates are retained with transparent uncertainty [8,28,29].

Toxic-unit quantities are diagnostic in the default screening implementation. They do not replace the primary contribution unless a receptor-specific toxic-unit mode is explicitly declared. In default mode,

$$\Omega_{i,k,e}^{\mathrm{screen}}=H_{i,k}\;\beta_{i,k}^{\mathrm{eff}}\;\mu_{i,k}\;\alpha_{i,k,e}^{†}\;V_{i,k,e}^{\mathrm{rt}}.$$

In a declared receptor-specific toxic-unit mode, the additive exposure term may instead be written as

$$\Omega_{i,k,e}^{\mathrm{TU}}=\sum_{r=1}^{N_{r}}\lambda_{r,k}^{\mathrm{rec}}\;{\mathrm{TU}}_{i,k,r}\;\mu_{i,k,r}\;\alpha_{i,k,e}^{†}\;\zeta_{i,r},$$

with receptor weights, effect references, and evidence adequacy reported separately. This separation prevents ${\mathrm{TU}}^{\sum }$ and $\mu_{i,k}$ from becoming two overlapping mixture-pressure scores.

Pairwise deviations from additivity are represented by the symmetric interaction matrix $\Gamma =(\gamma_{ij})$, where $\gamma_{ij}>0$ denotes potentiation or synergy, $\gamma_{ij}=0$ denotes no interaction beyond additivity, and $\gamma_{ij}<0$ denotes antagonism or reduced joint action. The concentration-addition term in Equation (39) defines the additive baseline. The mixture multiplier $\mu_{i,k}$ is therefore a non-additive modifier, not a second additive mixture score. The multiplier applied to contaminant *i* at site *k* is

(40) $$\mu_{i,k}=\mathrm{clip}\left[1+\sum_{\begin{array}{c} j=1\\ j\neq i \end{array}}^{N_{c}}\gamma_{ij}\;\min\left(1,H_{j,k}\beta_{j,k}^{\mathrm{eff}}\right),\mu_{i}^{\min},\mu_{i}^{\max}\right],$$

where clip(x,L,U)=max[L,min(x,U)], and the admissible bounds satisfy $0<\mu_{i}^{\min}\leq 1\leq \mu_{i}^{\max}$. The strictly positive lower bound prevents negative or zero mixture multipliers from destroying the toxicological meaning of $\Omega_{i,k,e}$, $\Theta_{k,e}$, and $\Psi_{k,e}$. The clipping bounds prevent unsupported extrapolation in poorly characterized mixtures. In first-tier applications, $\gamma_{ij}=0$ and $\mu_{i,k}=1$ are the defaults for unknown pairs, and positive or negative values should be assigned only for documented co-stressors, shared modes of action, or experimentally supported interactions. Because the full pairwise matrix becomes weakly identifiable as the number of analytes grows, the CSTAP treats Γ as a sparse, evidence-dependent matrix rather than as a complete catalogue of possible synergies. Grouping by contaminant class or mode of action is preferable to populating unsupported pairwise terms. This prevents the protocol from assuming synergy as a default while still allowing interaction to be represented when justified [30,32,33].

### 2.11. Activation Triggers and Disturbance Functions

The activation multiplier $\alpha_{i,k,e}$ quantifies how scenario *e* modifies exposure for contaminant *i* at site *k*. The CSTAP decomposes activation into physical, chemical, biological-contact, and stratigraphic intensities. Physical disturbance is therefore not the only activation driver: hypoxia, anoxia, reoxygenation, acidification, salinity shifts, and redox transitions may enter through a contaminant-specific chemical activation intensity even when the mechanical disturbance term is null. The dimensionless activation intensity is

(41) $$I_{i,k,e}^{\mathrm{act}}=\chi_{i}^{\mathrm{phys}}I_{k,e}^{\mathrm{phys}}+\chi_{i}^{\mathrm{chem}}I_{i,k,e}^{\mathrm{chem}}+\chi_{i}^{\mathrm{bio}}I_{k,e}^{\mathrm{bio}}+\chi_{i}^{z}I_{k,e}^{z},$$

where all four χ coefficients are non-negative sensitivity parameters. The unbounded diagnostic form is then

(42) $${\tilde{\alpha }}_{i,k,e}=1+\Lambda_{i}\left[1-\exp\left(-I_{i,k,e}^{\mathrm{act}}\right)\right].$$

The admitted static multiplier is capped unless a site-specific calibrated unbounded exploratory form is explicitly declared:

(43) $$\alpha_{i,k,e}=\min\left\{1+\Lambda_{i},\max\left(1,{\tilde{\alpha }}_{i,k,e}\right)\right\}.$$

Thus, $\Lambda_{i}$ denotes the upper activation increment after the physical, chemical, biological-contact, and stratigraphic terms have been transformed into dimensionless intensities. The cap keeps the default screening multiplier within $\left[1,1+\Lambda_{i}\right]$. Attenuating activation values below 1 are allowed only in a separately declared calibrated attenuation mode and must not be interpreted as conservative first-tier screening.

The main free or weakly identified parameters and their admissible defaults are governed as summarized in Table 8.

For hydrodynamic or mechanical disturbance, the physical component of the activation intensity can be written as

(44) $$I_{k,e}^{\mathrm{phys}}=\max\left(0,\frac{\tau_{b,k,e}-\tau_{c,k}}{\tau_{c,k}}\right)+\delta_{k,e}^{\mathrm{drg}},\;I_{k,e}^{\mathrm{bio}}=\delta_{k,e}^{\mathrm{bio}},$$

where $\tau_{b,k,e}$ is bed shear stress, $\tau_{c,k}$ is critical erosion stress, $\delta_{k,e}^{\mathrm{drg}}$ is a dredging or mechanical disturbance term, and $\delta_{k,e}^{\mathrm{bio}}$ is a biological-contact or bioturbation term. In shallow coastal systems, $\tau_{b,k,e}$ can be estimated from current velocity or wave–current boundary-layer models. A minimal current-based expression is

(45) $$\tau_{b,k,e}=\rho_{w}C_{D}{\left(v_{k,e}^{\mathrm{bed}}\right)}^{2},$$

where $\rho_{w}$ is water density, $C_{D}$ is a drag coefficient, and $v_{k,e}^{\mathrm{bed}}$ is the near-bed velocity under scenario *e*. Equation (44) formalizes the physical forcing pathway. Chemical activation remains possible through $I_{i,k,e}^{\mathrm{chem}}$ even when $I_{k,e}^{\mathrm{phys}}=0$, so pH, salinity, redox, hypoxia, or reoxygenation are not mathematically forced to wait for bed erosion [40,41,43,44].

The chemical gating term is defined with standardized, dimensionless scenario departures:

(46) $$\Delta_{\mathrm{pH},k,e}^{⋆}=\frac{{\mathrm{pH}}_{k,e}-{\mathrm{pH}}_{k,0}}{\Delta {\mathrm{pH}}_{i}^{\mathrm{ref}}},\;\Delta_{\mathrm{sal},k,e}^{⋆}=\frac{{\mathrm{Sal}}_{k,e}-{\mathrm{Sal}}_{k,0}}{\Delta {\mathrm{Sal}}_{i}^{\mathrm{ref}}},\;\Delta_{E^{h},k,e}^{⋆}=\frac{E_{k,e}^{h}-E_{k,0}^{h}}{\Delta E_{i}^{\mathrm{ref}}},$$

where the denominators are contaminant-specific reference intervals chosen from measurement resolution, experimental calibration, or management-relevant scenario ranges. The gate is then

(47) $$g_{i,k,e}^{\mathrm{chem}}=\exp\left[-s_{i}^{\mathrm{pH}}\Delta_{\mathrm{pH},k,e}^{⋆}-s_{i}^{\mathrm{sal}}\Delta_{\mathrm{sal},k,e}^{⋆}+s_{i}^{\mathrm{red}}\Delta_{E^{h},k,e}^{⋆}\right].$$

Because all arguments of the exponential are dimensionless, Equation (47) remains dimensionally closed even when pH, salinity, and redox potential have different physical units and measurement conventions. The gate itself is positive; the coefficients $s_{i}^{\mathrm{pH}}$, $s_{i}^{\mathrm{sal}}$, and $s_{i}^{\mathrm{red}}$ are the signed sensitivity coefficients. For the activation-intensity calculation, the release-oriented chemical component is

(48) $$I_{i,k,e}^{\mathrm{chem}}={\left[\ln g_{i,k,e}^{\mathrm{chem}}\right]}_{+},$$

so $g_{i,k,e}^{\mathrm{chem}}>1$ can trigger activation without mechanical erosion, whereas $g_{i,k,e}^{\mathrm{chem}}<1$ indicates chemical attenuation or immobilization and does not reduce the conservative screening score unless calibrated attenuation mode is explicitly declared. The coefficients $s_{i}^{\mathrm{pH}}$, $s_{i}^{\mathrm{sal}}$, and $s_{i}^{\mathrm{red}}$ encode contaminant-specific sensitivity and are signed coefficients, not universal positive penalties. Positive $s_{i}^{\mathrm{red}}$ can represent oxidation-sensitive release, whereas negative $s_{i}^{\mathrm{red}}$ can represent reduction-sensitive release or oxidation-driven immobilization; alternatively, applications may split the gate into separate oxidation-sensitive and reduction-sensitive pathways. For many cationic metals, lower pH and oxidation of reduced sulfide-bearing sediment may increase dissolved or labile fractions; for other metals or redox-active systems, reducing conditions, Fe–Mn oxide dissolution, sulfide formation, methylation windows, or reoxygenation can increase or decrease exposure depending on phase assemblage and organic matter. For some organic contaminants, salinity and dissolved organic matter can shift partitioning; for PFASs, chain length, functional group, organic matter, protein affinity, and ionic strength influence mobility and biotic uptake [32,33,42,45,58]. Equation (47) is therefore not a universal speciation model but a compact, bidirectional parameterization that can be replaced by PHREEQC, the Windermere Humic Aqueous Model (WHAM), the Non-Ideal Competitive Adsorption (NICA)–Donnan model, fugacity, or contaminant-specific transport models where such models are available.

The exponential form in Equation (47) is physically motivated by equilibrium thermodynamics. For a generic redox half-reaction involving *n* electrons and *m* protons, the Nernst relation may be written as

(49) $$E^{h}=E^{0^{\prime }}+\frac{R_{g}T_{k,e}^{\mathrm{abs}}}{nF_{F}}\ln\left(\frac{a_{\mathrm{Ox}}}{a_{\mathrm{Red}}}\right)-\frac{mR_{g}T_{k,e}^{\mathrm{abs}}\ln10}{nF_{F}}\mathrm{pH},$$

where $R_{g}$ is the gas constant, $T_{k,e}^{\mathrm{abs}}$ is absolute temperature, $F_{F}$ is Faraday’s constant, and $a_{\mathrm{Ox}}/a_{\mathrm{Red}}$ is the activity ratio between oxidized and reduced forms. Rearrangement gives an exponential dependence of activity ratios on $E^{h}$ and pH. The CSTAP does not attempt to solve full speciation from Equation (49); rather, it uses the same mathematical logic to justify smooth gates for pH and redox forcing. Site-specific speciation calculations can replace the gate when thermodynamic databases and mineral phases are sufficiently constrained [6,45,58,62].

### 2.12. Temperature, Oxygenation, and Redox-Conditioned Kinetic Modifiers

Temperature is treated in the CSTAP as a process modifier rather than as an independent contaminant. Warming does not create chemical burden, but it can alter desorption rates, diffusion, organic-contaminant partitioning, microbial transformation, oxygen demand, and biological sensitivity. When a contaminant-specific kinetic parameter is available at a reference temperature, the CSTAP uses an Arrhenius-type correction,

(50) $$\phi_{i,k,e}^{T}=\exp\left[-\frac{E_{i}^{\mathrm{des}}}{R_{g}}\left(\frac{1}{T_{k,e}^{\mathrm{abs}}}-\frac{1}{T_{i}^{\mathrm{ref}}}\right)\right],$$

where $E_{i}^{\mathrm{des}}$ is an apparent activation energy for desorption, transformation, or release; $T_{k,e}^{\mathrm{abs}}$ is the absolute temperature under scenario *e*; and $T_{i}^{\mathrm{ref}}$ is the reference temperature at which the parameter was measured. The temperature-corrected desorption coefficient is

(51) $$k_{i,k,e}^{\mathrm{des},T}=k_{i,k}^{\mathrm{des}}\;\phi_{i,k,e}^{T}.$$

For screening applications that lack activation-energy estimates, the same information can be encoded through a temperature-coefficient $Q_{10}$ form,

(52) $$\phi_{i,k,e}^{T}=Q_{10,i}^{(T_{k,e}^{\mathrm{abs}}-T_{i}^{\mathrm{ref}})/10},$$

provided that the temperature interval is expressed as a temperature difference and that the adopted $Q_{10,i}$ value is reported.

For sorption equilibria, the CSTAP allows a van’t Hoff correction when the direction and magnitude of the apparent sorption enthalpy are known:

(53) $$\ln\left(\frac{K_{i,k,e}^{d,T}}{K_{i,k}^{d,\mathrm{ref}}}\right)=-\frac{\Delta h_{i}^{\mathrm{sorp}}}{R_{g}}\left(\frac{1}{T_{k,e}^{\mathrm{abs}}}-\frac{1}{T_{i}^{\mathrm{ref}}}\right),$$

where $\Delta h_{i}^{\mathrm{sorp}}$ is the apparent enthalpy associated with sediment–water distribution. Equation (53) is not applied blindly: for heterogeneous sediments, black-carbon-rich phases, aging, biofilms, and ionizable contaminants, the sign and magnitude of $\Delta h_{i}^{\mathrm{sorp}}$ may vary. The default protocol therefore uses the correction only when supported by measurements, compound-class evidence, or calibrated site-specific partitioning.

Oxygenation is represented separately from redox potential because dissolved oxygen, sulfide stability, iron–manganese cycling, organic-matter degradation, and microbial methylation are related but not identical observables. A dimensionless oxygen gate can be defined as

(54) $$g_{k,e}^{\mathrm{ox}}={\left[1+\exp\left(-\upsilon^{\mathrm{ox}}\frac{O_{k,e}^{\mathrm{diss}}-O_{k}^{\mathrm{ref}}}{\Delta O_{k}^{\mathrm{ref}}}\right)\right]}^{-1},$$

where $O_{k}^{\mathrm{ref}}$ is a site-specific oxygen reference, $\Delta O_{k}^{\mathrm{ref}}$ is a scaling interval, and $\upsilon^{\mathrm{ox}}$ controls transition sharpness. The oxygen gate selects which redox-sensitive pathway is plausible; it is not itself a universal toxicity multiplier.

A temperature- and oxygen-conditioned activation multiplier can be written, in default conservative screening mode, as

(55) $$\alpha_{i,k,e}^{\mathrm{TO}}=\min\left\{1+\Lambda_{i},\;\alpha_{i,k,e}\;\max\left[1,\left(1+\omega_{i}^{T}\left(\phi_{i,k,e}^{T}-1\right)\right)\left(1+\omega_{i}^{O}\left(g_{k,e}^{\mathrm{ox}}-g_{k,0}^{\mathrm{ox}}\right)\right)\right]\right\},$$

where $\omega_{i}^{T}$ and $\omega_{i}^{O}$ are bounded sensitivity coefficients. Equation (55) is neutral or amplifying in default screening: it cannot reduce the primary activation score, and it cannot exceed the same admissible upper bound, $1+\Lambda_{i}$, used for the static activation multiplier. If a user allows $\alpha_{i,k,e}^{\mathrm{TO}}<1$ to represent calibrated attenuation, the calculation must be explicitly labeled as calibrated attenuation mode and must not be interpreted as conservative first-tier screening. An exponential form may be used when calibrated kinetic data are available. In the default CSTAI calculation, $\alpha_{i,k,e}^{\mathrm{TO}}$ replaces $\alpha_{i,k,e}$ only when temperature and oxygen data are available or when the event scenario explicitly concerns warming, hypoxia, or reoxygenation. This optional layer is included to make the protocol compatible with climate-adapted sediment assessment without overfitting data-poor sites; it also provides a direct route for laboratory validation under controlled temperature and oxygen scenarios [7,8,42,45,47,76].

### 2.13. Resuspension, Pore-Water Release, and Exposure-Pulse Scaling

The activation multiplier $\alpha_{i,k,e}$ can be related to standard cohesive-sediment transport physics. For an erosive event, the upward sediment mass flux can be represented by a Partheniades-type law,

(56) $$F_{k,e}^{\mathrm{er}}=M_{k}^{\mathrm{er}}{\left[\frac{\tau_{b,k,e}}{\tau_{c,k}}-1\right]}_{+},\;{\left[x\right]}_{+}=\max\left(0,x\right),$$

where $M_{k}^{\mathrm{er}}$ is an erodibility coefficient and $F_{k,e}^{\mathrm{er}}$ has dimensions of mass area^−1^ time^−1^. For a mixed layer of thickness $h_{k,e}^{\mathrm{mix}}$, the suspended particulate matter (SPM) concentration obeys the reduced mass balance

(57) $$\frac{dC_{k,e}^{\mathrm{SPM}}}{dt}=\frac{F_{k,e}^{\mathrm{er}}}{h_{k,e}^{\mathrm{mix}}}-\frac{w_{k,e}^{\mathrm{set}}}{h_{k,e}^{\mathrm{mix}}}C_{k,e}^{\mathrm{SPM}},$$

where $w_{k,e}^{\mathrm{set}}$ is an effective settling velocity. Under constant forcing, Equation (57) has the solution

(58) $$C_{k,e}^{\mathrm{SPM}}\left(t\right)=C_{k,e}^{\mathrm{SPM}}\left(0\right)\exp\left(-\frac{w_{k,e}^{\mathrm{set}}t}{h_{k,e}^{\mathrm{mix}}}\right)+\frac{F_{k,e}^{\mathrm{er}}}{w_{k,e}^{\mathrm{set}}}\left[1-\exp\left(-\frac{w_{k,e}^{\mathrm{set}}t}{h_{k,e}^{\mathrm{mix}}}\right)\right].$$

Equations (56)–(58) show why activation is nonlinear in event strength. Below the critical stress, erosion is absent in the reduced model; above it, suspended mass and particle-associated exposure increase toward an event-dependent steady value. These equations also separate physical forcing from contaminant chemistry: the same hydrodynamic event can produce very different toxicological responses depending on $C_{i,k}^{\mathrm{tot}}$, $K_{i,k}^{d}$, $\pi_{i,k}^{\mathrm{pw}}$, and sediment redox state [40,41,71,72].

For a receptor exposed during a finite event of duration $\Delta t_{e}$, the CSTAP defines the dimensionless exposure-pulse number

(59) $$P_{i,k,e,r^{*}}^{\exp}=\frac{1}{\Delta t_{e}}\int_{0}^{\Delta t_{e}}\frac{E_{i,k,e}\left(t\right)}{C_{i,r^{*}}^{\mathrm{eff}}}\;dt,$$

where $E_{i,k,e}\left(t\right)$ is the selected exposure metric and $C_{i,r^{*}}^{\mathrm{eff}}$ is the effect reference for the receptor or receptor group driving the assessment. The receptor index is explicit because a pulse can be acute for one receptor group and marginal for another. If a single receptor group drives the assessment, $r^{*}$ is reported as the dominant receptor or receptor-group reference. The activation multiplier may then be calibrated so that $\alpha_{i,k,e}$ increases with $P_{i,k,e,r^{*}}^{\exp}$, with an upper bound set by $\Lambda_{i}$. This formulation is important because short, high-intensity resuspension events may not be captured by annual or seasonal concentration averages, yet they may dominate acute pore-water exposure, particle ingestion, or early life-stage toxicity [40,43,44,66].

### 2.14. Mass-Conservation Ceiling for Event-Driven Exposure

An activation multiplier is physically meaningful only if the implied exposure remains compatible with the contaminant inventory that can be mobilized during the scenario. The CSTAP therefore includes a mass-conservation audit for erosive, dredging, storm, and reworking scenarios. Consider a surface control area $A_{k}^{\mathrm{surf}}$ and an event-accessible active thickness $L_{k,e}^{\mathrm{act}}$, where $0\leq L_{k,e}^{\mathrm{act}}\leq D_{k,e}^{\mathrm{er}}$ if a stratigraphic accessibility calculation is used. The mobilizable inventory for contaminant *i* is defined as

(60) $$M_{i,k,e}^{\mathrm{mob}}=A_{k}^{\mathrm{surf}}\rho_{b,k}L_{k,e}^{\mathrm{act}}f_{i,k,e}^{\mathrm{mob}}\left[\eta_{i,k,e}^{\mathrm{sol}}C_{i,k}^{\mathrm{sol}}+\eta_{k,e}^{\mathrm{pw}}\frac{\varepsilon_{k}}{\rho_{b,k}}C_{i,k}^{\mathrm{pw}}\right],$$

where $f_{i,k,e}^{\mathrm{mob}}$ is the fraction of the active layer effectively mobilized, $\eta_{i,k,e}^{\mathrm{sol}}$ is the fraction of the solid-associated inventory that can enter the exposure domain, and $\eta_{k,e}^{\mathrm{pw}}$ is the fraction of pore water released or exchanged. All three coefficients are bounded between 0 and 1 and must be declared. Equation (60) keeps the solid-associated and pore-water-equivalent inventories on the same dry-mass basis introduced in Equation (22).

If the mobilized mass is diluted into an event mixing volume

(61) $$V_{k,e}^{w,\mathrm{mix}}=A_{k}^{\mathrm{surf}}h_{k,e}^{\mathrm{mix}}\xi_{k,e}^{\mathrm{dil}},\;\xi_{k,e}^{\mathrm{dil}}\geq 1,$$

then the concentration ceiling for a water-column-equivalent exposure is

(62) $$C_{i,k,e}^{\mathrm{ceil}}=\frac{M_{i,k,e}^{\mathrm{mob}}}{V_{k,e}^{w,\mathrm{mix}}}.$$

The dilution factor $\xi_{k,e}^{\mathrm{dil}}$ accounts for exchange with adjacent water, plume spreading, or tidal flushing. In a closed laboratory elutriate, $\xi_{k,e}^{\mathrm{dil}}$ may approach unity; in an open coastal plume, it should exceed unity and may dominate the ceiling. The event exposure entering Equation (59) is therefore not allowed to exceed the available inventory at each time point:

(63) $$C_{i,k,e}^{\mathrm{evt}}\left(t\right)=\min\left[E_{i,k,e}^{\mathrm{raw}}\left(t\right),C_{i,k,e}^{\mathrm{ceil}}\right],$$

where $E_{i,k,e}^{\mathrm{raw}}\left(t\right)$ is the uncapped exposure predicted from resuspension, desorption, or pore-water exchange. For time-varying events, the cap should be applied to $E_{i,k,e}^{\mathrm{raw}}\left(t\right)$ before integration in Equation (59). When a scalar event-summary multiplier is required, define the event-mean raw exposure as

(64) $${\bar{E}}_{i,k,e}^{\mathrm{raw}}=\frac{1}{\Delta t_{e}}\int_{0}^{\Delta t_{e}}E_{i,k,e}^{\mathrm{raw}}\left(t\right)\;dt.$$

The activation multiplier can then be mass-corrected as

(65) $$\alpha_{i,k,e}^{\mathrm{mc}}=1+\left(\alpha_{i,k,e}-1\right)\min\left[1,\frac{C_{i,k,e}^{\mathrm{ceil}}}{{\bar{E}}_{i,k,e}^{\mathrm{raw}}+c_{i}^{\mathrm{evt},0}}\right],$$

where $c_{i}^{\mathrm{evt},0}$ is an event-exposure stabilizer with the same units as ${\bar{E}}_{i,k,e}^{\mathrm{raw}}$. It is distinct from the pore-water stabilizer $c_{i}^{0}$ used in Equation (36). Equation (65) guarantees $1\leq \alpha_{i,k,e}^{\mathrm{mc}}\leq \alpha_{i,k,e}$ when $\alpha_{i,k,e}\geq 1$. It also clarifies the physical role of event forcing: hydrodynamic or geochemical disturbance can accelerate release and change exposure pathways, but it cannot create more contaminant mass than the accessible sediment contains. This ceiling is especially important for theoretical protocols, because it prevents scenario multipliers from becoming unconstrained surrogates for concern and ties activation to cohesive-sediment transport, mixing, dilution, and inventory conservation [40,41,65,71,72,77]. The ceiling in Equations (62)–(65) is applied only to exposure metrics that can be expressed as concentration, mass per volume, or water-column-equivalent release. Particle number, particle surface area, ingestion rate, tissue-transfer proxies, and heterogeneous route metrics require a separate inventory-compatible ceiling and must not be capped with $C_{i,k,e}^{\mathrm{ceil}}$ unless a defensible unit conversion is specified.

### 2.15. Temporal Activation Memory and Post-Event Recovery

The finite-event exposure number in Equation (59) captures the magnitude of an exposure pulse, but it does not by itself distinguish a brief disturbance from persistent forcing, nor does it describe delayed recovery after dredging, hypoxia, reoxygenation, or storm-driven resuspension. The CSTAP therefore includes an optional activation-memory state, $Z_{i,k,e}^{\mathrm{act}}\left(t\right)$, for applications in which event duration, recurrence, and post-event monitoring are toxicologically relevant. The state variable is dimensionless, bounded between 0 and 1, and is defined by

(66) $$\frac{dZ_{i,k,e}^{\mathrm{act}}}{dt}=k_{i,k}^{\mathrm{act}}q_{i,k,e}^{\mathrm{evt}}\left(t\right)\left[1-Z_{i,k,e}^{\mathrm{act}}\left(t\right)\right]-k_{i,k}^{\mathrm{rec}}Z_{i,k,e}^{\mathrm{act}}\left(t\right),$$

where $k_{i,k}^{\mathrm{act}}$ is the activation-rate coefficient, $k_{i,k}^{\mathrm{rec}}$ is the recovery-rate coefficient, and $q_{i,k,e}^{\mathrm{evt}}\left(t\right)$ is a normalized contaminant–site–scenario event-forcing intensity. In temporal-memory applications, $q_{i,k,e}^{\mathrm{evt}}\left(t\right)$ should be assigned as a normalized form of $I_{i,k,e}^{\mathrm{act}}\left(t\right)$ or as a declared pathway-specific forcing that preserves the physical, chemical, biological-contact, and stratigraphic components entering Equation (41). One admissible form is $q_{i,k,e}^{\mathrm{evt}}\left(t\right)=I_{i,k,e}^{\mathrm{act}}\left(t\right)/\left[I_{i}^{\mathrm{ref}}+I_{i,k,e}^{\mathrm{act}}\left(t\right)\right]$, with $I_{i}^{\mathrm{ref}}>0$. If a scenario-level forcing common to all contaminants is used instead, it must be explicitly declared and contaminant-specific sensitivity must remain encoded in $I_{i,k,e}^{\mathrm{act}}$ and the activation-sensitivity coefficients. Equation (66) has the form of a bounded first-order relaxation process: activation memory grows only when event forcing is present and decays when forcing is removed. The nonlinear term $1-Z_{i,k,e}^{\mathrm{act}}$ prevents the memory variable from exceeding unity.

For a constant forcing level $q_{i,k,e}^{\mathrm{evt}}\left(t\right)=q_{i,e}$ over an event interval, the solution is

(67) $$Z_{i,k,e}^{\mathrm{act}}\left(t\right)=Z_{i,k,e}^{\mathrm{act},\infty }+\left[Z_{i,k,e}^{\mathrm{act}}\left(0\right)-Z_{i,k,e}^{\mathrm{act},\infty }\right]\exp\left[-\left(k_{i,k}^{\mathrm{act}}q_{i,e}+k_{i,k}^{\mathrm{rec}}\right)t\right],$$

with steady state

(68) $$Z_{i,k,e}^{\mathrm{act},\infty }=\frac{k_{i,k}^{\mathrm{act}}q_{i,e}}{k_{i,k}^{\mathrm{act}}q_{i,e}+k_{i,k}^{\mathrm{rec}}}.$$

After forcing ceases at time $t_{e}$, recovery follows

(69) $$Z_{i,k,e}^{\mathrm{act}}\left(t\right)=Z_{i,k,e}^{\mathrm{act}}\left(t_{e}\right)\exp\left[-k_{i,k}^{\mathrm{rec}}\left(t-t_{e}\right)\right],\;t>t_{e},$$

and the recovery half-time is

(70) $$t_{\mathrm{rec},i,k}^{1/2}=\frac{\ln2}{k_{i,k}^{\mathrm{rec}}}.$$

Thus, two events with the same maximum shear stress or redox departure can produce different CSTAI responses if one is too brief for activation to develop or if post-event recovery is slow.

When the memory layer is used, the activation multiplier entering CSTAI is replaced by a time-averaged multiplier,

(71) $$\alpha_{i,k,e}^{\mathrm{mem}}=\frac{1}{\Delta t_{e}}\int_{0}^{\Delta t_{e}}\min\left\{1+\Lambda_{i},\max\left[1,1+\Lambda_{i}Z_{i,k,e}^{\mathrm{act}}\left(t\right)\right]\right\}\;dt.$$

Equation (71) averages the bounded activation-memory state defined in Equation (66), thereby incorporating both activation and recovery through $k_{i,k}^{\mathrm{act}}$ and $k_{i,k}^{\mathrm{rec}}$. Because $0\leq Z_{i,k,e}^{\mathrm{act}}\left(t\right)\leq 1$, the memory multiplier remains bounded between 1 and $1+\Lambda_{i}$. The monitoring implication is explicit: the predicted activation class depends on forcing duration, sampling time, recovery time, and physical, chemical, biological-contact, and stratigraphic activation intensities, not only on the peak value of a mechanical disturbance descriptor. If temporal data are unavailable, the CSTAP uses the static multiplier in Equation (43) and reports temporal resolution as an uncertainty component.

### 2.16. Stratigraphic Accessibility of Contaminated Layers

Coastal sediment assessments often rely on surface samples, whereas ports, lagoons, and industrial bays may contain strongly stratified contamination inherited from historical inputs. A buried contaminated interval is not automatically bioavailable, but it can become relevant when erosion, dredging, bioturbation, anchor scouring, storm waves, or remediation works connect it to the biologically active layer. The CSTAP therefore treats vertical position as an accessibility problem rather than as a purely descriptive core-log attribute.

Let $l=1,\ldots ,N_{k}^{z}$ denote the sediment-depth interval in core or site *k*, with midpoint $z_{k,l}^{\mathrm{mid}}$ measured downward from the sediment–water interface. Let $D_{k,e}^{\mathrm{er}}$ be the event-accessible depth under scenario *e*, defined as the larger of the expected erosion depth, dredging contact depth, bioturbation depth, or engineered disturbance depth relevant to the assessment question. A smooth depth-accessibility weight is defined as

(72) $$\lambda_{k,l,e}^{z}={\left[1+\exp\left(\frac{z_{k,l}^{\mathrm{mid}}-D_{k,e}^{\mathrm{er}}}{z_{k}^{0}}\right)\right]}^{-1},$$

where $z_{k}^{0}>0$ is a transition length that expresses uncertainty in the effective activation depth. Equation (72) approaches unity when the layer lies within the event-accessible zone and approaches zero when the layer is well below the physically or biologically accessible depth. The logistic form avoids an artificial discontinuity at $D_{k,e}^{\mathrm{er}}$ while retaining a transparent interpretation.

For stratified cores, the layer-specific raw activation contribution $\Theta_{k,l,e}$ is first computed using the same contaminant, bioavailability, mixture, activation, receptor, and evidence-reporting modules used for surface sediment. The site–scenario score is then aggregated as

(73) $$\Theta_{k,e}^{z}=\frac{\sum_{l=1}^{N_{k}^{z}}\lambda_{k,l,e}^{z}\;m_{k,l}\;\Theta_{k,l,e}}{\sum_{l=1}^{N_{k}^{z}}\lambda_{k,l,e}^{z}\;m_{k,l}+\varepsilon_{z}},$$

where $m_{k,l}$ is the dry sediment mass or thickness-normalized mass represented by interval *l*, and $\varepsilon_{z}$ is a small mass-scale regularizer with the same units as $m_{k,l}$, preventing division by zero in purely surface-only datasets. If dimensionless layer weights are used instead of masses, the masses should first be normalized, and $\varepsilon_{z}$ should be replaced by a dimensionless stabilizer. Equation (73) makes explicit that two cores with identical maximum concentrations may have different activation potential if one contains contaminants within the event-accessible layer while the other stores them below the probable disturbance depth. This is particularly relevant for dredging, sediment-capping design, post-remediation monitoring, and reconstruction of contamination thickness in industrialized coastal basins.

The dimensionless stratigraphic accessibility factor reported by the CSTAP is

(74) $$A_{k,e}^{z}=\frac{\Theta_{k,e}^{z}}{\max_{l}\left(\Theta_{k,l,e}\right)+\varepsilon_{\Theta }},$$

with $0\leq A_{k,e}^{z}\leq 1$ when all layer scores are non-negative. Values near unity indicate that high-contamination layers are shallow or event-accessible; values near zero indicate deep storage with low short-term activation probability under the specified event. The factor is not a substitute for core dating, sediment geotechnics, or site-specific dredging design, but it prevents the CSTAP from treating a deeply buried legacy layer as equivalent to a surface deposit unless a credible event pathway connects that layer to exposure. In stratified-core applications, $\Theta_{k,e}^{z}$ is the preferred final raw score because it aggregates accessible intervals directly. $A_{k,e}^{z}$ is a diagnostic accessibility factor and should not be multiplied into $\Theta_{k,e}^{z}$ again unless an explicitly reduced stress-test formulation is declared. When Equation (41) is used with stratigraphic information, the optional stratigraphic component may be set as $I_{k,e}^{z}=A_{k,e}^{z}$ or as another declared, dimensionless accessibility proxy.

### 2.17. Exposure-Route Decomposition Before Receptor Weighting

The receptor module is more defensible when sensitivity is not treated as a single scalar detached from the pathway by which exposure occurs. The CSTAP therefore includes a route-resolved layer before ecological weighting. The exposure-route set is denoted by

(75) $$G^{\exp}=\left\{\mathrm{pw},\mathrm{ing},\mathrm{pc},\mathrm{tr}\right\},$$

where pw denotes dissolved or pore-water uptake, ing denotes sediment ingestion, pc denotes particle/contact exposure, and tr denotes trophic or food-web transfer. The set can be expanded for site-specific applications, but each route must have a stated measurement surrogate or modelling proxy.

For each receptor *r*, route weights form a normalized simplex,

(76) $$0\leq \nu_{g,r}^{\mathrm{rt}}\leq 1,\;\sum_{g\in G^{\exp}}\nu_{g,r}^{\mathrm{rt}}=1.$$

Thus, a deposit-feeding amphipod may receive large weights for pore-water uptake and sediment ingestion, a suspension-feeding bivalve may emphasize dissolved and particle-associated exposure, and a demersal fish may require a larger trophic-transfer component. Route weights are ecological descriptors, not toxicological effects; they should therefore be assigned from receptor biology, site use, or the protection goal before the CSTAI score is calculated.

Each route is converted to a non-negative, dimensionless accessibility factor. Let $E_{i,k,e,g}^{\mathrm{rt}}$ be the exposure metric for contaminant *i* at site *k* under scenario *e* along route *g*. It may be a pore-water concentration, freely dissolved concentration, chemical activity, DGT-labile flux, ingestible sediment burden, microplastic particle number, or tissue-transfer proxy, provided that the corresponding reference value $E_{i,g}^{\mathrm{rt},\mathrm{ref}}$ has compatible units. The default bounded normalization is

(77) $$A_{i,k,e,g}^{\mathrm{rt}}=\frac{E_{i,k,e,g}^{\mathrm{rt}}}{E_{i,g}^{\mathrm{rt},\mathrm{ref}}+E_{i,k,e,g}^{\mathrm{rt}}},\;E_{i,k,e,g}^{\mathrm{rt}}\geq 0,\;E_{i,g}^{\mathrm{rt},\mathrm{ref}}>0.$$

Equation (77) is deliberately saturating: it prevents a single route proxy with uncertain high values from dominating the receptor layer without bound. In this form, $E_{i,g}^{\mathrm{rt},\mathrm{ref}}$ is interpreted as a route-accessibility half-saturation constant rather than necessarily as a toxicological effect threshold; when $E_{i,k,e,g}^{\mathrm{rt}}=E_{i,g}^{\mathrm{rt},\mathrm{ref}}$, the route factor is 0.5 by construction. If a toxicological benchmark is used instead, an alternative threshold-based normalization such as $\min(1,E_{i,k,e,g}^{\mathrm{rt}}/E_{i,g}^{\mathrm{rt},\mathrm{ref}})$ must be declared. When a route-specific benchmark is unavailable, the CSTAP allows a categorical value of $A_{i,k,e,g}^{\mathrm{rt}}$ only if the data tier and uncertainty flag are reported.

The route-resolved receptor term is then

(78) $$V_{i,k,e}^{\mathrm{rt}}=\sum_{r=1}^{N_{r}}\lambda_{r,k}^{\mathrm{rec}}\zeta_{i,r}\left(\sum_{g\in G^{\exp}}\nu_{g,r}^{\mathrm{rt}}\;\kappa_{i,r,g}^{\mathrm{rt}}\;A_{i,k,e,g}^{\mathrm{rt}}\right),$$

where $\kappa_{i,r,g}^{\mathrm{rt}}$ is a route-specific relative potency coefficient. The default value is $\kappa_{i,r,g}^{\mathrm{rt}}=1$ unless receptor- and route-specific evidence supports another value. The unrouted receptor term in Equation (79) is recovered when the inner route sum equals unity. Consequently, route decomposition enriches the CSTAP without changing its limiting behavior: users with no route-resolved information may use $V_{i,k}^{\mathrm{rec}}$, whereas users with pore-water, ingestion, particle, or trophic evidence can calculate $V_{i,k,e}^{\mathrm{rt}}$.

Default route examples are provided in Supplementary Table S5 to keep the main protocol focused on equations and reporting outputs.

### 2.18. Receptor Vulnerability and Ecological Weighting

The receptor component links exposure to ecological relevance. For backward-compatible screening when route data are unavailable, the CSTAP defines the unrouted receptor-weighted vulnerability term as

(79) $$V_{i,k}^{\mathrm{rec}}=\sum_{r=1}^{N_{r}}\lambda_{r,k}^{\mathrm{rec}}\zeta_{i,r},\;\sum_{r=1}^{N_{r}}\lambda_{r,k}^{\mathrm{rec}}=1,\;\lambda_{r,k}^{\mathrm{rec}}\geq 0.$$

The coefficient $\zeta_{i,r}$ reflects the sensitivity of receptor *r* to contaminant *i*, while $\lambda_{r,k}^{\mathrm{rec}}$ represents the receptor’s ecological or management relevance at site *k*. For example, a fine-grained lagoon sediment with abundant deposit-feeding amphipods and polychaetes may assign higher weights to benthic invertebrates; a shellfish area may assign higher relevance to bivalves and trophic transfer; a nursery habitat may include demersal fish and larval stages. This construction avoids treating all receptors as equally relevant across all coastal settings and allows the CSTAP to be aligned with site-specific protection goals. In route-resolved applications, Equation (78) replaces Equation (79) in the final score; in Tier 0 applications, $V_{i,k}^{\mathrm{rec}}$ remains an explicit fallback rather than an implicit assumption [6,7,78].

### 2.19. Uncertainty-Priority and Evidence Separation

The CSTAP separates the estimated activation magnitude from the evidential strength supporting that estimate. The uncertainty-priority coefficient $U_{k}$ prevents a sparse dataset from being treated as equivalent to a well-characterized site, but it is not used to inflate the primary CSTAI activation score. It is defined as

(80) $$U_{k}=1+u_{k}^{\mathrm{chem}}+u_{k}^{\mathrm{bioav}}+u_{k}^{\mathrm{mix}}+u_{k}^{\mathrm{event}}+u_{k}^{\mathrm{eco}},$$

where each *u* term ranges from 0 to a prescribed maximum and represents missing or low-quality information on chemical coverage, bioavailability, mixture interactions, disturbance scenarios, and ecological receptors. For management screening, an optional priority score can be reported as

(81) $$\Pi_{k,e}^{\mathrm{prio}}=U_{k}\Theta_{k,e}.$$

Equation (81) is not interpreted as toxicity. It is a conservative triage output indicating that a site–scenario pair with poor evidence may deserve additional measurement before a management decision is made. The activation class is therefore reported together with $U_{k}$ and the evidence-adequacy coefficient $K_{k,e}^{\mathrm{evi}}$ rather than being silently inflated by missing information.

For reporting, the CSTAP outputs should be read on four separate tracks: $\Psi_{k,e}$ describes toxicological activation potential; $K_{k,e}^{\mathrm{evi}}$ describes evidential support; $U_{k}$ describes the priority created by missing or weak information; and, where contested interpretation is relevant, ${\mathrm{FIG}}_{k}$ describes procedural integrity. Only $\Psi_{k,e}$ determines the exploratory CSTAI class. The other outputs qualify confidence, triage priority, or evidentiary defensibility; they do not constitute measured toxicity and they do not by themselves shift the primary activation class.

When several activation modules are available, the CSTAP first computes a pre-mass-correction activation multiplier $\alpha_{i,k,e}^{\mathrm{pre}}$ from the declared static, temperature–oxygen, or temporal-memory layer. If the mass-conservation audit is triggered, the admitted multiplier is

$$\alpha_{i,k,e}^{†}=1+\left(\alpha_{i,k,e}^{\mathrm{pre}}-1\right)f_{i,k,e}^{\mathrm{mc}};$$

otherwise, $\alpha_{i,k,e}^{†}=\alpha_{i,k,e}^{\mathrm{pre}}$. This sequence ensures that the mass ceiling is applied to the selected activation pathway rather than only to the static disturbance form.

### 2.20. Definition of CSTAI

The contaminant-specific CSTAP contribution for site *k* under scenario *e* is

(82) $$\Omega_{i,k,e}=H_{i,k}\;\beta_{i,k}^{\mathrm{eff}}\;\mu_{i,k}\;\alpha_{i,k,e}^{†}\;V_{i,k,e}^{\mathrm{rt}},$$

where $\alpha_{i,k,e}^{†}$ denotes the activation multiplier actually admitted into the final score, and $V_{i,k,e}^{\mathrm{rt}}$ is the route-resolved receptor term from Equation (78). When route information is unavailable, $V_{i,k,e}^{\mathrm{rt}}=V_{i,k}^{\mathrm{rec}}$ by explicit fallback. In static applications, $\alpha_{i,k,e}^{†}=\alpha_{i,k,e}$; in temperature–oxygen applications, it may be replaced by $\alpha_{i,k,e}^{\mathrm{TO}}$; in temporal-memory applications it may be replaced by $\alpha_{i,k,e}^{\mathrm{mem}}$; and in event scenarios subject to the inventory audit, it is replaced by $\alpha_{i,k,e}^{\mathrm{mc}}$ from Equation (65). This notation separates the process-specific activation calculation from the mass-conserving multiplier ultimately used in the CSTAI. The raw Coastal Sediment Toxicological Activation Index is then

(83) $$\Theta_{k,e}=\sum_{i=1}^{N_{c}}\Omega_{i,k,e}.$$

Because $\Theta_{k,e}$ may span orders of magnitude, the CSTAP reports the log-transformed score

(84) $$\Psi_{k,e}=\log_{10}\left(1+\Theta_{k,e}\right).$$

The transformation preserves monotonicity while reducing scale dominance by a single extreme contaminant. The score can be computed for baseline conditions and for specific event scenarios, producing a site–scenario matrix $\Psi_{k,e}$.

A useful mathematical property follows directly. *Proposition.* If $H_{i,k}$, $\beta_{i,k}^{\mathrm{eff}}$, $\mu_{i,k}$, $\alpha_{i,k,e}^{†}$, and $V_{i,k,e}^{\mathrm{rt}}$ are non-negative, then $\Psi_{k,e}$ is monotonically non-decreasing in each component of the product in Equation (83) when the remaining components are held fixed. *Proof.* The partial derivative of $\Theta_{k,e}$ with respect to any non-negative fixed component is the product of the remaining non-negative components and is therefore non-negative. Since $d\log_{10}\left(1+x\right)/dx={\left[\left(1+x\right)\ln10\right]}^{-1}>0$ for x≥0, the monotonicity of $\Psi_{k,e}$ follows under the stated condition. This property is local and conditional: if antagonistic mixture coefficients are admitted through $\gamma_{ij}<0$, the mixture multiplier $\mu_{i,k}$ may decrease as another contaminant changes, and global monotonicity is not asserted. Therefore, monotonicity claims apply either with $\mu_{i,k}$ fixed, with $\gamma_{ij}\geq 0$, or with no antagonistic interaction admitted.

For protocol interrogation, the CSTAP also reports local elasticities. If *x* is any positive multiplicative component in Equation (83), the dimensionless elasticity of the log-score is

(85) $$L_{k,e}^{x}=\frac{\partial \Psi_{k,e}}{\partial \ln x}=\frac{x}{(1+\Theta_{k,e})\ln10}\frac{\partial \Theta_{k,e}}{\partial x}.$$

This derivative identifies whether uncertainty reduction should prioritize chemistry, bioavailability, mixture interaction, hydrodynamic activation, or receptor sensitivity. A site dominated by $L^{\beta }$ requires better pore-water, passive-sampler, DGT, or AVS–SEM data; a site dominated by $L^{\alpha }$ requires better event physics, critical shear stress, dredging, hypoxia, or storm-scenario characterization. Thus, the mathematics directly informs monitoring design rather than merely producing a final class.

### 2.21. Uncertainty Propagation and Sensitivity Decomposition

The uncertainty-priority coefficient in Equation (80) is a transparent screening device, while stochastic uncertainty can be propagated mathematically when probability distributions are available. Using the raw score defined in Equation (83), the contribution share of contaminant *i* is

(86) $$q_{i,k,e}^{\mathrm{con}}=\left\{\begin{array}{cc} \frac{\Omega_{i,k,e}}{\Theta_{k,e}}, & \Theta_{k,e}>0,\\ 0, & \Theta_{k,e}=0, \end{array}\right.\;0\leq q_{i,k,e}^{\mathrm{con}}\leq 1.$$

For $\Theta_{k,e}>0$, the contribution shares sum to unity. For $\Theta_{k,e}=0$, all shares are set to zero by convention because no contaminant contributes to a positive activation-potential score. For $\Theta_{k,e}>0$ and any positive multiplicative factor $x_{i,k,e}$ inside $\Omega_{i,k,e}$, the local derivative of $\ln\Theta_{k,e}$ with respect to $\ln x_{i,k,e}$ is $q_{i,k,e}^{\mathrm{con}}$. This result gives an exact first-order decomposition of sensitivity across contaminants: uncertainty reduction should first target the contaminants with the largest $q_{i,k,e}^{\mathrm{con}}$ and the process terms with the largest elasticities.

If the logarithms of uncertain protocol inputs are collected in a vector $y_{k,e}$ with covariance matrix $\sum_{k,e}$, the delta-method approximation for the variance of the raw-score logarithm is

(87) $$\mathrm{Var}\;\left[\ln\Theta_{k,e}\right]\approx \nabla_{y}\ln\Theta_{k,e}^{T}\;\sum_{k,e}\;\nabla_{y}\ln\Theta_{k,e}.$$

The same approximation can be transferred to the reported log-score by

(88) $$\mathrm{Var}\;\left[\Psi_{k,e}\right]\approx {\left[\frac{\Theta_{k,e}}{(1+\Theta_{k,e})\ln10}\right]}^{2}\mathrm{Var}\;\left[\ln\Theta_{k,e}\right].$$

Equations (86)–(88) allow the CSTAP to report not only a class but also the dominant uncertainty sources qualifying the robustness of that class. This is essential for a protocol intended to guide monitoring, because the most valuable next measurement is not necessarily the easiest measurement; it is the measurement that most reduces uncertainty in $\Psi_{k,e}$ and proximity to the exploratory class thresholds [6,7,79].

### 2.22. Class-Assignment Uncertainty and Calibration Target

The deterministic class defined below is sufficient for a first protocol implementation, but data-rich applications should report the uncertainty distribution over the exploratory class assignment. Let $b_{0}=-\infty$, $b_{1}=0.30$, $b_{2}=0.70$, $b_{3}=1.10$, $b_{4}=1.50$, and $b_{5}=+\infty$ denote the class boundaries for Classes I–V. If the uncertainty analysis gives an expected score $m_{k,e}^{\Psi }$ and a score variance $v_{k,e}^{\Psi }$, a first-order normal approximation gives

(89) $$\mathrm{Pr}\left({\mathrm{Class}}_{k,e}=m\right)=\Phi \;\left(\frac{b_{m}-m_{k,e}^{\Psi }}{\sqrt{v_{k,e}^{\Psi }+\epsilon_{\Psi }}}\right)-\Phi \;\left(\frac{b_{m-1}-m_{k,e}^{\Psi }}{\sqrt{v_{k,e}^{\Psi }+\epsilon_{\Psi }}}\right),$$

where m=1,…,5, Φ is the standard normal cumulative distribution function, and $\epsilon_{\Psi }>0$ is a small numerical stabilizer. The uncertainty mass above the elevated-activation boundary can then be reported as

(90) $$\mathrm{Pr}\left({\mathrm{Class}}_{k,e}\geq \mathrm{IV}\right)=\mathrm{Pr}\left({\mathrm{Class}}_{k,e}=4\right)+\mathrm{Pr}\left({\mathrm{Class}}_{k,e}=5\right).$$

This uncertainty distribution is useful as a boundary-sensitivity summary when $\Psi_{k,e}$ is close to a class threshold or when the input data are sparse. It avoids overinterpreting artificial distinctions between, for example, a score of 1.09 and a score of 1.11 when uncertainty is large, but it does not redefine the deterministic class assignment. Until calibrated against observed toxicity outcomes, however, it is a provisional uncertainty summary rather than a validated class-assignment model.

For future calibration, the CSTAP can be fitted to observed toxicity labels without changing the protocol structure. Let $y_{k,e}=1$ when an adverse effect is observed under site–scenario pair (k,e). The primary calibration layer is the logistic likelihood defined in Section 2.32. The expression below is its regularized extension for weakly identified interaction and activation parameters:

(91) $$O^{\mathrm{cal}}\left(\vartheta ,\varrho_{0},\varrho_{1}\right)=-\sum_{k,e}\left[y_{k,e}\ln P_{k,e}^{\mathrm{tox}}+\left(1-y_{k,e}\right)\ln\left(1-P_{k,e}^{\mathrm{tox}}\right)\right]+\lambda_{\Gamma }\sum_{i<j}\left|\gamma_{ij}\right|+\lambda_{\alpha }\sum_{i}{\left(\Lambda_{i}-\Lambda_{i}^{0}\right)}^{2},$$

where $P_{k,e}^{\mathrm{tox}}$ is the logistic probability defined in Equation (99), ϑ is the parameter vector, $\lambda_{\Gamma }$ penalizes unsupported mixture interactions, $\lambda_{\alpha }$ penalizes excessive activation amplitudes, and $\Lambda_{i}^{0}$ is a prior activation amplitude. Equation (91) is not a second calibration model; it is the regularized form of the likelihood-based calibration in Equation (99), useful when weakly identified parameters such as $\gamma_{ij}$, $\Lambda_{i}$, or activation sensitivities must be constrained. It is not required for first use of CSTAP, but it gives the protocol a formal route from theoretical scoring to calibrated prediction using paired chemistry–toxicity or pre-/post-event datasets [7,8,10,11,79].

A Bayesian extension can be used when evidence is heterogeneous across ports, lagoons, and estuaries. If D denotes the available paired dataset and p(ϑ) denotes a prior constrained to physically admissible parameter values, then

(92) p(ϑ∣D)∝p(D∣ϑ)p(ϑ).

The Bayesian form is not a new index; it is a future calibration envelope for CSTAP parameters and thresholds. Until paired chemistry–toxicity datasets are used, class-assignment uncertainty summaries are provisional rather than validated probability forecasts of effects [7,8,79,80].

### 2.23. Boundary Conditions and Mathematical Sanity Checks

The CSTAP was constructed to satisfy explicit boundary conditions. If all hazard ratios are zero, then $\Omega_{i,k,e}=0$, $\Theta_{k,e}=0$, and $\Psi_{k,e}=0$, irrespective of event forcing. If a sediment is contaminated but physically and chemically stable, $\alpha_{i,k,e}$ tends toward its baseline value and the class is controlled by chemical burden, bioavailability, mixture pressure, and receptor vulnerability. If disturbance intensity increases while other terms remain non-negative, the monotonicity property ensures that the activation class cannot decrease unless a documented antagonistic mixture term is introduced. Finally, if data are sparse, $U_{k}$ increases priority for further assessment only through the separate priority output $\Pi_{k,e}^{\mathrm{prio}}$, thereby avoiding confusion between ignorance and measured toxicity. These sanity checks are necessary for regulatory and scientific defensibility: a protocol that violates them would be numerically convenient but physically misleading.

### 2.24. Scoring Rules and Exploratory Toxicological-Activation-Potential Classes

The CSTAP supports three nested implementation levels. The core level may use categorical terms when data are incomplete; the extended level uses measured or independently constrained process functions; exploratory optional layers are reported separately when their evidential basis is incomplete or purpose-specific. Categorical core applications assign each module a score from 0 to 4, where 0 is negligible or absent and 4 is very high. Continuous outputs from Equations (24)–(84) can be converted to exploratory classes using threshold values. The class thresholds defined below are provisional defaults for protocol testing only. They are not proposed as universal effect thresholds, regulatory limits, or validated management classes, and they should be recalibrated when sufficient paired chemistry–exposure–toxicity datasets are available.

Before any CSTAI calculation, the analytical panel must be declared. Adding marginal analytes after inspecting the score must be reported as a panel-sensitivity analysis rather than as a primary class shift. Contributions below a declared de minimis threshold may be reported for transparency, but they should not be used to infer an event-conditioned class shift without panel-size sensitivity analysis.

When categorical 0–4 inputs are used in a core screening application, the mapping to multipliers must be declared. Table 9 provides a bounded ordinal mapping intended to improve comparability between data-limited applications. The entries are protocol-control values, not empirically calibrated toxicity coefficients or biological effect thresholds. The scale is anchored to a neutral multiplier of 1.00 and capped at 2.00 so that one proxy-based module cannot more than double an otherwise unchanged contaminant contribution in the default Core implementation. Categories 2 and 3 represent quarter- and half-scale amplification above the neutral baseline (1.25 and 1.50), whereas category 4 reaches the full default cap (2.00). This deliberately bounded progression preserves ordinal separation without implying a linear biological dose–response. A score of 0 must not simultaneously mean absence of contaminant, absence of evidence, and absence of activation; these cases are reported separately through $H_{i,k}$, $K_{k,e}^{\mathrm{evi}}$, and the relevant multiplier.

The resulting exploratory class thresholds are summarized in Table 10.

Mapping robustness must be evaluated when categorical defaults materially influence the score. At minimum, the default mapping should be compared with the compressed and linear-to-cap alternatives in Supplementary Table S13 when (i) a categorical module contributes at least 25% of $\Theta_{k,e}$, (ii) $\Psi_{k,e}$ lies within 0.10 of a class boundary, or (iii) a plausible alternative mapping changes the assigned class. A class change must be reported as *mapping-sensitive* and must not be interpreted beyond screening and prioritization. Measured or endpoint-calibrated process functions supersede all categorical defaults.

Because class boundaries are defined on $\Psi_{k,e}=\log_{10}\left(1+\Theta_{k,e}\right)$, Table 11 reports the equivalent raw-score thresholds. These conversions make the log scale transparent; the thresholds remain provisional protocol-testing defaults until calibrated against endpoint-specific datasets.

### 2.25. Protocol Workflow

**Figure 2:**
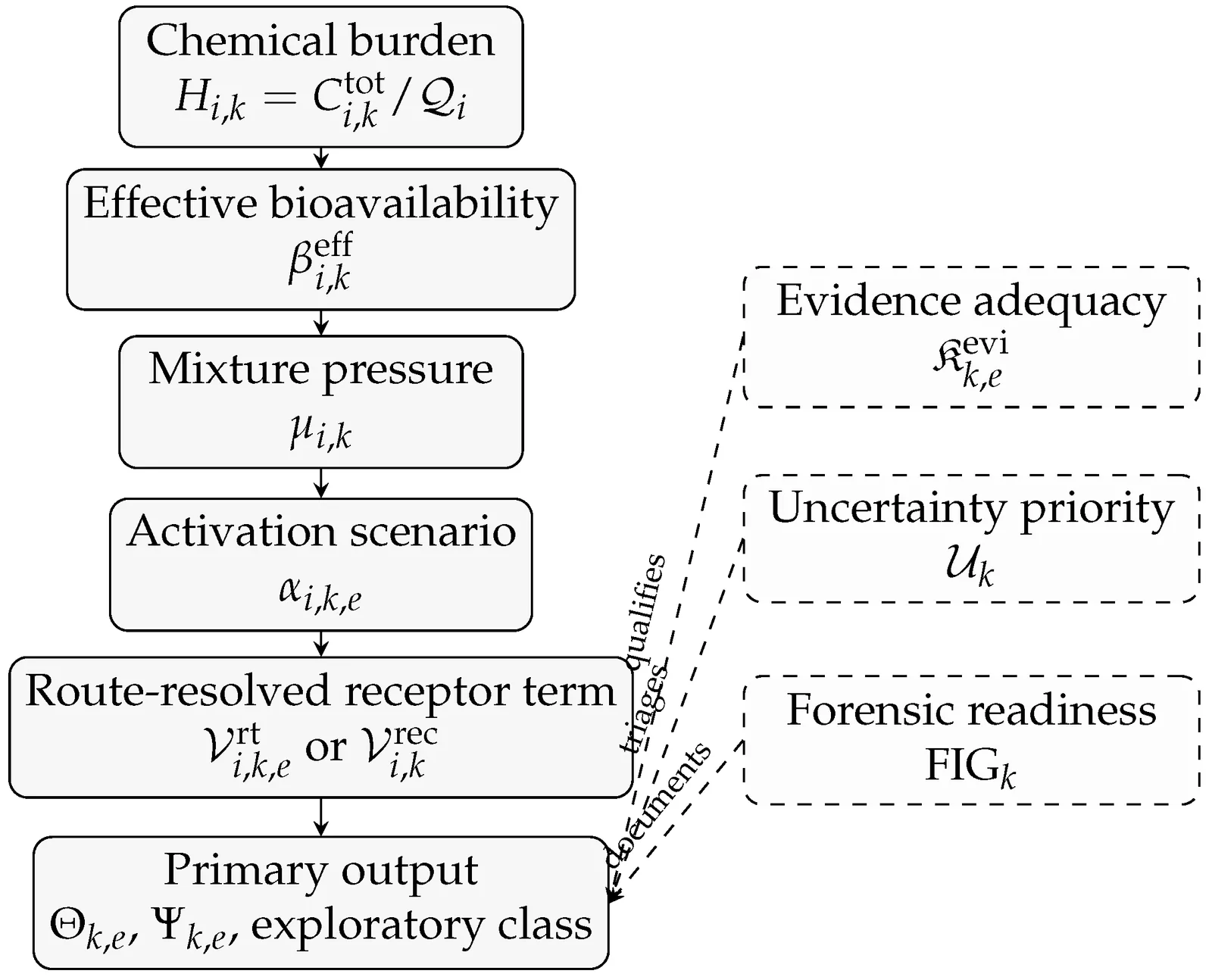
Coastal Sediment Toxicological Activation Protocol (CSTAP) workflow. Solid arrows define the primary scoring track that produces $\Theta_{k,e}$, $\Psi_{k,e}$, and the exploratory activation class. Dashed arrows are secondary reporting tracks: evidence adequacy, uncertainty priority, and forensic readiness qualify interpretation but do not alter the primary class.

The operational workflow is shown in Figure 2. Step 1 defines the spatial unit of analysis, such as a core interval, management cell, port basin, or sediment layer. Step 2 compiles chemical data and selects $Q_{i}$ values from relevant SQGs, background concentrations, regulatory thresholds, or site-specific benchmarks. Step 3 evaluates bioavailability using measured pore water, passive samplers, DGT, AVS–SEM, organic carbon, black carbon, sediment texture, pH, redox potential, and salinity. Step 4 evaluates mixture pressure through toxic units, co-occurrence, or interaction coefficients. Step 5 defines baseline and disturbance scenarios, including temperature, oxygenation, redox, and event-duration descriptors where relevant. Step 6 calculates the route-resolved receptor term $V_{i,k,e}^{\mathrm{rt}}$ or its explicit fallback $V_{i,k}^{\mathrm{rec}}$. Step 7 calculates $\Theta_{k,e}$ and $\Psi_{k,e}$. Step 8 assigns the exploratory CSTAI class from $\Psi_{k,e}$ and reports evidence adequacy, uncertainty priority, and forensic readiness separately. Step 9 links the four-track report to indicative follow-up measurements, such as additional monitoring, ecotoxicological testing, dredging-scenario review, remediation feasibility assessment, or post-event surveillance; these outputs remain illustrative until thresholds are calibrated and accepted for the site context.

### 2.26. Algorithmic Implementation and Reproducibility

The protocol can be implemented deterministically from the ordered operations in Table 12. This algorithmic statement documents the core arithmetic and input–output logic of the protocol. Independent users should obtain the same exploratory CSTAI class if they use the same input data, benchmarks, coefficients, and scenario definitions. A minimal reference implementation using Python 3 standard syntax, comma-separated-value (CSV) input tables, and no proprietary dependencies is provided as Supplementary Material together with a parameter dictionary, a worked example, and a checksum manifest. The code reproduces the worked example and does not yet implement every optional extended module. The manifest and machine-readable files follow the reproducibility logic of Findable, Accessible, Interoperable, and Reusable (FAIR)-aligned research objects by making inputs, outputs, scripts, and file identity explicit [81].

### 2.27. Core, Extended, and Exploratory Implementations

To reduce overparameterization and make the protocol usable in data-limited coastal settings, the CSTAP is specified in three nested implementation levels. The *core implementation* contains only the minimum scoring structure needed to express chemical burden, effective bioavailability, event-dependent activation, and receptor vulnerability. The *extended implementation* activates measured or independently constrained exposure, mixture, transport, stratigraphic, and receptor-route modules. The *exploratory layer* contains optional procedures, such as temporal memory, advanced interaction matrices, decision-theoretic translation, and evidentiary-integrity reporting, that should be used only when the required data and purpose are explicit.

In the core mode,

(93) $$\Theta_{k,e}^{\mathrm{core}}=\sum_{i=1}^{N_{c}}H_{i,k}\;\beta_{i,k}^{\mathrm{eff},\mathrm{core}}\;\alpha_{i,k,e}^{\mathrm{core}}\;V_{i,k}^{\mathrm{rec},\mathrm{core}},$$

with $\mu_{i,k}=1$ unless mixture evidence is available, with $\beta_{i,k}^{\mathrm{eff},\mathrm{core}}$ constrained by the information-source safeguard, and with $K_{k,e}^{\mathrm{evi}}$ capped by the declared data tier. Core-mode scores are burden-informed hypotheses for monitoring design; they are not quantitative predictions of toxicity. Extended-mode scores use Equation (83) and should be reported only when the decisive coefficients are measured, independently constrained, or justified by a declared site-specific rationale. Exploratory modules must be labelled as exploratory in the output and must not be allowed to imply regulatory readiness.

The required inputs and permitted interpretation at each implementation level are summarized in Table 13.

All applications must state whether the reported class is generated by the core, extended, or exploratory level. Default coefficients used in the core implementation are structured screening values rather than calibrated constants and must be replaced by site-specific measurements or calibration whenever the output is used beyond prioritization.

### 2.28. Quality-Control Gates

An exploratory CSTAI class is only interpretable if the calculation passes a minimum set of quality-control gates. These gates do not add new toxicological evidence; they determine whether the available evidence is internally consistent enough to support a class assignment. Failed gates should be reported as limitations and should either prevent numerical classification or downgrade the application to a lower data tier. Table 14 defines the gates used in the CSTAP.

The gates are deliberately conservative. They are designed to prevent three common errors: combining variables with incompatible units, treating missing exposure information as evidence of low concern, and allowing a disturbance label to change the score without a physical or biogeochemical mechanism. In this sense, the gates are part of the protocol’s mathematical hygiene rather than an additional subjective weighting system.

### 2.29. Forensic-Readiness Reporting Layer: Custody, Repeatability, and Destructive-Analysis Control

The CSTAP can be applied at three evidentiary levels: scientific, regulatory, and contested-use. The scientific level is sufficient for hypothesis generation, research comparison, and exploratory monitoring; detailed forensic requirements are optional and should be moved to supplementary checklists when they are not central to the ecotoxicological question. The regulatory level requires greater standardization of sampling plans, analytical quality control, documentation, and traceability. The contested-use level is reserved for contested environmental applications in which the result may be challenged, repeated, independently re-analyzed, or used to support source attribution, damage assessment, remediation liability, dredging disputes, or enforcement. The forensic-readiness reporting layer does not modify $H_{i,k}$, $\beta_{i,k}$, $\beta_{i,k}^{\mathrm{eff}}$, $\mu_{i,k}$, $\alpha_{i,k,e}$, $V_{i,k,e}^{\mathrm{rt}}$, $\Theta_{k,e}$, or $\Psi_{k,e}$. It determines whether the numerical CSTAP/CSTAI output is supported by samples and records that are sufficiently traceable, preserved, and repeatable to withstand independent scrutiny.

Contested-use applications should distinguish at least four physical sample roles. The *analytical aliquot* is consumed or altered by routine analyses. The *split duplicate* is produced from the same homogenized or stratigraphically matched material and quantifies subsampling and analytical variability. The *field duplicate* is independently collected from the same sampling unit and quantifies small-scale spatial and sampling variability. The *reserve aliquot* is sealed, documented, and preserved for counter-analysis. Where sediment cores are used, the same logic should be expressed as an analytical half-core, an archive half-core, and, for high-consequence sites, a dispute-reserve core or interval-specific reserve material. Composite samples may be useful for scientific screening, but contested-use CSTAP should preserve discrete georeferenced samples before any compositing step, because compositing can dilute localized signals and weaken source or depth attribution.

The order of operations is also part of evidentiary integrity. Non-destructive or minimally destructive documentation should precede destructive analysis whenever repeatability is required. This includes field and laboratory photography, unique sample identifiers, position and depth records, core logging, visible stratigraphy, wet mass, container type, seal status, and, where available, non-destructive scanning or imaging. Destructive measurements such as extraction, digestion, solvent treatment, elutriate preparation, pore-water separation, microplastic digestion, or bioassay preparation should be conducted only after the reserve and duplicate structure has been recorded. For a destructive analysis *a*, a simple admissibility condition is

(94) $$M_{k,a}^{\mathrm{res}}\geq M_{a}^{\min}+M_{a}^{\mathrm{rep}},$$

where $M_{k,a}^{\mathrm{res}}$ is the mass or volume of preserved material available for reserve use in sediment unit *k*, $M_{a}^{\min}$ is the minimum mass required for the original analysis, and $M_{a}^{\mathrm{rep}}$ is the mass required for one independent repetition or counter-analysis. If Equation (94) cannot be satisfied, the analysis may still be scientifically useful, but it should not be labeled suitable for contested use unless the absence of reserve material is justified and disclosed.

Forensic readiness is reported through a separate Forensic Integrity Grade (FIG), retained as a procedural label for comparability with contested-use terminology. Let $h\in H^{\mathrm{for}}$ index forensic-readiness components such as chain-of-custody, documentation, duplicate design, reserve aliquots, analyte-specific preservation, destructive-analysis logging, blanks, contamination control, and laboratory competence. Each component is scored as $F_{h,k}\in \left[0,1\right]$ and weighted by $w_{h}^{\mathrm{for}}\geq 0$. The forensic-readiness score is

(95) $$F_{k}^{\mathrm{int}}=\frac{\sum_{h\in H^{\mathrm{for}}}w_{h}^{\mathrm{for}}F_{h,k}}{\sum_{h\in H^{\mathrm{for}}}w_{h}^{\mathrm{for}}}.$$

A critical failure indicator $\chi_{k}^{\mathrm{crit}}$ is set to 1 when a defect prevents reliable linkage between the result and the sampled material, for example absent unique identification, broken custody with no explanation, missing location/depth metadata for a disputed sample, or unrecorded destructive consumption of all material. The grade is then assigned as

(96) $${\mathrm{FIG}}_{k}=\left\{\begin{array}{cc} D, & \chi_{k}^{\mathrm{crit}}=1,\\ A, & \chi_{k}^{\mathrm{crit}}=0\;\mathrm{and}\;F_{k}^{\mathrm{int}}\geq 0.85,\\ B, & \chi_{k}^{\mathrm{crit}}=0\;\mathrm{and}\;0.70\leq F_{k}^{\mathrm{int}}<0.85,\\ C, & \chi_{k}^{\mathrm{crit}}=0\;\mathrm{and}\;0.50\leq F_{k}^{\mathrm{int}}<0.70,\\ D, & \chi_{k}^{\mathrm{crit}}=0\;\mathrm{and}\;F_{k}^{\mathrm{int}}<0.50. \end{array}\right.$$

The FIG is deliberately separated from the CSTAI. A site can be Class IV with FIG-A, meaning high activation potential supported by strong evidentiary integrity; it can also be Class IV with FIG-C, meaning a similar activation score but limited suitability for contested interpretation. Conversely, FIG-A does not reduce toxicological activation potential. It only indicates that the sample history, preservation, duplicate design, and analytical record are strong enough to support repeatability and independent scrutiny.

The distinctions among scientific, regulatory, and contested-use implementations are summarized in Table 15.

Preservation rules must remain analyte-specific. Metals, redox-sensitive species, hydrophobic organics, volatile organic compounds (VOCs), PFASs, microplastics, pore water, and bioassay material have different container, temperature, headspace, holding-time, and contamination constraints. The protocol therefore does not prescribe a universal preservation condition. Instead, contested-use CSTAP requires that the preservation rationale be declared before sampling, that incompatible materials be avoided for the target analyte, and that a sealed reserve aliquot be preserved under conditions compatible with the most likely counter-analysis. The relevant principles are consistent with sediment-sampling procedures that emphasize contamination prevention and custody, forensic trace-evidence guidance that prioritizes preservation before potentially destructive testing, geological-evidence standards that distinguish questioned from known/reference material, and laboratory standards that stress competence, impartiality, and consistent operation [50,51,52,53,54,55].

### 2.30. Minimum Data Tiers and Reporting Checklist

The CSTAP can be applied at different information levels, but the declared level must accompany the final class because evidential support changes the interpretation of $\Psi_{k,e}$. A Tier 0 application is a screening exercise based on bulk chemistry and benchmarks. A Tier 1 application adds sedimentological and geochemical modifiers. A Tier 2 application includes direct exposure measurements such as pore water, passive samplers, DGT, interstitial-water toxic units, or AVS–SEM; recent PAH and metal examples show why these exposure-focused variables can resolve ambiguity left by bulk sediment criteria [26,27]. A Tier 3 application includes biological effects, receptor-specific endpoints, event monitoring, or calibrated reactive-transport parameters. The tier is not a quality label and does not alter the primary class; it is a reproducibility statement that tells readers how much independent evidence supports the score.

The minimum evidence required at each data tier is summarized in Table 16.

### 2.31. Evidence-Adequacy Layer and Two-Track Reporting

The exploratory CSTAI class and the evidential strength supporting that class are distinct outputs. This distinction is necessary because a high score derived from measured pore-water concentrations, toxicity tests, and event monitoring is not equivalent to the same score inferred from bulk chemistry and categorical defaults. The CSTAP therefore reports an evidence-adequacy coefficient, denoted by $K_{k,e}^{\mathrm{evi}}$, alongside the activation score. The evidence coefficient is not a toxicity multiplier and does not alter $\Psi_{k,e}$; it grades how well the modules are supported by direct, reproducible, and scenario-relevant observations.

For the six CSTAP modules, let m=1,…,6 denote chemical burden, bioavailability, mixture pressure, activation forcing, receptor vulnerability, and uncertainty characterization. Each module receives a coverage score $D_{m,k,e}^{\mathrm{cov}}\in \left[0,1\right]$, where 0 denotes an absent or purely assumed input, 0.5 denotes an indirect proxy, and 1 denotes a direct or independently constrained measurement. With non-negative module weights $w_{m}^{\mathrm{evi}}$ satisfying $\sum_{m=1}^{6}w_{m}^{\mathrm{evi}}=1$, evidence adequacy is defined as

(97) $$K_{k,e}^{\mathrm{evi}}=\sum_{m=1}^{6}w_{m}^{\mathrm{evi}}D_{m,k,e}^{\mathrm{cov}}.$$

When no site-specific rationale is available, equal weights $w_{m}^{\mathrm{evi}}=1/6$ should be used. Differential weights are admissible only when declared before scoring, for example when a dredging assessment gives greater evidential weight to activation forcing and stratigraphic accessibility than to long-term benthic-community descriptors.

The reporting rule is two-track:

(98) $${\mathrm{Output}}_{k,e}=\left(C_{k,e},\Psi_{k,e},K_{k,e}^{\mathrm{evi}},q_{k,e},g_{k,e}\right),$$

where $C_{k,e}$ is the exploratory CSTAI class, $q_{k,e}$ is the vector of contaminant contribution shares, and $g_{k,e}$ is the vector of passed or failed quality-control gates. Equation (98) prevents evidence deficiency from being hidden inside a single numerical class: the same exploratory Class IV score may be reported as Class IV–A when evidence is direct and complete, or as Class IV–D when the class is driven mainly by conservative assumptions.

The evidence-adequacy grades used in two-track reporting are given in Table 17.

To prevent the protocol from becoming an opaque index, each application should report the items listed in the complete reporting checklist provided as Supplementary Table S6. The checklist identifies the minimum information required for another analyst to reproduce or audit the score.

### 2.32. Calibration, Inverse Use, and Validation Logic

The CSTAP can be used in two modes. In forward mode, the user supplies chemistry, matrix descriptors, event descriptors, and receptor weights; the protocol returns $\Psi_{k,e}$, its exploratory class, and separate evidence and uncertainty reports. In inverse or calibration mode, independent evidence of toxicity is used to estimate coefficients or class thresholds. Let $Y_{k,e}^{\mathrm{tox}}=1$ denote an observed adverse outcome under site–scenario pair (k,e), such as significant whole-sediment toxicity, pore-water toxicity, impaired benthic community condition, or a validated biomarker response, and let $Y_{k,e}^{\mathrm{tox}}=0$ denote absence of such evidence under the same decision rule. A parsimonious calibration layer is

(99) $$P_{k,e}^{\mathrm{tox}}=\mathrm{Pr}\;\left(Y_{k,e}^{\mathrm{tox}}=1|\Psi_{k,e}\right)={\left[1+\exp\left[-(\varrho_{0}+\varrho_{1}\Psi_{k,e})\right]\right]}^{-1},$$

where $\varrho_{0}$ and $\varrho_{1}$ are calibration parameters and $\varrho_{1}>0$ is required for consistency with the monotonicity proposition. Equation (99) is the primary likelihood layer for future endpoint-specific calibration. Equation (91) should be read as its regularized extension, not as an alternative calibration route. The likelihood does not convert the CSTAP into a purely statistical black box; it provides a formal bridge between the theoretical score and future empirical validation, analogous to logistic sediment-chemistry–toxicity models but with explicit bioavailability and activation terms [8,10,11,12].

If a calibration dataset is available, the parameter vector ϑ can be estimated by constrained minimization of the negative log-likelihood

(100) $$L^{\mathrm{cal}}\left(\vartheta ,\varrho_{0},\varrho_{1}\right)=-\sum_{k,e}\left[Y_{k,e}^{\mathrm{tox}}\ln P_{k,e}^{\mathrm{tox}}+\left(1-Y_{k,e}^{\mathrm{tox}}\right)\ln\left(1-P_{k,e}^{\mathrm{tox}}\right)\right]+\lambda \;R\left(\vartheta \right),$$

subject to the admissibility constraints defined above. The regularization term R(ϑ) penalizes implausible coefficients, and λ≥0 controls the strength of the penalty. In the absence of a calibration dataset, coefficients should be reported as protocol defaults, expert-elicited values, or conservative screening values, and the evidence basis should be downgraded or flagged accordingly. This calibration layer is important for peer review because it shows exactly how CSTAP can be falsified, refined, and transferred from a theoretical protocol to a calibrated procedure after external testing.

Predictive validation should be reported with metrics that are interpretable for binary or ordinal toxicity outcomes. For a validation set of $N_{\mathrm{val}}$ independent site–scenario observations, the Brier score (BS) is

(101) $$\mathrm{BS}=\frac{1}{N_{\mathrm{val}}}\sum_{n=1}^{N_{\mathrm{val}}}{\left({\hat{p}}_{n}-y_{n}\right)}^{2},$$

where ${\hat{p}}_{n}$ is the calibrated probability of adverse effect, and $y_{n}\in \left\{0,1\right\}$ is the observed outcome. Calibration can also be summarized by a bin-wise expected calibration error (ECE),

(102) $$\mathrm{ECE}=\sum_{b=1}^{N_{\mathrm{bin}}}\frac{n_{b}}{N_{\mathrm{val}}}\left|{\bar{p}}_{b}-{\bar{y}}_{b}\right|,$$

where $n_{b}$ is the number of observations in probability bin *b*, ${\bar{p}}_{b}$ is the mean predicted probability in that bin, and ${\bar{y}}_{b}$ is the observed event frequency. Equations (101) and (102) make the protocol empirically contestable: a low score with high observed toxicity, or a high score with repeatedly absent toxicity, identifies the module that must be recalibrated or rejected.

The validation metrics and their intended roles are summarized in Table 18.

### 2.33. Decision-Theoretic Translation

The CSTAP can organize decision-relevant information without replacing regulatory judgement. Once an uncertainty distribution over exploratory CSTAI classes is available after calibration, candidate follow-up options can be compared by expected loss rather than by the point estimate alone. Let C={I,II,III,IV,V} denote the ordered class set, and let a∈A denote a candidate action, such as routine monitoring, additional pore-water characterization, constrained dredging, capping, removal, or no-disturbance management. Define Λ(a,c) as the loss assigned to action *a* when the true class is *c*. The decision score is

(103) $$D_{k,e}\left(a\right)=\sum_{c\in C}\Lambda \left(a,c\right)\mathrm{Pr}\left(C_{k,e}=c|x_{k,e}\right),$$

and the protocol-preferred action is

(104) $$a_{k,e}^{⋆}=\arg\min_{a\in A}D_{k,e}\left(a\right).$$

This layer is optional, because the loss matrix is a policy choice, not a chemical law. It is nevertheless useful in dredging and remediation planning because false-negative errors in Class IV–V sediments may have much greater ecological cost than false-positive precaution in Class II–III sediments. The loss matrix must therefore be reported explicitly whenever Equations (103) and (104) are used.

### 2.34. Scenario-Based Sensitivity Analysis

A scenario-based sensitivity analysis was constructed to examine how the CSTAI responds to plausible coastal disturbances. Six scenarios were defined: baseline oxic stability, dredging/resuspension, hypoxia/anoxia, storm/flood runoff, acidification/salinity shift, and combined climate disturbance. For each scenario, activation terms were modified according to the process equations above. The analysis is not intended to estimate a specific real site; it tests the protocol’s internal behavior and shows whether physically interpretable changes produce coherent score shifts.

The scenario definitions used for sensitivity analysis are provided in Supplementary Table S7. They are dimensionless theoretical settings for protocol testing rather than field measurements.

### 2.35. Dimensionless Numerical Stress Test

To verify the internal behavior of the CSTAP independently of any single field dataset, a dimensionless numerical stress test was added. The test uses reference sediment cells, not new environmental measurements. Each cell is defined by a structural pressure term

(105) $$B_{j}^{\mathrm{str}}=\sum_{i=1}^{N_{c}}H_{i,j}\;\beta_{i,j}^{\mathrm{eff}}\;\mu_{i,j}\;V_{i,j,e}^{\mathrm{rt}},$$

where *j* indexes a reference cell. Under scenario *e*, the raw score becomes

(106) $$\Theta_{j,e}=B_{j}^{\mathrm{str}}\;\alpha_{j,e}^{\mathrm{mem}}\;A_{j,e}^{z},\;\Psi_{j,e}=\log_{10}\left(1+\Theta_{j,e}\right),$$

where $\alpha_{j,e}^{\mathrm{mem}}$ is a cell-specific activation multiplier, and $A_{j,e}^{z}$ is the stratigraphic accessibility factor. Equation (106) is a reduced form of Equation (83); it is used only to test whether the protocol behaves coherently when contamination, bioavailability, event forcing, and accessibility are varied in controlled combinations.

Four reference cells were used: a reduced metal-rich harbour mud, a hydrophobic-organic industrial mud, a PFAS–microplastic estuarine mud, and a buried legacy core interval. These archetypes were chosen because they represent distinct physical and chemical routes to activation. The expected behavior is not that all cells become critical under all events but that class shifts occur when the event modifies the controlling process: oxidation and resuspension for metal sulfides, desorption and ingestion for hydrophobic organics, salinity and particle-mediated transport for PFAS–plastic co-occurrence, and dredging-depth accessibility for buried legacy layers.

### 2.36. Illustrative Applications to Published Mediterranean Datasets

Published datasets were used only to illustrate CSTAP encoding and not to conduct an evidence-synthesis article. Four Mediterranean cases were selected because they represent distinct contaminant and process archetypes: Mar Piccolo of Taranto for trace elements, contaminated sediment thickness, and climate-sensitive resuspension; Augusta Harbour for legacy industrial Hg, Ba, PCBs, and hexachlorobenzene (HCB) in cores; the southeastern Gulf of Trieste for sedimentary microplastics; and Bagnoli–Coroglio/Pozzuoli coastal sediments for mixed industrial and geogenic contamination. These cases were chosen to illustrate how the CSTAP can accommodate metals, legacy organics, emerging particle-bound contaminants, stratified contamination, hydrodynamic activation, and complex source settings using already published information [82,83,84,85,86,87,88].

For each case, the protocol was applied qualitatively by mapping reported contaminant classes, sedimentological controls, and disturbance pathways onto the CSTAP modules. Where numerical values were not consistently available across all modules, the demonstration used categorical scoring rather than continuous CSTAI calculation. This approach preserves transparency: it shows how a site enters the protocol, identifies which modules are data-rich or data-poor, and defines what measurements would be needed for quantitative CSTAI calibration.

## 3. Protocol Verification and Illustrative Outputs

### 3.1. Reading CSTAP Outputs

The outputs reported here are protocol outputs, not new field measurements and not empirical performance estimates. The primary track produces the dimensionless score $\Theta_{k,e}$, the log-transformed score $\Psi_{k,e}$, and the exploratory CSTAI class. The secondary tracks report evidence adequacy, uncertainty priority, and forensic readiness. These secondary tracks qualify the interpretation but do not change the primary class. This separation is the main safeguard against treating sparse information, procedural weakness, or contested-use limitations as toxicity evidence.

A concise guide to the interpretation of the parallel CSTAP outputs is provided in Table 19.

### 3.2. Baseline Comparison and Internal Behavior

The CSTAP was internally compared with simple reference calculations: bulk SQG exceedance, exposure-focused quotients, additive toxic units, and event-free core scoring. These baselines are not competitors to be discarded; they are reference cases that show what the protocol adds. Bulk comparison identifies chemical concern. Exposure-focused quotients show whether the same burden is biologically accessible. Additive toxic units represent mixture pressure without non-additive assumptions. Event-free scoring defines the baseline state against which dredging, resuspension, hypoxia, salinity change, acidification, or warming are compared.

The internal checks support four expected behaviors. First, if $C_{i,k}^{\mathrm{tot}}=0$ for all contaminants, the score is zero, and a disturbance cannot create contaminant-driven activation potential. Second, high bulk burden alone does not imply a high class when exposure, accessibility, and activation terms are weak. Third, event forcing without contaminant inventory is reported as physical disturbance, not toxicological activation potential. Fourth, uncertainty, evidence adequacy, and forensic readiness qualify the output without altering the primary class. Extended stress-test and scenario-response tables are retained in the Supplementary Tables because they describe protocol behavior rather than field observations.

### 3.3. Worked Example and Threshold Translation

The worked example illustrates arithmetic reproducibility. The supplied Python/CSV implementation reproduces the core calculation for a theoretical harbour sediment cell: Θ=12.1605, Ψ=1.11927, and $\Pi^{\mathrm{prio}}=13.9846$, yielding exploratory Class IV for the declared inputs. The example is intentionally dimensionless and does not represent a measured site. It verifies input–output consistency, contribution accounting, and the separation of score from uncertainty-priority reporting.

The log transformation makes the thresholds interpretable. The class limits Ψ=0.30, 0.70, 1.10, and 1.50 correspond to approximately Θ=1.00, 4.01, 11.59, and 30.62, respectively. These thresholds are provisional defaults for protocol testing and must not be interpreted as universal ecological or regulatory boundaries.

### 3.4. Illustrative Mediterranean Encoding

Published Mediterranean cases were encoded only to show how different evidence types activate different modules. Mar Piccolo of Taranto illustrates trace-element burden, contaminated thickness, and potential resuspension sensitivity. Augusta Harbour illustrates industrial legacy contamination and disturbance-sensitive Hg and organic contaminants. The Gulf of Trieste illustrates microplastic retention and the need for route-specific particle metrics. Bagnoli–Coroglio/Pozzuoli illustrates the need to separate anthropogenic burden from geogenic and hydrothermal background. These examples do not validate CSTAI classes. They show how a future calibrated implementation would declare modules, data tiers, uncertainty, and missing evidence before drawing operational conclusions [82,83,85,86,87,88,89].

### 3.5. Current Evidential Status

The present outputs illustrate coherence, dimensional closure, reproducible arithmetic, and defensible reporting logic. They do not demonstrate predictive accuracy. The protocol becomes empirical only when $\Psi_{k,e}$ and its component terms are calibrated against paired chemistry, exposure, bioassay, and ecological-effect datasets. Until then, the CSTAP should be used to organize hypotheses, compare scenarios, locate data gaps, and design validation studies.

## 4. Discussion

### 4.1. Scientific Contribution and Boundaries

The CSTAP shifts the assessment question from static contamination status to event-conditioned toxicological activation potential. This shift does not weaken sediment-quality screening. It clarifies its boundary conditions. A bulk benchmark exceedance remains a useful warning, but it does not state whether the contaminant is bioavailable, mobilizable, route-accessible, or biologically consequential under a given scenario. The CSTAP makes those intermediate assumptions explicit and auditable.

The contribution is structural. The protocol separates state variables from process modifiers and separates the primary score from evidence, uncertainty, and procedural integrity. That separation is essential because high activation potential can arise from different causes: high chemical burden, independent exposure evidence, disturbance forcing, receptor sensitivity, or conservative defaults. A defensible application should therefore report not only the class but also the dominant terms and the evidential tier that produced it.

### 4.2. Bioavailability, Mixtures, and Parameter Governance

Bioavailability is treated as a state variable, not as a universal contaminant constant. The information-source safeguard is therefore central: when exposure information is inferred only from the same bulk concentration used in $H_{i,k}$, $\beta_{i,k}^{\mathrm{eff}}$ returns to unity and the calculation remains burden-based. Independent pore-water measurements, passive samplers, DGT, AVS–SEM, or well-declared sediment properties can justify stronger exposure terms.

Mixture pressure is handled conservatively. Additivity is the default, and non-additive terms should be used only when supported by experimental evidence, field evidence, or a transparent expert declaration. This is particularly important for PFASs, microplastics, nanoplastics, and particle-associated contaminants, where exposure metrics and biological routes differ from those used for neutral hydrophobic organics or dissolved metals. The CSTAP can organize such evidence, but it does not make heterogeneous contaminant classes automatically commensurable.

### 4.3. Event Conditioning and Climate-Relevant Scenarios

The most distinctive use of the CSTAP is event-conditioned assessment. Dredging, resuspension, hypoxia, reoxygenation, salinity change, acidification, warming, and storm runoff can alter partitioning, lability, and biological contact. The protocol treats these events as declared scenarios rather than generic concern multipliers. This is why the redox gate is bidirectional, the mass-conservation ceiling is limited to mass-compatible exposure metrics, and class shifts must be traced to physical transport, chemical gates, stratigraphic accessibility, or receptor contact. Climate-related forcing justifies scenario design; it does not replace site-specific calibration.

### 4.4. Forensic Readiness and Reproducibility

The forensic-readiness layer is optional and procedural. It documents whether samples, splits, reserve aliquots, blanks, preservation records, custody forms, and destructive-analysis logs can support repeat analysis or contested interpretation. It is not a toxicity multiplier and cannot change the CSTAI class. This separation keeps the ecotoxicological calculation distinct from evidentiary defensibility while still acknowledging that sediment results are often used in contested management settings.

Reproducibility is supported by the Supplementary Reproducibility Package, which provides a minimal Python implementation, CSV inputs, expected outputs, a parameter dictionary, and a checksum manifest for the worked example. The package reproduces the core arithmetic only. Optional modules require site-specific implementation and independent validation before predictive use. A future repository DOI would further improve traceability and reuse in line with FAIR principles [81].

### 4.5. Limitations and Validation Roadmap

The present protocol is not externally validated. The class thresholds are provisional defaults, the class-assignment uncertainty outputs are summaries rather than validated probability forecasts, and the calibration equations are prospective. These limitations are not cosmetic; they define the current scientific status of CSTAP.

Validation should be endpoint-specific. Acute toxicity, chronic benthic response, bioaccumulation, trophic transfer, and community alteration should not be collapsed into a single outcome before separate calibration. A useful validation dataset would include $C_{i,k}^{\mathrm{tot}}$, pore-water or passive-sampler exposure metrics, AVS–SEM or DGT for metals, TOC, black carbon, grain size, pH, $E^{h}$, salinity, oxygen, disturbance descriptors, bioassays, and benthic-community observations before and after a known event. Retrospective multi-site calibration, prospective resuspension/redox experiments, field event studies, and repeatability tests using split and archive aliquots would allow the CSTAP to move from theoretical protocol to calibrated procedure.

The minimum paired dataset recommended for first endpoint-specific calibration is listed in Table 20.

## 5. Conclusions

The CSTAP provides a theoretical protocol for organizing the transition from coastal sediment contamination to event-conditioned toxicological activation potential. Its main contribution is to separate stored burden, effective exposure, disturbance forcing, receptor vulnerability, evidential support, uncertainty priority, and procedural integrity into distinct reporting components. The CSTAI is the associated exploratory score. It is useful for hypothesis generation, scenario comparison, monitoring design, baseline-versus-event reporting, and calibration planning.

The protocol does not provide externally validated class prediction. CSTAI classes remain exploratory until tested against paired chemistry, exposure metrics, bioassays, and ecological observations across sites and event regimes. The next step is endpoint-specific calibration, uncertainty propagation, sensitivity ranking, and external testing under contrasting sedimentological, geochemical, and management contexts. Until then, the CSTAP should be used to make assumptions, data gaps, and event-conditioned activation pathways explicit, not to replace biological evidence or regulatory assessment.

## Figures and Tables

**Table 1:** Protocol invariants and empirical hypotheses generated by the Coastal Sediment Toxicological Activation Protocol (CSTAP).

Claim Type and Name	Expected Behavior	Observation or Audit Result Requiring Correction
**Mathematical/implementation invariants**		
Zero-burden sanity check	If all $H_{i,k}=0$, then $\Theta_{k,e}=0$ and $\Psi_{k,e}=0$ irrespective of scenario labels.	A non-zero score produced without a declared contaminant burden.
Conditional monotonicity	Increasing $H_{i,k}$, $\beta_{i,k}^{\mathrm{eff}}$, $\alpha_{i,k,e}$, or $V_{i,k}^{\mathrm{rec}}$ while holding all other terms fixed, or while restricting mixture terms to non-antagonistic values, cannot lower $\Psi_{k,e}$.	A verified implementation in which a positive burden or exposure increment decreases the score without a compensating reduction in another declared multiplier.
Reporting-layer separation	$U_{k}$, $K_{k,e}^{\mathrm{evi}}$, and the Forensic Integrity Grade (FIG), ${\mathrm{FIG}}_{k}$, qualify interpretation but do not alter $\Theta_{k,e}$, $\Psi_{k,e}$, or the primary class boundaries.	An implementation in which missing evidence, high uncertainty, or weak custody records alone changes the primary class without changing a declared scoring term.
**Empirical hypotheses for future calibration**		
Partition control	At fixed $C_{i,k}^{\mathrm{tot}}$, $\varepsilon_{k}$, and $\rho_{b,k}$, increasing $K_{i,k}^{d}$ should decrease the equilibrium estimate of $C_{i,k}^{\mathrm{pw}}$.	Paired sediment–pore-water measurements showing systematic increases in exposure with increasing effective partitioning after confounders are removed.
Trigger specificity	Disturbance should change class only by changing transport, chemical gates, biological contact, stratigraphic accessibility, or receptor contact.	A class shift caused by an event descriptor that has no declared physical, chemical, stratigraphic, or biological-contact pathway.
Matrix dependence	Equal bulk concentrations may yield different classes when partitioning, redox, mixture structure, receptor setting, or disturbance forcing differs.	Matched datasets in which these variables vary substantially but measured exposure and effect probabilities remain invariant within uncertainty.
Calibration slope	In endpoint-specific calibration, the fitted relationship between $\Psi_{k,e}$ and observed adverse-response labels should have a positive slope if the score is informative for that endpoint.	Calibrated slope estimates that are zero, negative, or unstable across independent sites and endpoints.

**Table 2:** Core indices and concentration variables used in the CSTAP and CSTAI. The notation is kept invariant throughout the manuscript.

Symbol	Meaning	Unit	Comment
*i*	Contaminant index	–	Metals, metalloids, PAHs, PCBs, PFASs, TBT, microplastics, and other contaminants.
*k*	Site or sediment unit index	–	Station, core interval, management cell, or spatial grid cell.
*r*	Ecological receptor index	–	Amphipods, polychaetes, bivalves, echinoderm larvae, demersal fish, or food-web compartments.
*e*	Disturbance scenario index	–	Stable, dredging, storm, hypoxia, acidification, salinity shift, or combined scenario.
*g*	Exposure-route index	–	Pore-water uptake, sediment ingestion, particle contact, or trophic/food-web transfer route.
$C_{i,k}^{\mathrm{tot}}$	Total sediment concentration	mass mass^−1^ dry sediment	Primary chemical-burden input.
$Q_{i}$	Sediment guideline or reference value	same as $C_{i,k}^{\mathrm{tot}}$	SQG, background-corrected threshold, site-specific benchmark, or regulatory value.
$H_{i,k}$	Hazard ratio	dimensionless	$H_{i,k}=C_{i,k}^{\mathrm{tot}}/Q_{i}$.
$C_{i,k}^{\mathrm{pw}}$	Pore-water concentration	mass volume^−1^	Mechanistic exposure proxy for dissolved or labile contaminants.
$K_{i,k}^{d}$	Solid–water distribution coefficient	volume mass^−1^	Site- and contaminant-specific partitioning coefficient.
$\varepsilon_{k}$	Sediment porosity	dimensionless	Pore-water volume per bulk sediment volume.
$\rho_{b,k}$	Dry bulk density	mass volume^−1^	Dry solid mass per bulk sediment volume.

**Table 3:** Core multipliers, weights, and CSTAI scores used in CSTAP. Symbols are not reused for distinct quantities.

Symbol	Meaning	Unit	Comment
$\beta_{i,k}$	Bioavailability multiplier	dimensionless	Increases when a larger fraction is dissolved, labile, or chemically active.
$I_{i,k}^{\mathrm{src}}$	Information-source safeguard coefficient	dimensionless	Governance coefficient indicating whether the bioavailability term is supported by a source separable from $H_{i,k}$; it is not a formal statistical-independence measure.
$\beta_{i,k}^{\mathrm{eff}}$	Effective bioavailability multiplier	dimensionless	$\beta_{i,k}^{\mathrm{eff}}=1+I_{i,k}^{\mathrm{src}}\left(\beta_{i,k}-1\right)$; used in CSTAI to reduce double counting.
$\mu_{i,k}$	Mixture multiplier	dimensionless	Encodes additive, synergistic, or antagonistic co-occurrence.
$\alpha_{i,k,e}$	Activation multiplier	dimensionless	Scenario-dependent effect of disturbance on exposure.
$\zeta_{i,r}$	Receptor vulnerability coefficient	dimensionless	Sensitivity of receptor *r* to contaminant *i*.
$\lambda_{r,k}^{\mathrm{rec}}$	Receptor relevance weight	dimensionless	Site-specific ecological relevance; $\sum_{r}\lambda_{r,k}^{\mathrm{rec}}=1$.
$V_{i,k}^{\mathrm{rec}}$	Unrouted receptor-weighted sensitivity	dimensionless	$V_{i,k}^{\mathrm{rec}}=\sum_{r}\lambda_{r,k}^{\mathrm{rec}}\zeta_{i,r}$; fallback term when route data are unavailable.
$V_{i,k,e}^{\mathrm{rt}}$	Route-resolved receptor vulnerability	dimensionless	Combines receptor sensitivity, receptor relevance, route weights, and route accessibility under scenario *e*.
$U_{k}$	Uncertainty-priority coefficient	dimensionless	Records information deficiency for conservative management prioritization; reported separately from the activation score.
$\Pi_{k,e}^{\mathrm{prio}}$	Optional management-priority score	dimensionless	$\Pi_{k,e}^{\mathrm{prio}}=U_{k}\Theta_{k,e}$; not interpreted as measured toxicity.
$\Theta_{k,e}$	Raw CSTAI activation score	dimensionless	Multiplicative sum across contaminants and receptors.
$\Psi_{k,e}$	Log-transformed CSTAI score	dimensionless	$\Psi_{k,e}=\log_{10}\left(1+\Theta_{k,e}\right)$.
$\Omega_{i,k,e}$	Contaminant-specific score contribution	dimensionless	Product term entering the summation for $\Theta_{k,e}$.
$q_{i,k,e}^{\mathrm{con}}$	Contribution share	dimensionless	Fraction of the summed score attributable to contaminant *i* under scenario *e*.
$P_{k,e}^{\mathrm{tox}}$	Calibrated probability of adverse effect	dimensionless	Logistic validation output when independent toxicity observations are available.
$K_{k,e}^{\mathrm{evi}}$	Evidence-adequacy coefficient	dimensionless	Weighted completeness of the evidence supporting a site–scenario class; reported separately from $\Psi_{k,e}$.
$D_{m,k,e}^{\mathrm{cov}}$	Module-level evidence coverage	dimensionless	Coverage score for protocol module *m*; bounded between 0 and 1.
$w_{m}^{\mathrm{evi}}$	Evidence weight of module *m*	dimensionless	Weight used only to grade evidential adequacy; $\sum_{m}w_{m}^{\mathrm{evi}}=1$.

**Table 4:** Benchmark hierarchy for defining $Q_{i}$ before calculating $H_{i,k}$. The highest defensible level should be used, and lower levels should be reported as sensitivity checks when they materially change the exploratory CSTAI class.

Level	Benchmark Source	Preferred Use	Required Disclosure
A	Site-specific effect threshold calibrated against local toxicity tests or benthic responses.	Tier 3 assessments and regulatory decisions with local validation.	Endpoint, receptor, calibration dataset, statistical model, and confidence interval.
B	Established marine, estuarine, or freshwater SQG matching the sediment matrix and contaminant form.	Tier 0–2 screening and inter-site comparison.	Guideline family, matrix, dry-weight basis, grain-size fraction, and applicability limits.
C	Regional background, reference-site concentration, or background-corrected threshold.	Naturally enriched settings, geogenic inputs, and mixed anthropogenic–geogenic sources.	Background derivation, reference-site selection, and enrichment rule.
D	Equilibrium-partitioning or pore-water benchmark converted to a sediment value using $K_{i,k}^{d}$.	Contaminants for which exposure metrics are stronger than bulk sediment guidelines.	Partitioning model, $K_{i,k}^{d}$ source, pore-water reference value, and evidence/uncertainty flag.

**Table 5:** Physical and mathematical admissibility constraints used in the default CSTAP implementation. Bounds may be narrowed by site-specific calibration but should not be violated without explicit justification.

Quantity	Default Admissible Range	Reason for Constraint
$C_{i,k}^{\mathrm{tot}}$, $C_{i,k}^{\mathrm{pw}}$, $C_{i,k}^{\mathrm{free}}$	≥0	Concentrations and exposure metrics cannot be negative.
$\varepsilon_{k}$	$0<\varepsilon_{k}<1$	Porosity is a volume fraction.
$\rho_{b,k}$, $K_{i,k}^{d}$, $C_{i,r}^{\mathrm{eff}}$	>0	Density, distribution coefficients, and effect references enter denominators.
$H_{i,k}$, ${\mathrm{TU}}_{i,k,r}$, $\Theta_{k,e}$, $\Psi_{k,e}$	≥0	Hazard, toxic-unit, and CSTAI scores are non-negative by construction.
$\beta_{i,k}^{\mathrm{eff}}$, $\alpha_{i,k,e}$	≥1 in default screening	Default use treats bioavailability and activation as amplifying or neutral factors; calibrated applications may report attenuating factors separately.
$U_{k}$	≥1, reported separately	Indicates uncertainty-driven priority for additional evidence; it does not alter the primary activation score.
$\mu_{i,k}$	$\mu_{i}^{\min}\leq \mu_{i,k}\leq \mu_{i}^{\max}$	Prevents unsupported extrapolation of mixture synergy or antagonism.
$\lambda_{r,k}^{\mathrm{rec}}$	$0\leq \lambda_{r,k}^{\mathrm{rec}}\leq 1$, $\sum_{r}\lambda_{r,k}^{\mathrm{rec}}=1$	Receptor weights form a normalized ecological relevance vector.
$\nu_{g,r}^{\mathrm{rt}}$	$0\leq \nu_{g,r}^{\mathrm{rt}}\leq 1$, $\sum_{g\in G^{\exp}}\nu_{g,r}^{\mathrm{rt}}=1$	Route weights form a receptor-specific exposure simplex.
$A_{i,k,e,g}^{\mathrm{rt}}$	$0\leq A_{i,k,e,g}^{\mathrm{rt}}\leq 1$ in the default bounded form	Route accessibility cannot exceed its normalized reference ceiling unless explicitly declared exploratory.
$A_{k,e}^{z}$	$0\leq A_{k,e}^{z}\leq 1$	Stratigraphic accessibility is a bounded fraction of layer-specific activation potential.
$Z_{i,k,e}^{\mathrm{act}}\left(t\right)$	$0\leq Z_{i,k,e}^{\mathrm{act}}\left(t\right)\leq 1$	Temporal activation memory is a bounded state variable and cannot exceed full activation.
$f_{i,k,e}^{\mathrm{mob}}$, $\eta_{i,k,e}^{\mathrm{sol}}$, $\eta_{k,e}^{\mathrm{pw}}$	0≤·≤1	Mobilization and release efficiencies are bounded fractions of the accessible inventory.
$C_{i,k,e}^{\mathrm{ceil}}$, $C_{i,k,e}^{\mathrm{evt}}$	≥0 and $C_{i,k,e}^{\mathrm{evt}}\leq C_{i,k,e}^{\mathrm{ceil}}$	Event concentrations cannot exceed the mobilizable mass divided by the scenario mixing volume.
$P_{k,e}^{\mathrm{tox}}$	$0\leq P_{k,e}^{\mathrm{tox}}\leq 1$	Calibrated toxicity probability must be probabilistically admissible.

**Table 6:** Minimal examples for the information-source safeguard. The examples are illustrative rules for first-pass implementation rather than calibrated parameter estimates.

Evidence Used for β	Source Relation to *H*	Typical $I^{\mathrm{src}}$	Interpretation
Bulk concentration only	Same signal already used in $H_{i,k}$	0	$\beta_{i,k}^{\mathrm{eff}}=1$; output remains burden-based.
Equilibrium partitioning (EqP) using $C_{i,k}^{\mathrm{tot}}$ plus measured total organic carbon (TOC), black carbon (BC), porosity, or AVS–SEM	Partly independent	0.25–0.75	Exposure modifier may be used, but the inherited bulk signal is discounted.
Measured pore water, passive sampler, DGT flux, or independent AVS–SEM diagnostic	Independent exposure evidence	0.75–1	$\beta_{i,k}^{\mathrm{eff}}$ may approach the raw exposure multiplier.

**Table 7:** Operational governance rules for $I_{i,k}^{\mathrm{src}}$, $\beta_{i,k}^{\mathrm{eff}}$, $V_{i,k,e}^{\mathrm{rt}}$, $U_{k}$, and $K_{k,e}^{\mathrm{evi}}$. These rules separate physicochemical availability, receptor-route access, uncertainty priority, and evidential adequacy.

Quantity	Admissible Assignment	Reporting Rule
$I_{i,k}^{\mathrm{src}}=1$	$\beta_{i,k}$ is based on pore water, passive sampling, DGT, AVS–SEM, or another exposure line independent of $C_{i,k}^{\mathrm{tot}}$.	Full bioavailability amplification can enter $\beta_{i,k}^{\mathrm{eff}}$, unless the same evidence is also used in $V_{i,k,e}^{\mathrm{rt}}$.
$0<I_{i,k}^{\mathrm{src}}<1$	$\beta_{i,k}$ is derived from EqP or a proxy that combines $C_{i,k}^{\mathrm{tot}}$ with independently measured sediment properties such as organic carbon, black carbon, sulfides, porosity, pH, or salinity.	Report the proxy and the basis for partial independence; sensitivity to $I_{i,k}^{\mathrm{src}}$ is recommended.
$I_{i,k}^{\mathrm{src}}=0$	No exposure information beyond bulk concentration is available.	Set $\beta_{i,k}^{\mathrm{eff}}=1$; record the missing exposure evidence through $K_{k,e}^{\mathrm{evi}}$ and $U_{k}$.
$V_{i,k,e}^{\mathrm{rt}}$	Route term based on receptor presence, route contact, ingestion/contact/trophic relevance, and biological vulnerability.	Do not reuse the same exposure measurement already used for $\beta_{i,k}^{\mathrm{eff}}$ unless source separation is declared.
$U_{k}$	Uncertainty-priority coefficient based on missing variables, proxy use, spatial coverage, and analytical limitations.	Report separately or use only in optional $\Pi_{k,e}^{\mathrm{prio}}$; it does not change $\Psi_{k,e}$ or the primary class.
$K_{k,e}^{\mathrm{evi}}$	Evidence-adequacy coefficient based on independent support for burden, exposure, activation, receptor, and scenario components.	Report as confidence/evidence support; it cannot compensate for or reduce the primary score.

**Table 8:** Governance of the main free or weakly identified parameters. Defaults are conservative screening rules and do not replace endpoint-specific calibration.

Parameter or Term	Default/Admissible Use	Evidence Requirement
$\gamma_{ij}$	0 for unknown interactions; non-zero values only in the declared interaction matrix; $\mu_{i}^{\min}>0$.	Co-stressor evidence, shared mode of action, or experimental/field support; no ordinary co-occurrence amplification.
$\Lambda_{i}$	Upper activation increment; caps admitted activation at $1+\Lambda_{i}$.	Screening default or contaminant-specific calibration.
$\chi_{i}^{\mathrm{phys}},\chi_{i}^{\mathrm{chem}},\chi_{i}^{\mathrm{bio}},\chi_{i}^{z}$	Component-specific non-negative activation sensitivities for physical, chemical, biological-contact, and stratigraphic activation.	Event-response calibration or conservative sensitivity analysis; report which component is used.
$g_{i,k,e}^{\mathrm{chem}}$	Positive dimensionless chemical gate with signed sensitivity coefficients; not a speciation model.	pH, salinity, redox, phase, or contaminant-class rationale.
$g_{k,e}^{\mathrm{bio}}$	Non-negative biological-contact gate; bounded in screening.	Bioturbation, bioirrigation, resuspension contact, or route evidence.
$s_{i}^{\mathrm{pH}},s_{i}^{\mathrm{sal}},s_{i}^{\mathrm{red}}$	Signed sensitivity coefficients.	Direction must be contaminant-specific and documented.
$\omega_{i}^{T},\omega_{i}^{O}$	Bounded temperature and oxygen sensitivities; default mode cannot attenuate the score.	Calibrated laboratory or field evidence for attenuation or amplification.
$\zeta_{i,r},\kappa_{i,r,g}^{\mathrm{rt}}$	Default 1 unless receptor or route evidence supports another value.	Species sensitivity, route-specific uptake, bioassay, or ecological-protection goal.

**Table 9:** Default bounded ordinal mapping from categorical 0–4 screening scores to neutral or amplifying CSTAP multipliers. These entries are governance defaults for data-limited Core applications, not biological effect thresholds or calibrated toxicity coefficients; measured or endpoint-calibrated process functions supersede them whenever available.

Category	Meaning	Default Multiplier Use	Reporting Rule
0	absent or not applicable	0 only for burden; 1 for neutral multipliers	Distinguish absence of contaminant from missing evidence.
1	low	1.00	No amplification relative to the neutral screening baseline.
2	moderate	1.25	Use only when a declared proxy supports moderate amplification.
3	high	1.50	Requires a declared process rationale or measured proxy.
4	very high	2.00 or calibrated cap	Requires sensitivity analysis or a calibrated cap.

**Table 10:** Exploratory CSTAI classes based on the log-transformed score $\Psi_{k,e}=\log_{10}\left(1+\Theta_{k,e}\right)$. Thresholds are provisional defaults for protocol testing only and require endpoint-specific calibration before predictive or regulatory use.

Class	Range of $\Psi_{k,e}$	Name	Interpretation
I	$\Psi_{k,e}<0.30$	Low protocol signal	Contaminant burden and exposure modifiers are low within the assessed scenario.
II	$0.30\leq \Psi_{k,e}<0.70$	Moderate protocol signal	Stored contamination or moderate exposure relevance is indicated; targeted measurements may improve evidence adequacy.
III	$0.70\leq \Psi_{k,e}<1.10$	Elevated protocol signal	Chemical burden and/or exposure relevance appear substantial even without major disturbance; bioassays and pore-water measurements are appropriate for confirmation.
IV	$1.10\leq \Psi_{k,e}<1.50$	Event-sensitive protocol signal	Disturbance, redox change, or mixture pressure may shift the sediment toward higher activation potential; confirmatory event-specific evidence is required before operational inferences are drawn.
V	$\Psi_{k,e}\geq 1.50$	Highest exploratory protocol signal	Contamination, exposure relevance, mixture pressure, and activation triggers coincide in the protocol output; the class indicates highest priority for confirmatory testing and site-specific review.

**Table 11:** Raw-score equivalents of the provisional exploratory CSTAI class thresholds, computed from $\Theta =10^{\Psi }-1$.

Boundary	Ψ Threshold	Θ Equivalent	Interpretation
I/II	0.30	1.00	Transition from very low to low/moderate activation-potential signal.
II/III	0.70	4.01	Transition to elevated latent activation-potential signal.
III/IV	1.10	11.59	Transition to event-driven activation-potential concern requiring confirmatory evidence.
IV/V	1.50	30.62	Highest exploratory protocol signal requiring site-specific calibration before operational use.

**Table 12:** Algorithmic implementation of CSTAP for a site *k* and event scenario *e*.

Step	Operation
1	Define the spatial unit *k*, the scenario *e*, the contaminant set $\left\{1,\ldots ,N_{c}\right\}$, and the receptor set $\left\{1,\ldots ,N_{r}\right\}$.
2	Select sediment benchmarks $Q_{i}$ and calculate $H_{i,k}=C_{i,k}^{\mathrm{tot}}/Q_{i}$.
3	Estimate exposure metrics from measured pore water, passive samplers, DGT, AVS–SEM, or Equations (24)–(32); calculate $\beta_{i,k}$, assign $I_{i,k}^{\mathrm{src}}$, and compute $\beta_{i,k}^{\mathrm{eff}}$.
4	Calculate additive toxic units where $C_{i,r}^{\mathrm{eff}}$ exists; otherwise use conservative hazard surrogates and flag the evidence limitation.
5	Define the interaction matrix Γ and calculate $\mu_{i,k}$ with bounded interaction coefficients.
6	Calculate disturbance and chemical gates from $\tau_{b,k,e}$, $\tau_{c,k}$, ${\mathrm{pH}}_{k,e}$, ${\mathrm{Sal}}_{k,e}$, $E_{k,e}^{h}$, $T_{k,e}^{\mathrm{abs}}$, $O_{k,e}^{\mathrm{diss}}$, and event descriptors; obtain $\alpha_{i,k,e}$, $\alpha_{i,k,e}^{\mathrm{TO}}$, $\alpha_{i,k,e}^{\mathrm{mem}}$, or the mass-corrected $\alpha_{i,k,e}^{\mathrm{mc}}$ when the mobilizable-inventory audit is triggered.
7	Assign receptor weights $\lambda_{r,k}^{\mathrm{rec}}$, vulnerability coefficients $\zeta_{i,r}$, route weights $\nu_{g,r}^{\mathrm{rt}}$, and route accessibility factors $A_{i,k,e,g}^{\mathrm{rt}}$; calculate $V_{i,k,e}^{\mathrm{rt}}$ or the fallback $V_{i,k}^{\mathrm{rec}}$.
8	Calculate the activation score $\Theta_{k,e}$ and $\Psi_{k,e}$.
9	Assign the uncertainty-priority coefficient $U_{k}$, calculate the optional priority score $\Pi_{k,e}^{\mathrm{prio}}$, assign evidence adequacy $K_{k,e}^{\mathrm{evi}}$, and optionally propagate stochastic uncertainty using Equations (87) and (88).
10	Assign the exploratory CSTAI class from Table 10 and report the class together with dominant contribution shares $q_{i,k,e}^{\mathrm{con}}$, elasticities, evidence adequacy, missing variables, and indicative follow-up measurements.
11	When the application is regulatory, contested, or otherwise dispute-sensitive, assign ${\mathrm{FIG}}_{k}$ from Equations (95) and (96) and report custody, duplicates, reserve aliquots, preservation records, blanks, and destructive-analysis logs.

**Table 13:** Core, extended, and exploratory CSTAP implementation levels. Each application must declare the level used before the exploratory activation class is interpreted.

Component	Core Implementation	Extended Implementation	Exploratory or Optional Layer
Purpose	Transparent screening, hypothesis generation, and missing-data diagnosis.	Site-specific assessment with measured or independently constrained process terms.	Scenario exploration, contested-sample reporting, advanced mixture or temporal analyses, and calibration planning.
Required input	Site unit, contaminant list, dry-weight concentrations, selected benchmarks, receptor fallback, and declared scenario.	Core inputs plus pore water or passive sampling, AVS–SEM or DGT where relevant, sediment descriptors, event forcing, and route-specific receptor information.	Explicit purpose, declared assumptions, data tier, and a statement that the module is not part of validated prediction.
Default use	$\mu_{i,k}=1$; categorical $\beta_{i,k}^{\mathrm{eff},\mathrm{core}}$ and $\alpha_{i,k,e}^{\mathrm{core}}$; declared receptor fallback.	Defaults should be replaced by measured or independently constrained coefficients wherever possible.	Non-additive mixture terms, temporal memory, decision-theoretic loss functions, and forensic-readiness grades require explicit justification.
Forbidden interpretation	Not a validated toxicity prediction or a regulatory limit.	Not a regulatory decision rule unless calibrated, externally tested, and accepted by the relevant authority.	Not evidence of toxicity and not legal admissibility; used only to expose assumptions, priorities, or procedural integrity.
Output	$\Theta_{k,e}^{\mathrm{core}}$, $\Psi_{k,e}$, provisional class, evidence grade, and missing-variable list.	$\Theta_{k,e}$, $\Psi_{k,e}$, contribution shares, uncertainty propagation, mass-conservation diagnostics, route-resolved outputs, and calibration metrics.	Optional appendices or supplementary outputs, such as FIG, advanced sensitivity audits, and scenario-planning diagnostics.

**Table 14:** Quality-control gates used before assigning a numerical exploratory CSTAI class. A failed gate does not prove low or high toxicity; it identifies a methodological weakness that must be corrected or disclosed.

Gate	Pass Condition	Consequence If Not Satisfied
Dimensional gate	All concentrations, benchmarks, and exposure proxies use compatible units, dry-weight basis, and sediment fraction.	Numerical exploratory CSTAI class is not admissible; only qualitative screening is allowed.
Benchmark gate	$Q_{i}$ is selected from the declared hierarchy before scoring, and sensitivity to alternative benchmarks is documented when relevant.	$H_{i,k}$ is flagged as benchmark-dependent and the low evidence basis must be flagged in $U_{k}$ and $K_{k,e}^{\mathrm{evi}}$.
Exposure gate	$\beta_{i,k}$, $I_{i,k}^{\mathrm{src}}$, and $\beta_{i,k}^{\mathrm{eff}}$ are based on measured pore water, passive sampling, DGT, AVS–SEM, modeled partitioning, or an explicitly declared categorical proxy.	Assessment remains Tier 0–1 if exposure evidence is absent; class must be interpreted as a burden-based hypothesis.
Mixture gate	The contaminant set, mixture model, and interaction coefficients are declared; the no-interaction default is $\mu_{i,k}=1$.	Mixture amplification cannot be claimed; missing mixture evidence enters uncertainty.
Event gate	Each activation scenario changes at least one physical, chemical, biological-contact, stratigraphic, or receptor-contact pathway.	Event-driven class shifts are not admissible for that scenario.
Mass-conservation gate	For erosive or dredging scenarios, $C_{i,k,e}^{\mathrm{evt}}$ is checked against $C_{i,k,e}^{\mathrm{ceil}}$ or the absence of inventory information is explicitly declared.	Unsupported activation multipliers are capped, or the scenario remains qualitative.
Receptor-route gate	Receptor set, receptor weights, route weights, route accessibility proxies, and vulnerability coefficients are stated and linked to the management question.	$V_{i,k,e}^{\mathrm{rt}}$ defaults or $V_{i,k}^{\mathrm{rec}}$ fallbacks must be disclosed and interpreted conservatively.
Stratigraphic gate	For cores or buried deposits, the depth interval and accessibility function $A_{k,e}^{z}$ are reported.	Buried contamination cannot be treated as activated unless erosion, dredging, or bioturbation is specified.
Evidence-adequacy gate	The evidence-adequacy coefficient $K_{k,e}^{\mathrm{evi}}$ and the module coverages $D_{m,k,e}^{\mathrm{cov}}$ are reported.	The class may be used only as a hypothesis-generating screen, not as a stand-alone management endpoint.
Forensic-integrity gate	For contested applications, ${\mathrm{FIG}}_{k}$, custody, duplicate design, reserve aliquots, preservation, blanks, and destructive-analysis logs are reported.	The CSTAI score may remain scientifically useful, but it should not be presented as evidence suitable for contested use.
Traceability gate	Input values, coefficients, scenario settings, missing variables, and contribution shares are reported.	The score is not reproducible and should not be used for management prioritization.

**Table 15:** Scientific, regulatory, and contested-use CSTAP applications. The forensic-readiness reporting layer governs evidentiary defensibility and does not change the CSTAI activation-potential score.

Requirement	Scientific-Grade	Regulatory-Grade	Contested-Use
Spatial and depth documentation	Recommended; sufficient for research interpretation when uncertainty is declared.	Required according to the sampling plan and management question.	Required with unique identifiers, Global Positioning System (GPS)/depth metadata, photographs, core logs where relevant, and recorded deviations.
Field duplicate	Recommended for heterogeneous areas.	Required or justified by the quality assurance/quality control (QA/QC) design.	Required at critical locations to quantify sampling-scale variability.
Split duplicate	Optional unless analytical precision is central.	Recommended for key contaminants and high-consequence decisions.	Required or explicitly justified; used to support repeatability and counter-analysis.
Reserve aliquot or archive core	Optional.	Recommended for confirmatory analysis.	Required for contested locations whenever destructive analysis is planned.
Chain of custody	Project-dependent documentation.	Signed custody record from field to laboratory.	Continuous custody record with seals, transfer times, seal status, openings, aliquoting, and final disposition.
Destructive-analysis log	Usually implicit in laboratory records.	Recommended for non-repeatable or high-cost tests.	Required; order of analyses and consumed mass/volume must be recorded.
Blanks and contamination controls	Method-dependent.	Required for analytes prone to procedural contamination.	Required and documented, including field, equipment, trip, container, procedural, or laboratory blanks as appropriate.
Analyte-specific preservation	Based on accepted analytical method.	Required by the selected method and holding-time plan.	Required with explicit record of container material, temperature, headspace, light exposure, time to processing, and incompatibilities such as PFAS or polytetrafluoroethylene (PTFE) contact-material contamination, or microplastic contamination.
Laboratory competence	Preferred.	Appropriate accreditation or documented QA/QC is expected.	Accreditation to ISO/IEC 17025 or equivalent documented competence is strongly preferred for contested analyses.
Interpretive use	Scientific hypothesis generation and monitoring design.	Management decision support and compliance-oriented assessment.	Repeatable, auditable, independently reviewable environmental evidence; not a statement of legal admissibility.

**Table 16:** Minimum data tiers for reproducible CSTAP applications. Higher tiers can be combined with lower-tier information, but the declared tier must reflect the strongest line of evidence actually available.

Tier	Minimum Inputs	Permitted CSTAP Output	Mandatory Uncertainty Statement
Tier 0: chemical screening	$C_{i,k}^{\mathrm{tot}}$, $Q_{i}$, location, sediment unit definition.	Hazard ratios and preliminary latent activation-potential class.	Missing bioavailability, mixture, receptor, and event data must be stated explicitly.
Tier 1: geochemical modifier	Tier 0 plus grain size, TOC/OC, salinity, pH, redox proxy, and contextual hydrodynamics.	Categorical $\beta_{i,k}$ and $\alpha_{i,k,e}$; qualitative exploratory CSTAI class.	Direction and basis of geochemical corrections must be reported.
Tier 2: exposure-based	Tier 1 plus pore water, passive sampling, DGT, AVS–SEM, or contaminant-specific labile fractions.	Quantitative or semi-quantitative $\beta_{i,k}$; scenario-specific $\Psi_{k,e}$.	Analytical limits, extraction method, equilibration time, and exposure proxy must be reported.
Tier 3: effect- and event-calibrated	Tier 2 plus bioassays, benthic ecology, receptor-specific endpoints, event monitoring, or calibrated transport/reaction parameters.	Full exploratory CSTAI class, contribution shares, sensitivity outputs, and indicative follow-up class.	Calibration data, validation endpoint, and class decision rule must be provided.

**Table 17:** Evidence-adequacy grades for two-track CSTAP reporting. The grade accompanies, but does not replace, the exploratory CSTAI class.

Grade	Range of $K_{k,e}^{\mathrm{evi}}$	Name	Interpretation
A	$0.85\leq K_{k,e}^{\mathrm{evi}}\leq 1.00$	Directly supported	Most modules are constrained by direct measurements, calibrated parameters, or event observations.
B	$0.65\leq K_{k,e}^{\mathrm{evi}}<0.85$	Well supported	Core chemistry and at least one exposure or activation module are supported by measured or independently constrained inputs.
C	$0.40\leq K_{k,e}^{\mathrm{evi}}<0.65$	Partly supported	The class is interpretable as a structured hypothesis, but one or more decisive modules are proxy-based.
D	$K_{k,e}^{\mathrm{evi}}<0.40$	Screening only	The output is useful for prioritizing data acquisition but should not be used as a stand-alone management endpoint.

**Table 18:** Validation endpoints and failure criteria for CSTAP. The table converts the theoretical protocol into testable empirical claims once appropriate datasets are available.

Validation Domain	Required Observations	Expected CSTAP Behavior	Potential Falsification
Exposure prediction	Matched bulk sediment, pore water, passive-sampler, or DGT data.	Higher $\beta_{i,k}$ should correspond to higher exposure metrics after matrix effects are considered.	Predicted high bioavailability repeatedly coincides with low measured exposure under comparable conditions.
Mixture pressure	Paired contaminant mixtures and receptor-specific toxicity endpoints.	Higher $\mu_{i,k}$ or toxic-unit sums should increase adverse-effect probability when exposure is not limiting.	Mixture-adjusted scores do not improve over single-contaminant burden or produce systematic false alarms.
Event activation	Pre-/post-event chemistry, turbidity, pore water, bioassays, or benthic response.	Event scenarios with elevated $\alpha_{i,k,e}$ or $\alpha_{i,k,e}^{\mathrm{mem}}$ should show greater exposure or effect potential.	Events classified as high activation repeatedly show no exposure change despite adequate sampling.
Temporal recovery	Time series after disturbance.	Recovery should scale with $t_{\mathrm{rec},i,k}^{1/2}$ and the decay in $Z_{i,k,e}^{\mathrm{act}}\left(t\right)$.	Observed recovery is much faster or slower than predicted without an identifiable missing process.
Class calibration	Independent binary or ordinal toxicity labels.	$\varrho_{1}>0$, acceptable Brier score, and acceptable calibration error.	Calibrated probability decreases with $\Psi_{k,e}$ or remains uninformative across classes.

**Table 19:** Concise reading guide for CSTAP outputs. Only $\Psi_{k,e}$ assigns the exploratory CSTAI class; all other outputs qualify interpretation.

Output	Role	Interpretation Rule
$\Theta_{k,e}$ and $\Psi_{k,e}$	Primary toxicological-activation-potential score	Used to assign the exploratory CSTAI class under the declared site–scenario pair.
$K_{k,e}^{\mathrm{evi}}$	Evidence adequacy	Reports whether the score is supported by bulk-only data, exposure metrics, effect evidence, or event-specific observations. It does not shift the class.
$U_{k}$ and $\Pi_{k,e}^{\mathrm{prio}}$	Uncertainty-priority reporting	Helps triage data-poor cases for additional measurement. It is not toxicity evidence and does not determine the class.
${\mathrm{FIG}}_{k}$	Forensic-readiness reporting	Documents custody, duplicates, reserve aliquots, blanks, preservation, and destructive-analysis records for contested use. It does not modify the toxicological score.

**Table 20:** Minimum paired dataset for a first endpoint-specific calibration of CSTAP. The first calibration should target one primary endpoint before multiple biological outcomes are combined.

Component	Minimum Variables	Recommended First Endpoint
Chemistry and matrix	$C_{i,k}^{\mathrm{tot}}$, selected $Q_{i}$, TOC, black carbon, grain size, porosity, pH, $E^{h}$, salinity, oxygen	Required for all calibration records.
Exposure evidence	Pore water or passive samplers for hydrophobic organics; AVS–SEM or DGT for metals; route-specific metrics for PFASs and particle-associated contaminants	Paired with the same sample interval as chemistry.
Event descriptor	Baseline and post-event state, dredging/resuspension intensity, redox or oxygenation change, erosion/accessibility depth, sampling time	Needed to calibrate α rather than only burden.
Biological endpoint	Whole-sediment amphipod survival/growth or pore-water toxicity; benthic index as a separate ecological endpoint	Calibrate one primary endpoint first, then test transferability.

## Data Availability

No new experimental data were generated in this theoretical protocol-development study. The illustrative applications are based on published datasets cited in the text. The Supplementary Tables contain notation, reporting, scenario-audit, and forensic-readiness checklists. The compressed Supplementary Reproducibility Package provides the machine-readable parameter dictionary, information-source inputs, class thresholds, scenario and worked-example comma-separated-value (CSV) files, baseline-comparison table, minimal Python implementation, expected outputs, and a SHA-256 checksum manifest listed in Supplementary Table S12. The code reproduces the worked example and the core arithmetic only; optional extended modules are specified mathematically and require site-specific implementation and validation before predictive use. Further inquiries may be directed to the corresponding authors.

## Supplementary Materials

The following supporting information can be downloaded at: https://www.mdpi.com/article/10.3390/toxics14080687/s1. Supplementary Tables: Table S1, Symbol-family audit used to prevent notational ambiguity in CSTAP; Table S2, Exposure-route variables; Table S3, Additional transport and kinetic quantities; Table S4, Additional thermodynamic, oxygenation, and chemical-gate quantities; Table S5, Default exposure routes; Table S6, CSTAP reporting checklist; Table S7, Scenario definitions used for sensitivity analysis; Table S8, CSTAP stress tests; Table S9, Illustrative scenario response; Table S10, Forensic sampling and chain-of-custody metadata checklist; Table S11, Analyte-specific preservation and counter-analysis considerations; Table S12, Reproducibility package manifest; Table S13, Mapping-sensitivity alternatives for categorical 0–4 Core inputs. The CSTAP Supplementary Reproducibility Package includes comma-separated-value (CSV) inputs, class thresholds, a parameter dictionary, a minimal Python implementation, expected outputs, and a Secure Hash Algorithm 256-bit (SHA-256) checksum manifest.

## Abbreviations

AI, artificial intelligence; AVS–SEM, acid-volatile sulfide and simultaneously extracted metals; BC, black carbon; BS, Brier score; CSTAI, Coastal Sediment Toxicological Activation Index; CSTAP, Coastal Sediment Toxicological Activation Protocol; CSV, comma-separated value; DGT, diffusive gradients in thin films; ECE, expected calibration error; EqP, equilibrium partitioning; FAIR, Findable, Accessible, Interoperable, and Reusable; FIG, Forensic Integrity Grade; GPS, Global Positioning System; HCB, hexachlorobenzene; NICA, Non-Ideal Competitive Adsorption; OC, organic carbon; PAHs, polycyclic aromatic hydrocarbons; PCBs, polychlorinated biphenyls; PFASs, per- and polyfluoroalkyl substances; PNEC, predicted no-effect concentration; PTFE, polytetrafluoroethylene; QA/QC, quality assurance/quality control; SHA-256, Secure Hash Algorithm 256-bit checksum; SPM, suspended particulate matter; SQGs, sediment quality guidelines; TBT, tributyltin; TOC, total organic carbon; TU, toxic unit; VOCs, volatile organic compounds; WHAM, Windermere Humic Aqueous Model.
