# Supplementary material for: CSTAP: An Event-Conditioned Protocol for Toxicological Activation Potential in Coastal Sediments
Source: Toxics. 2026 Aug 3;14(8):687. doi: 10.3390/toxics14080687 (PMC13517519; doi:10.3390/toxics14080687)
Supplement: Supplementary file 1 [file toxics-14-00687-s001.zip › toxics-4424000-supplementary.pdf]

Supplementary Tables

CSTAP: An Event-Conditioned Protocol for Toxicological Activation Potential in Coastal Sediments

These supplementary tables contain extended notation, reporting, and scenario-audit material moved out of the main manuscript to improve readability. They are part of the reproducibility package and do not report new field measurements.

Abbreviations used in the Supplementary Tables: ASCII, American Standard Code for Information Interchange; AVS–SEM, acid-volatile sulfide and simultaneously extracted metals; CSTAI, Coastal Sediment Toxicological Activation Index; CSTAP, Coastal Sediment Toxicological Activation Protocol; DGT, diffusive gradients in thin films; FIG, Forensic Integrity Grade; OCPs, organochlorine pesticides; PAHs, polycyclic aromatic hydrocarbons; PCBs, polychlorinated biphenyls; PFAS/PFASs, per- and polyfluoroalkyl substances; QA/QC, quality assurance/quality control; SHA-256, Secure Hash Algorithm 256-bit checksum; SOPs, standard operating procedures; SVOCs, semivolatile organic compounds; VOCs, volatile organic compounds; ZIP, compressed archive file format.

Table S1. Symbol-family audit used to prevent notational ambiguity in CSTAP. Each family is reserved for a single conceptual role; related quantities are distinguished by superscripts but not repurposed across modules.

Reserved family	Conceptual role	Examples and restrictions
C	Concentrations and exposure concentrations.	$C^{\mathrm{tot}}_{i,k}$, $C^{\mathrm{pw}}_{i,k}$, $C^{\mathrm{evt}}_{i,k,e}$, and $C^{\mathrm{ceil}}_{i,k,e}$ denote concentrations or concentration ceilings only.
H	Chemical burden normalized to a benchmark.	$H_{i,k}$ is the hazard ratio and is not reused for hydrodynamic forcing, enthalpy, or class labels.
β	Bioavailability.	$\beta_{i,k}$ is the unadjusted bioavailability multiplier, and $\beta^{\mathrm{eff}}_{i,k}$ is the effective multiplier used in CSTAI after the information-source safeguard; no event, receptor, or mixture parameter uses β.
μ	Mixture pressure.	$\mu_{i,k}$ is reserved for mixture effects; the molar chemical potential is written as $\varphi^{\mathrm{chem}}_{i,k}$ to avoid conflict.
α	Activation forcing.	$\alpha_{i,k,e}$ and its constrained forms, such as $\alpha^{\mathrm{mc}}_{i,k,e}$, represent event-driven activation only.
ζ	Receptor vulnerability.	$\zeta_{i,r}$ is used only for receptor sensitivity; mobilization is denoted by $f^{\mathrm{mob}}_{i,k,e}$ rather than by a zeta-like symbol.
f and η	Mobilizable fractions and release efficiencies.	$f^{\mathrm{mob}}_{i,k,e}$ denotes the fraction of the active layer effectively mobilized; $\eta^{\mathrm{sol}}_{i,k,e}$ and $\eta^{\mathrm{pw}}_{k,e}$ denote solid-associated and porewater release efficiencies.
ξ	Dilution or exchange scaling.	$\xi^{\mathrm{dil}}_{k,e}$ denotes dilution in the event mixing volume and is not used for biological vulnerability or uncertainty.
Θ, Ψ, Ω	CSTAI score quantities.	$\Theta_{k,e}$ is the raw score, $\Psi_{k,e}$ is the log-transformed score, and $\Omega_{i,k,e}$ is a contaminant-specific contribution.

Table S2. Exposure-route variables used to connect contaminant availability with receptor-specific contact pathways. These symbols are reserved for route decomposition and are not reused elsewhere.

Symbol	Meaning	Unit	Comment
$\mathcal{G}^{\text{exp}}$	Set of exposure routes	–	Default routes are pore-water uptake, sediment ingestion, particle/contact exposure, and trophic transfer.
$\nu_{g,r}^{\text{rt}}$	Route weight for receptor r	dimensionless	Non-negative simplex weight; $\sum_{g\in\mathcal{G}^{\text{exp}}} \nu_{g,r}^{\text{rt}} = 1$.
$\mathcal{E}_{i,k,e,g}^{\text{rt}}$	Route-specific exposure metric	route-dependent	Pore-water concentration, chemical activity, labile flux, ingestible sediment burden, particle count, or tissue-transfer proxy.
$\mathcal{E}_{i,g}^{\text{rt,ref}}$	Route-specific reference exposure	same as $\mathcal{E}_{i,k,e,g}^{\text{rt}}$	Benchmark used to normalize route-specific exposure.
$A_{i,k,e,g}^{\text{rt}}$	Normalized route accessibility	dimensionless	Bounded between 0 and 1 in the default formulation.
$\kappa_{i,r,g}^{\text{rt}}$	Route-specific relative potency	dimensionless	Default value is 1 unless receptor- and route-specific evidence supports another value.
$\mathcal{V}_{i,k,e}^{\text{rt}}$	Route-resolved receptor term	dimensionless	Replaces $\mathcal{V}_{i,k}^{\text{rec}}$ in the final CSTAI calculation when route information is available.

Table S3. Additional transport and kinetic quantities used in the extended CSTAP derivation. Symbols are reserved for one meaning only throughout the manuscript.

Symbol	Meaning	Unit	Comment
$C_i^{\mathrm{ref,pw}}$	Pore-water reference concentration	mass volume^{-1}	Used in the bioavailability multiplier for contaminant i.
$C_{i,r}^{\mathrm{eff}}$	Receptor-specific effect concentration	mass volume^{-1} or compatible	Used to calculate toxic units for receptor r.
$D_{i,k}^{\mathrm{sed}}$	Effective sediment diffusion coefficient	length^2 time^{-1}	Includes tortuosity correction where appropriate.
$k_{i,k}^{\mathrm{des}}$	First-order desorption or relaxation coefficient	time^{-1}	Controls approach to local partitioning equilibrium.
$\ell_{k,e}^{\mathrm{mix}}$	Active mixed-layer length scale	length	Characteristic thickness affected by event e.
$\mathcal{J}_{i,k,e}^{\mathrm{diff}}$	Diffusive exposure flux	mass area^{-1} time^{-1}	Driven by sediment–water concentration gradients.
$\mathcal{J}_{i,k,e}^{\mathrm{res}}$	Resuspension-associated exposure flux	mass area^{-1} time^{-1}	Associated with particle erosion and pore-water release.
$\mathrm{Da}_{i,k,e}^{\mathrm{des}}$	Desorption Damköhler number	dimensionless	Ratio between event duration and desorption time scale.
$\mathrm{Pe}_{i,k,e}^{\mathrm{adv}}$	Advective Péclet number	dimensionless	Ratio between advective and diffusive transport over $\ell_{k,e}^{\mathrm{mix}}$.
$\mathrm{Bi}_{i,k,e}^{\mathrm{sw}}$	Sediment–water Biot number	dimensionless	Ratio between external exchange and internal diffusion.

Table S4. Additional thermodynamic, oxygenation, and chemical-gate quantities used in the extended CSTAP derivation.

Symbol	Meaning	Unit	Comment
$T_{k,e}^{\mathrm{abs}}$	Absolute temperature	K	Used in thermodynamic and kinetic corrections.
T_i^{ref}	Reference temperature	K	Contaminant-specific calibration temperature.
$\phi_{i,k,e}^{\mathrm{T}}$	Temperature multiplier	dimensionless	Arrhenius-type modifier for desorption or transformation rates.
$\Delta h_i^{\mathrm{sorp}}$	Apparent sorption enthalpy	energy mole^{-1}	Used for temperature-dependent partitioning when justified.
E_i^{des}	Desorption activation energy	energy mole^{-1}	Used for temperature-dependent kinetic release.
$O_{k,e}^{\mathrm{diss}}$	Dissolved oxygen	mass volume^{-1} or molarity	Oxygenation constraint on redox-sensitive activation.
$I_{k,e}^{\mathrm{ion}}$	Ionic strength	molarity or dimensionless proxy	Used for salinity and PFAS/ionic-contaminant partitioning.
$\mathfrak{a}_{i,k,e}$	Chemical activity	dimensionless	Activity of the bioavailable species or exposure-driving phase.
$\Delta^{\star}_{\mathrm{pH},k,e}$, $\Delta^{\star}_{\mathrm{sal},k,e}$, $\Delta^{\star}_{E^{\mathrm{h}},k,e}$	Standardized scenario deviations	dimensionless	pH, salinity, and redox departures scaled by reference intervals.
$\mathcal{Z}_{i,k,e}^{\mathrm{act}}(t)$	Activation-memory state	dimensionless	Bounded state variable representing persistence of event-driven activation through time.
$k_{i,k}^{\mathrm{act}}$	Activation-rate coefficient	time^{-1}	Rate at which normalized event forcing increases activation memory.
$k_{i,k}^{\mathrm{rec}}$	Recovery-rate coefficient	time^{-1}	Rate at which activation memory decays after forcing ceases.
$q_{i,k,e}^{\mathrm{evt}}(t)$	Normalized event-forcing intensity	dimensionless	Scaled contaminant–site–scenario hydrodynamic, redox, mechanical, or biogeochemical forcing entering the memory equation.

Table S5. Default exposure routes used in the route-resolved receptor layer. The table is a reporting aid; each route must still be supported by measured data, a transparent proxy, or an explicit uncertainty statement.

**Route**	**Possible exposure metric**	**Typical activation mechanism**	**Receptor relevance**
Pore-water uptake	Interstitial-water concentration, freely dissolved concentration, chemical activity, DGT-labile flux.	Desorption, sulfide oxidation, redox switch, pH or salinity shift, pore-water expulsion.	Amphipods, polychaetes, bivalves, embryos, and larval stages.
Sediment ingestion	Ingestible fine-fraction burden, gut-fluid extractable fraction, deposit-feeding rate proxy.	Resuspension, surface exposure of buried layers, bioturbation, dredging-induced mixing.	Deposit-feeding invertebrates and demersal organisms.
Particle/contact exposure	Suspended particulate concentration, microplastic or nanoplastic particle number, particle-bound toxic units.	Storm waves, dredging plumes, flood pulses, settling-resuspension cycles.	Filter feeders, suspension feeders, early life stages, and benthic epifauna.
Trophic transfer	Tissue or food-web proxy, bioaccumulation factor, biomagnification-relevant concentration.	Long-term mobilization, prey contamination, sediment-to-biota transfer.	Bivalve harvesting areas, nursery habitats, demersal fish, and higher trophic levels.

Table S6. CSTAP reporting checklist for future applications.

Item	Required reporting element
1	Spatial unit k, sediment depth interval, grain-size fraction, and dry-weight normalization.
2	Contaminant list, analytical method, detection limits, and treatment of censored values.
3	Benchmark source $\mathcal{Q}_i$ and rationale for background correction or site-specific threshold selection.
4	Exposure proxy used for $\mathcal{E}_{i,k}$, $\beta_{i,k}$, $\mathcal{I}^{\mathrm{src}}_{i,k}$, and $\beta^{\mathrm{eff}}_{i,k}$: measured pore water, passive sampler, DGT, AVS–SEM, modeled pore water, or categorical proxy.
5	Mixture assumption: concentration addition, independent action, interaction matrix, or conservative no-interaction default.
6	Scenario definition e, including event duration, bed-shear or dredging descriptor, pH, salinity, redox, hypoxia assumptions, and any activation-memory or recovery parameters.
7	Receptor set, weights $\lambda^{\mathrm{rec}}_{r,k}$, vulnerability coefficients $\zeta_{i,r}$, and ecological justification.
8	Uncertainty factor $\mathcal{U}_k$, evidence adequacy $\mathfrak{K}^{\mathrm{evi}}_{k,e}$, missing variables, sensitivity outputs, and class threshold used.
9	Dominant contribution shares $q^{\mathrm{con}}_{i,k,e}$ and whether the class is burden-driven, bioavailability-driven, mixture-driven, or activation-driven.
10	Recommended follow-up measurement or management-review step required to move the application to the next data tier.

Table S7. Scenario definitions used for sensitivity analysis. Values are dimensionless theoretical settings for protocol testing.

**Scenario**	**Dominant forcing**	**Primary affected terms**	**Expected CSTAP response**
Baseline oxic stability	Low shear stress, stable redox, no major works	$\mathcal{I}^{\mathrm{phys}}_{k,e} \approx 0$, $\mathcal{I}^{\mathrm{chem}}_{i,k,e} \approx 0$, $\mathcal{I}^{\mathrm{bio}}_{k,e} \approx 0$, $\mathcal{I}^{\mathrm{act}}_{i,k,e} \approx 0$	Score governed mainly by chemical burden and baseline bioavailability.
Dredging/resuspension	Mechanical erosion and suspended sediment plume	$\delta^{\mathrm{drg}}_{k,e}$, $\tau_{\mathrm{b},k,e}$, porewater release	Increased dissolved and particle-associated exposure; higher $\alpha_{i,k,e}$.
Hypoxia/anoxia	Oxygen depletion and redox lowering	$\Delta^{\star}_{E^{\mathrm{h}},k,e} < 0$, sulfide formation or metal release depending phase	Strong contaminant-specific response; potential release of redox-sensitive elements.
Storm/flood runoff	High flow, wave action, new contaminant inputs	$\tau_{\mathrm{b},k,e}$, external loading, salinity shift	Pulsed resuspension and mixing; elevated activation for fine and contaminated deposits.
Acidification/salinity shift	Lower pH, ionic-strength change	$\Delta^{\star}_{\mathrm{pH},k,e}$, $\Delta^{\star}_{\mathrm{sal},k,e}$	Increased lability for pH-sensitive metals and altered partitioning for PFAS/organics.
Combined climate disturbance	Co-occurrence of storm, warming, hypoxia, acidification	Multiple multipliers simultaneously	Highest probability of class shift due to coupled activation mechanisms.

Table S8. CSTAP stress tests used to verify mathematical and physical behavior in limiting cases.

**Condition**	**Expected model behavior**	**Interpretation**
Zero chemical burden	$H_{i,k} = 0$ for all i implies $\Theta_{k,e} = 0$	Disturbance alone cannot generate contaminant toxicity.
High burden, no independent bioavailability amplification	Elevated $H_{i,k}$ with $\beta^{\mathrm{eff}}_{i,k} = 1$ and low $\mathcal{A}^{\mathrm{z}}_{k,e}$ yields a storage-dominated protocol signal	Storage is separated from exposure in conservative screening mode.
High disturbance, low burden	High $\alpha_{i,k,e}$ with negligible $H_{i,k}$ does not create a high exploratory CSTAI class	Physical hazard and contaminant toxicity are not conflated.
Single-contaminant dominance	$q^{\mathrm{con}}_{j,k,e} \to 1$ for contaminant j	The model remains interpretable as a single-contaminant assessment because one contribution share dominates the score.
Sparse evidence	$\mathcal{U}_k$ and $\mathfrak{K}^{\mathrm{evi}}_{k,e}$ flag weak support while the declared tier remains low	Uncertainty is reported as uncertainty, not as confirmed toxicity.

Table S9. Illustrative scenario response for a theoretical mixed-contaminant coastal sediment. Values are log-transformed CSTAI scores intended to illustrate protocol behavior, not to represent a particular measured site.

**Scenario**	**Illustrative** $\Psi_{k,e}$	**Class**	**Interpretation**
Baseline oxic stability	0.82	III	Elevated latent activation potential driven by chemical burden and moderate bioavailability.
Dredging/resuspension	1.34	IV	Mechanical disturbance increases pore-water release and particle contact.
Hypoxia/anoxia	1.08	III–IV	Redox-sensitive response; class depends on metal sulfide balance and organic matter degradation.
Storm/flood runoff	1.22	IV	Resuspension and new contaminant inputs raise activation potential.
Acidification/salinity shift	1.15	IV	pH- and salinity-sensitive contaminants become more labile.
Combined climate disturbance	1.67	V	Multiple triggers coincide, producing a high-priority protocol signal that requires confirmatory assessment.

Table S10. Forensic sampling and chain-of-custody metadata checklist for contested CSTAP applications. The checklist supports FIG assignment and does not modify CSTAI.

**Metadata group**	**Minimum fields to record**
Sample identity	Unique sample code, project/case code, matrix, sediment unit, core interval or surface layer, container code, seal code.
Location and depth	Latitude, longitude, coordinate system, water depth, sediment depth interval, tidal state if relevant, field sketch or station map.
Field conditions	Date, time, operators, weather, sea state, recent disturbance, visible oil/debris/odor, photographs, deviations from sampling plan.
Sampling method	Device, grab/core type, penetration depth, recovery length, disturbed/undisturbed status, compositing decision, decontamination procedure.
Duplicate and reserve design	Field duplicate identifier, split duplicate identifier, analytical aliquot, reserve aliquot, archive core or half-core, mass/volume reserved.
Preservation and transport	Container material, headspace, light protection, temperature, cooling/freezing decision, time to laboratory, holding-time target, analyte-specific incompatibilities.
Custody events	Person releasing, person receiving, date/time of transfer, seal status, storage condition, shipping record, laboratory receipt condition.
Destructive-analysis record	Aliquot consumed, mass/volume consumed, extraction/digestion/elutriation/bioassay step, remaining reserve material, whether counter-analysis remains possible.
QA/QC controls	Field blank, equipment blank, trip blank, container blank, procedural blank, certified/reference material, laboratory duplicate, method blank.

Table S11. Analyte-specific preservation and counter-analysis considerations for contested-use CSTAP applications. These are planning principles and do not replace method-specific SOPs.

**Target class**	**Container/preservation concern**	**Forensic repeatability concern**	**Counter-analysis note**
Total metals and metalloids	Use clean containers compatible with trace-metal analysis; avoid cross-contamination and moisture loss where water content is needed.	Digestion is destructive; split duplicate and reserve aliquot should be created before digestion.	Preserve enough dry or wet mass for independent digestion and reference-material comparison.
Redox-sensitive species	Minimize oxygenation changes when speciation, methylation, sulfides, or pore-water chemistry are endpoints.	pH, E^{h}, sulfide, and pore-water separation can change after sampling.	Record time to measurement, atmosphere/contact with air, and whether reserve material can reproduce original redox state.
PAHs, PCBs, OCPs, and petroleum residues	Prefer containers compatible with organic analysis; minimize light, heat, volatilization, and sorption losses.	Solvent extraction is destructive and homogenization can obscure stratigraphic evidence.	Archive pre-extraction material and document compositing or homogenization order.
VOCs/SVOCs	Minimize headspace and disturbance; use dedicated containers and trip blanks where applicable.	Volatilization can make repeated analysis non-equivalent.	Reserve design must be analyte-specific; a generic archive aliquot may be inadequate.
PFASs	Avoid fluorinated materials and document all sampling and laboratory contact materials.	Low-level contamination from containers, tubing, clothing, or field materials can dominate results.	Field, equipment, and container blanks are central to contested interpretation.
Microplastics/nanoplastics	Minimize airborne fibres and plastic contamination; document clothing, tools, and procedural blanks.	Digestion and density separation are destructive and can alter particle counts or morphology.	Preserve an unprocessed aliquot and record polymer-analysis method, particle-size window, and blank correction.
Pore water and elutriates	Separation and elutriation alter the original sediment matrix and can be time-sensitive.	The analysed phase may not be reproducible unless separation conditions are replicated.	Record centrifugation/filtration, redox exposure, salinity, temperature, contact time, and dilution.
Bioassays	Test preparation consumes or transforms material and may require specific storage conditions.	Biological response can depend on holding time, oxygenation, salinity, and handling history.	Preserve matched sediment for chemistry and counter-analysis; record organism, endpoint, exposure duration, and acclimation conditions.

Table S12. Reproducibility package manifest. The file names below match the internal paths in the compressed Supplementary Reproducibility Package.

**File path in ZIP**	**Purpose**
`cstap_reproducibility/README_CSTAP_reproducibility.md`	Execution instructions, scope statement, ASCII alias definitions, and statement that the code reproduces the worked example and core arithmetic only.
`cstap_reproducibility/baseline_comparison_logic.csv`	Baseline-comparison descriptions for bulk, exposure, toxic-unit, and event-free CSTAP core outputs.
`cstap_reproducibility/beta_isrc_examples.csv`	Minimal examples for assigning $\mathcal{I}^{\mathrm{src}}$ and computing β^{eff} under bulk-only, partitioning-derived, and measured-exposure conditions.
`cstap_reproducibility/class_thresholds.csv`	Provisional exploratory class thresholds on the Ψ scale.
`cstap_reproducibility/cstap_minimal_implementation.py`	Standard-library Python script reproducing the worked example and core arithmetic; includes optional `--check` comparison against expected output.
`cstap_reproducibility/forensic_integrity_checklist.csv`	Checklist supporting the optional forensic-readiness reporting layer.
`cstap_reproducibility/generated_outputs/qc_gate_results.csv`	Quality-control checks generated by the minimal implementation for the worked example.
`cstap_reproducibility/generated_outputs/worked_example_outputs_ generated.csv`	Output generated by the minimal implementation during the reproducibility run.
`cstap_reproducibility/governance_rules.csv`	Operational rules for $\mathcal{I}^{\mathrm{src}}$, β^{eff}, the ASCII alias `V_rt` for $\mathcal{V}^{\mathrm{rt}}$, $\mathcal{U}$, and $\mathfrak{K}^{\mathrm{evi}}$.
`cstap_reproducibility/information_source_inputs.csv`	Component-level evidence sources and information-source coefficients used in the worked example.
`cstap_reproducibility/minimum_validation_dataset.csv`	Machine-readable form of the minimum paired dataset recommended for first endpoint-specific calibration.
`cstap_reproducibility/parameter_dictionary.csv`	Machine-readable dictionary of symbols, inputs, units, default status, ASCII aliases, and notes.
`cstap_reproducibility/parameter_governance_extended.csv`	Default, range, and evidence requirements for weakly identified parameters.
`cstap_reproducibility/repro_run.log`	Console output documenting successful execution of the worked example without temporary absolute paths.
`cstap_reproducibility/run_all.sh`	Convenience shell script running the worked example, Python tests, and SHA256 manifest verification.
`cstap_reproducibility/scenario_inputs.csv`	Scenario descriptors and mass-ceiling applicability flags for illustrative events.
`cstap_reproducibility/test_repro.py`	Small Python test file checking zero burden, β^{eff}, thresholds, contribution shares, and manifest integrity.
`cstap_reproducibility/threshold_theta_equivalents.csv`	Raw Θ equivalents of the Ψ thresholds.
`cstap_reproducibility/worked_example_expected_outputs.csv`	Fixed expected output table used by the `--check` option.
`cstap_reproducibility/worked_example_inputs.csv`	Input table for the dimensionless worked example.
`cstap_reproducibility/categorical_mapping_sensitivity.csv`	Alternative bounded ordinal mappings and reporting triggers used to test the robustness of categorical Core inputs.
`cstap_reproducibility/MANIFEST_SHA256.txt`	File names and SHA-256 checksums for integrity verification.

Table S13. Mapping-sensitivity alternatives for categorical 0–4 Core inputs. These mappings are protocol–governance checks, not calibrated biological response functions. Category 0 remains 0 for burden and 1 for neutral process multipliers.

**Category**	**Meaning**	**Compressed mapping**	**Default mapping**	**Linear-to-cap mapping**
0	absent/not applicable	0 burden or 1.00 neutral	0 burden or 1.00 neutral	0 burden or 1.00 neutral
1	low	1.00	1.00	1.00
2	moderate	1.10	1.25	1.33
3	high	1.25	1.50	1.67
4	very high	1.50	2.00	2.00

Sensitivity rule. Apply at least the default and compressed mappings when a categorical module contributes $\geq 25\%$ of $\Theta_{k,e}$ or when $\Psi_{k,e}$ lies within 0.10 of a class boundary. The linear-to-cap mapping provides an additional rank-preserving check. If the exploratory class changes under an admissible alternative mapping, report all resulting classes and label the application *mapping-sensitive*. No operational inference should be based on the categorical class alone in that case. Measured or endpoint-calibrated process functions supersede these mappings.
